# Why are so many enteric pathogen infections asymptomatic? Pathogen and gut microbiome characteristics associated with diarrhea symptoms and carriage of diarrheagenic *E. coli* in northern Ecuador

**DOI:** 10.1080/19490976.2023.2281010

**Published:** 2023-11-22

**Authors:** Kelsey J Jesser, Gabriel Trueba, Konstantinos T. Konstantinidis, Karen Levy

**Affiliations:** a Department of Environmental and Occupational Health Sciences, University of Washington, Seattle, WA, USA; b Instituto de Microbiología, Universidad San Francisco de Quito, Quito, Ecuador; c School of Civil and Environmental Engineering and School of Biological Sciences, Georgia Institute of Technology, Atlanta, GA, USA

**Keywords:** Diarrhea, e. coli, gut microbiome, pathogen, symptomatic, asymptomatic

## Abstract

A high proportion of enteric infections, including those caused by diarrheagenic *Escherichia coli* (DEC), are asymptomatic for diarrhea. The factors responsible for the development of diarrhea symptoms, or lack thereof, remain unclear. Here, we used DEC isolate genome and whole stool microbiome data from a case–control study of diarrhea in Ecuador to examine factors associated with diarrhea symptoms accompanying DEC carriage. We investigated i) pathogen abundance, ii) gut microbiome characteristics, and iii) strain-level pathogen characteristics from DEC infections with diarrhea symptoms (symptomatic infections) and without diarrhea symptoms (asymptomatic infections). We also included data from individuals with and without diarrhea who were not infected with DEC (uninfected cases and controls). i) *E. coli* relative abundance in the gut microbiome was highly variable, but higher on-average in individuals with symptomatic compared to asymptomatic DEC infections. Similarly, the number and relative abundances of virulence genes in the gut were higher in symptomatic than asymptomatic DEC infections. ii) Measures of microbiome diversity were similar regardless of diarrhea symptoms or DEC carriage. Proteobacterial families that have been described as pathobionts were enriched in symptomatic infections and uninfected cases, whereas potentially beneficial taxa, including the *Bacteroidaceae* and *Bifidobacteriaceae*, were more abundant in individuals without diarrhea. An analysis of high-level gene functions recovered in metagenomes revealed that genes that were differentially abundant by diarrhea and DEC infection status were more abundant in symptomatic than asymptomatic DEC infections. iii) DEC isolates from symptomatic versus asymptomatic individuals showed no significant differences in virulence or accessory gene content, and there was no phylogenetic signal associated with diarrhea symptoms. Together, these data suggest signals that distinguish symptomatic from asymptomatic DEC infections. In particular, the abundance of *E. coli*, the virulence gene content of the gut microbiome, and the taxa present in the gut microbiome have an apparent role.

## Introduction

Infectious diarrhea is a leading contributor to mortality and morbidity globally, particularly in low- and middle-income countries (LMICs), where there is limited access to water and sanitation services^1^. Diarrhea is responsible for an estimated 1.6 million deaths each year, of which nearly a half-million occur in children under age 5.^2^ In addition, diarrhea has been linked to growth faltering, malnutrition, and other morbidities in young children.^2,3^ Though the incidence and risk of mortality associated with diarrhea declines incrementally with age, diarrhea in older children and adults is associated with dehydration, increased incidence of inflammatory bowel diseases, and loss of income and education.^4,5^ However, not all enteropathogen infections lead to diarrhea, and many enteropathogens that have been associated with diarrhea symptoms demonstrate asymptomatic colonization and shedding.^6^

Of the wide array of bacterial, viral, and parasitic etiological agents that cause infectious diarrhea, diarrheagenic strains of the bacterium *Escherichia coli* are among the most common and widely studied^7^. Though typically a benign member of the commensal gut microbiota, *E. coli* can acquire virulence genes that enable it to cause diarrheal and extraintestinal disease.^8^ *E. coli* strains that can cause diarrhea in humans are collectively referred to as diarrheagenic *E. coli* (DEC) and are grouped into pathotypes based on the presence of specific virulence genes. DEC pathotypes include: enterotoxigenic *E. coli* (ETEC), typical and atypical enteropathogenic *E. coli* (tEPEC and aEPEC, respectively), enterohemmorhagic *E. coli* (EHEC), enteroinvasive *E. coli* (EIEC), enteroaggregative *E. coli* (EAEC), and diffusely adherent *E. coli* (DAEC). While all of these DEC pathotypes have been associated with diarrhea in epidemiological and/or challenge studies,^7,9^ they have also all been isolated from individuals who are asymptomatic for diarrhea.^7,10–13^

Numerous research efforts have highlighted the prevalence of asymptomatic carriage of DEC and other enteropathogens in LMIC settings. A recent meta-analysis that estimated odds ratios between pathogen detection and diarrhea for common enteropathogens, including DEC pathotypes ETEC and EPEC, concluded that nearly all enteropathogens demonstrate asymptomatic carriage and associations between pathogen detection and diarrhea vary by age and child mortality setting.^14^ Two large-scale studies of childhood diarrhea in LMIC settings, the Global Enterics Multi-Center Study (GEMS)^15^ and the Malnutrition and Enteric Disease Study (Mal-ED),^16^ reported high rates of asymptomatic enteric infection by DEC and other enteropathogens in young children and infants. The frequent asymptomatic colonization of the gastrointestinal tract by DEC pathotypes has influenced the scientific and clinical understanding of these organisms as diarrheal disease agents.^6,7^

A combination of host, pathogen, and environmental factors are thought to determine whether a given enteric infection results in acute diarrhea. These include: strain-level heterogeneity in pathogen virulence, the amount of inoculum ingested by the host, pathogen incubation and shedding rates, host immune status and susceptibility to infection, and the structure and function of the host commensal microbiome.^6^ The relative importance of these factors in determining symptomatic or asymptomatic disease outcomes remains unclear, but whole-genome and community-level gut microbiome sequencing methods hold promise for addressing which are most critical. Whole-genome sequence-based analyses of DEC and other enteropathogens isolated from symptomatic and asymptomatic individuals may be used to elucidate whether virulence genes or other genome characteristics are associated with diarrheal disease outcomes. This is important because DEC pathotypes are composed of heterogenous strains that encompass multiple lineages, some with greater virulence potential than others.^17,18^ Amplicon and/or shotgun metagenome sequencing can provide insights into if and how the taxonomic composition and the virulence and metabolic potential of the entire gut microbiome affect symptomology accompanying a DEC infection.^7,19^

Diagnosis and treatment of infectious diarrhea is typically determined by the detection of a given pathogen from diarrheal stool.^9^ The frequency of asymptomatic enteropathogen carriage has become more apparent as detection of enteropathogens has increasingly relied on molecular diagnostics, which offer improved sensitivity compared to culture-based methods.^20,21^ Molecular methods that quantify pathogen-derived nucleic acids in a sample (*e.g.*, quantitative PCR (qPCR)) have also led to the discovery that enteropathogen abundance in the gut is positively associated with diarrhea symptoms.^20,22^ However, this approach still relies on pathogen detection and does not account for the gut microbiome or host-associated factors that may influence the development of diarrhea. Identification of which, if any, additional factors are associated with symptomatic infections could help to define additional screening criteria for diagnosis and treatment of enteric infections in LMIC settings.

Here, we used gut microbiome and DEC isolate genome data from EcoZUR, an age-matched case–control study of diarrhea conducted in northern Ecuador, to gain insights into the pathogen and gut microbiome factors associated with diarrhea symptoms and DEC carriage. This unique dataset combines whole-genome sequencing data for DEC pathotypes isolated from diarrhea cases and controls with gut microbiome sequencing data for the same sample set, enabling comparison of pathogen and gut microbiome characteristics in individuals with symptomatic versus asymptomatic DEC infections. We also considered diarrhea case and non-diarrhea control samples from individuals where we did not detect DEC by isolation for comparison, which allowed us to explore how factors that may be associated with diarrhea are impacted by DEC carriage. We investigated: i) pathogen abundance, as determined by the amount of *E. coli* and total number and relative abundances of virulence genes in the gut, ii) gut microbiome characteristics, as determined by the diversity, taxonomic composition, and functional potential of the gut microbiome, and iii) pathogen characteristics, as determined by DEC strain relatedness and functional and virulence traits.

## Methods

### Study design and case/control definitions

The EcoZUR study was conducted in northern Ecuador from April 2014 to September 2015. Participants were recruited from Ecuadorian Ministry of Health hospitals and/or clinics as previously described.^23^ All study participants signed a consent approved by the Emory University Institutional Review Board (IRB00065781) and the Universidad San Francisco de Quito (USFQ 2013-145 M), and the research protocol was approved by the Ecuadorian Ministry of Health (MSP-DIS-204-005-O). In total, 907 participants were enrolled and submitted survey data on demographic variables, including age, race, and antibiotic usage. Of these, *n=*771 participants submitted stool samples.

**Figure 1.**
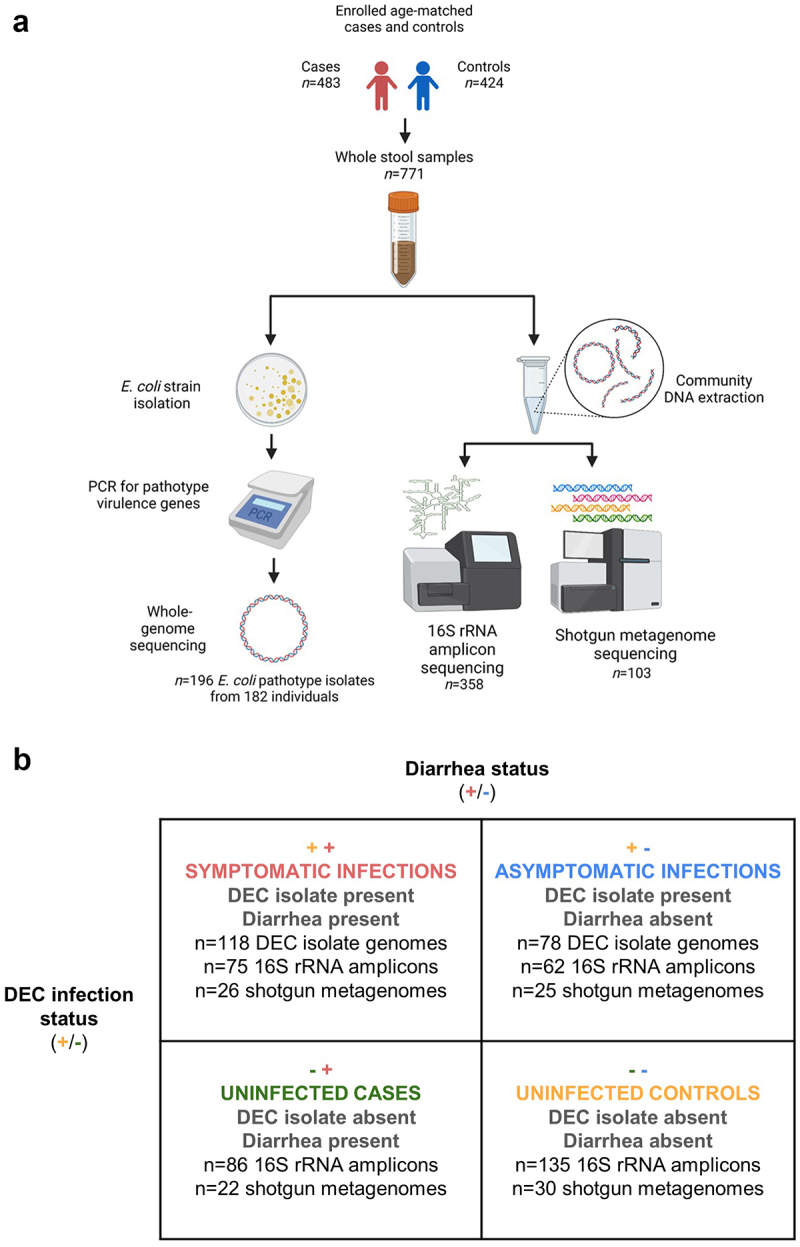
Summary of study design (a) and sample groups (b). All participant stool samples were PCR-screened for DEC pathotype genes and positive isolates were whole-genome sequenced. A random subset of age and sex-matched participant stool samples were selected for 16S rRNA gene amplicon gut microbiome sequencing, and a further subset were selected for shotgun metagenome sequencing so that there would be an approximately equal number of shotgun metagenomes for diarrheal case and control samples and DEC-positive and negative samples. Symbols in panel b indicate diarrhea case (+) or control (-) status and the presence (+) or absence (-) of DEC in participant stools. All samples with a DEC isolate genome are from infections because by-definition there were no DEC isolates detected in the uninfected stool samples. See text and Supplementary Table 2 for additional sample selection and filtering criteria.

We analyzed samples from four different groups of participants: a) diarrhea cases with DEC infections (symptomatic infections), b) non-diarrhea controls with DEC infections (asymptomatic infections), c) uninfected diarrhea cases, and d) uninfected non-diarrhea controls. DEC infections were determined by DEC isolation and identification using endpoint PCR and whole-genome typing, as described in detail below. Cases were defined as individuals of any age visiting a hospital or clinic with acute diarrhea (>3 loose stools in 24 h), and controls were age-matched individuals visiting the same clinic for another complaint without diarrhea, vomiting, or antibiotic use in the prior week. Case/control matching was robust on key demographic variables, including age, sex, and race^23^. Stool samples were subjected to a variety of analyses encompassing *E. coli* isolation and screening for DEC pathotype diagnostic genes (all samples), ELISA screening for rotavirus infection (all samples), whole-genome sequencing of DEC isolates (all samples), 16S rRNA gene amplicon sequencing of whole stool (random subset of age and sex-matched samples), and short-read shotgun metagenomic sequencing of whole stool (age and sex-matched samples selected based on case-control and DEC infection status). The study design, laboratory methods, and sample groups are summarized in Figure 1. Additional details about the design of the EcoZUR study have been reported elsewhere.^23^

### E. coli *isolation and pathogen detection*

Fresh stools were collected and used to isolate DEC as previously reported.^23^ After testing to differentiate Shigellae, endpoint PCR (assays listed in Supplementary Table S1) was used to screen isolate lysates for DEC pathotype diagnostic virulence genes. The PCR-based pathotype designations were later confirmed using an isolate whole-genome scan for diagnostic genes. Detailed methods for DEC isolation, endpoint PCR, and the whole-genome scan for DEC diagnostic genes are available in the Supplementary Material.

Individuals were determined to have a DEC infection if an *E. coli* isolate with a diagnostic virulence gene profile (as determined by both the PCR and genome-based pathotype analyses) was isolated from their stool. We also analyzed data for uninfected cases and controls where no DEC isolate was detected to better understand how DEC carriage impacts diarrhea symptom development in comparison to symptoms from undiagnosed infectious agents or by noninfectious osmotic or food-related factors. In addition to DEC and Shigellae, fresh stool samples were tested for rotavirus using a RIDA Quick Rotavirus test (r-Biopharm, Darmstadt, Germany). Rotavirus-positive samples were filtered from the dataset prior to downstream bioinformatic analyses because rotavirus is consistently strongly associated with diarrhea symptoms.^14^ In the EcoZUR study, 202 of 204 total rotavirus detections (99%) were in individuals who were symptomatic for diarrhea. Cases of diarrhea with rotavirus infections were therefore presumed to be caused by rotavirus and removed from the analysis to allow for a focus on the impact of DEC.

### DNA extraction and sequencing

Presumptive DEC isolates and whole stool samples were transported in liquid nitrogen dewars and stored at −80°C at the Universidad San Francisco de Quito. DNA from isolates and whole stool samples were extracted and sequenced as described in the Supplementary Material. Sequencing data were subject to additional filtering criteria as described below prior to inclusion in the analyses for this study, resulting in a final dataset of *n=*196 DEC isolate genomes, *n=*358 16S rRNA gene amplicon communities, and *n=*103 whole shotgun metagenomes (Figure 1, Supplementary Table 2). All sequencing data is publicly available under NCBI BioProject PRJNA486009, and associated metadata and sequencing/assembly metrics are listed in the Supplementary Material (Supplementary Tables 3–6). DEC strain serotypes and pathotype associations with diarrhea in the study area have been previously reported for this dataset.^24,25^

### Sequence processing and sample filtering

#### Isolate genomes

Isolate whole-genome sequencing reads were filtered, assembled, and annotated using the Microbial Genomes Atlas (MiGA) bioinformatics pipeline for microbial genomes and metagenomes.^26^ Within MiGA, reads were trimmed and quality filtered using SolexaQA++^27^, then assembled *de novo* using IDBA-UD with precorrections.^28^ Protein-coding gene regions were annotated using MetaGeneMark, and taxonomy was assigned to isolate assemblies based on average nucleotide identity (ANI) and average amino acid identity (AAI) comparisons to a reference database. MyTaxa scan barplots^29^ and MiGA reports of genome contamination and completeness based on the recovery of lineage-specific marker genes were used to confirm the taxonomic assignment and assess quality of each assembly.

PCR-based virulence gene detections and DEC pathotype designations for *E. coli* isolates were confirmed using a read-mapping-based analysis of diagnostic virulence genes. See the Supplementary Material for detailed methods and Supplementary Table 7 for reference gene sequences and pathotype designation criteria. Of *n* = 279 total *E. coli* isolates designated as DEC by PCR, 83 were filtered from the analysis because the participant stool was rotavirus-positive, whole-genome sequences were poor-quality or had ambiguous taxonomic assignments, or DEC pathotype designations by PCR and whole-genome sequencing were mismatched. This resulted in a final dataset of *n* = 196 pathotype isolates from *n =* 182 individuals (Supplementary Table 2).

#### 16S rRNA gene amplicons

Demultiplexed 16S rRNA gene amplicon libraries were processed using QIIME2 v. 2019.7 (https://qiime2.org/). Forward and reverse amplicon sequences were denoised, trimmed, quality filtered, and clustered into amplicon sequence variants (ASVs) using Dada2^30^ as implemented in QIIME2. A feature table containing read counts for ASVs that were 100% identical was generated, and ASVs were assigned taxonomy using the q2-feature-classifier^31^ classify-sklearn naïve Bayes taxonomy classifier trained on the GreenGenes 13_8 reference database.^32^ ASVs were filtered to remove ambiguous and chloroplast and mitochondria-annotated sequences using the R package phyloseq.^33^ After removal of *n=*53 samples that were rotavirus-positive, had insufficient or missing metadata, or where infection status could not be determined due to contamination of the isolate sequence (Supplementary Table 2), there were *n* = 358 samples for which 16S rRNA gene amplicon data was analyzed.

#### Shotgun metagenomes

Raw shotgun metagenome reads were mapped against the human reference genome sequence using BMTagger,^34^ and human-associated reads were removed prior to downstream analyses. Human-decontaminated reads were trimmed, quality filtered, and assembled into contigs using MiGA as described above for isolate whole-genome sequences. The taxonomic composition of shotgun metagenomes was determined using Kraken 2,^35^ which applies a k-mer based approached to classify short metagenome reads. We used the “Standard” RefSeq database (available at https://benlangmead.github.io/aws-indexes/k2) to run Kraken 2, which includes references sequences from archaea, bacteria, viruses, plasmids, and humans. Bracken^36^ was used to generate abundance estimates from Kraken 2 taxonomic annotations. Normalized abundances for each taxon in each shotgun metagenome were calculated as the number of annotated divided by total trimmed reads. Of *n=*108 total shotgun metagenomes sequenced, four were positive for rotavirus and one had a contaminated isolate genome sequence. These samples were filtered from the analysis (Supplementary Table 2), resulting in *n=*103 shotgun metagenomes for downstream analyses.

### Pathogen abundance

#### Metagenome abundance of DEC isolates

*In situ* relative abundance of DEC isolate genome sequences was calculated by aligning human read-filtered and quality-trimmed metagenome reads to the assembly of the DEC isolate recovered from the corresponding sample (*n=*53). Because DEC isolates were isolated only from infected individuals, no data from uninfected individuals was included in this analysis. Shotgun metagenome reads were aligned to DEC isolate genome assemblies with ≥95% nucleotide identity and ≥70% query sequence coverage using blastn. TAD80 values (truncated average sequence depth across the genome after removing the top and bottom 10% of genome positions) were calculated from the resulting alignments using enveomics scripts^37^ and genome equivalent values for each metagenome were calculated using MicrobeCensus.^38^ Metagenome relative abundances were estimated using the ratio of the TAD80 of the query genome to MicrobeCensus genome equivalents in the corresponding metagenome as previously described^19^. Shapiro-Wilkes testing revealed data were non-normal, so we used nonparametric Wilcoxon tests to compare metagenome DEC abundances in samples from symptomatic versus asymptomatic individuals with a significance threshold of *p* < 0.05. These and all other statistical analyses were conducted in R v.3.6.3.^39^ In addition to conducting this analysis for all samples, we conducted a sub-analysis for children age 5 years and younger, given the gut microbiome differences and disproportionate impact of enteropathogen infection and diarrhea on young children^2^.

#### E. coli abundance by qPCR

qPCR assays for total *E. coli uidA* and total bacterial 16S rRNA genes were run for a subset of *n =* 34 samples for which sufficient whole stool nucleic acid template was available after 16S rRNA gene amplicon and shotgun metagenome sequencing; qPCR methods are detailed in the Supplementary Material and assay details are listed in Supplementary Table 8. Percent *E. coli* in total community DNA was calculated by qPCR as the abundance of *E. coli uidA* copies per ng DNA divided by the abundance of total bacterial 16S rRNA gene copies per ng DNA and compared to the metagenome relative abundances of DEC isolate sequences using Spearman’s rho correlations.

#### Number and relative abundance of virulence genes in the gut microbiome

Filtered metagenome reads were mapped against the Virulence Factors Database (VFDB; http://www.mgc.ac.cn/VFs/, accessed January 21, 2021)^53^ using blastn^40^ following the approach described in the Supplementary Material for DEC pathotype genes. Blastn outputs were filtered to include only the best match for each read. A gene was considered present in the metagenome if it had a sequencing depth >1X and >70% gene length coverage, and absent if it had lower depth or coverage (note that read coverage of the housekeeping gene *rpoB* in isolates was > 20X (26.1X coverage on average)). Relative abundance for each present gene was calculated as gene sequencing depth divided by the MicrobeCensus genome equivalent value for the corresponding metagenome. After checking for normality with Shapiro-Wilkes testing, we used nonparametric Wilcoxon rank sum tests with a significance threshold of *p* < 0.05 to determine whether the number of total or *E. coli*-annotated virulence genes differed for symptomatic versus asymptomatic DEC infections or uninfected cases versus controls. In addition, we conducted the same comparisons for *Salmonella*, *Campylobacter*, and *Klebsiella*-annotated virulence genes to determine whether there was evidence for diarrhea symptoms associated with carriage of other common bacterial enteropathogens. VFDB genes present in >10% of metagenomes were tested for significantly different relative abundances between symptomatic DEC infections, asymptomatic DEC infections, uninfected cases, and uninfected controls using a four-way Kruskal–Wallis nonparametric test with a Benjamani-Hochberg (BH) multiple-test correction. Virulence genes were considered significantly differentially abundant between groups if they had a BH-adjusted p-value <0.05. Heatmaps of mean relative abundance of significant virulent genes were made using ComplexHeatmap^41^ with default hierarchical clustering. As for DEC isolate abundance, we also conducted testing for differences in the number and relative abundance of virulence genes after subsetting the data to include only samples from participants aged less than 5 years.

### Gut microbiome characteristics

#### Diversity metrics

Alpha-diversity metrics (observed ASVs (species richness), Shannon and Simpson diversity) were estimated for 16S rRNA gene amplicon data using the R package iNEXT v.2.0.9,^42^ which avoids discarding data by using sample-size-based rarefaction and extrapolation of sampling curves to estimate sample completeness and alpha diversity metrics. Datatype was set to “abundance”, where abundance equals the number of reads assigned an ASV in a sample. To complement the 16S rRNA gene amplicon metrics for alpha diversity, we used Nonpareil 3.0^43^ to calculate the shotgun metagenome-based alpha diversity metric N_d_, which is based on the extent of overlap (redundancy) between trimmed and filtered reads of a metagenomic dataset. After checking for normality using Shapiro-Wilkes testing, we used nonparametric Wilcoxon tests to examine differences between symptomatic versus asymptomatic DEC infections and uninfected cases versus controls with a threshold of *p <* 0.05.

Between-sample beta diversity was compared for symptomatic versus asymptomatic DEC infections and for uninfected cases versus controls using Bray-Curtis dissimilarity matrices for 16S rRNA gene amplicon data and Mash^44^ k-mer-based distances of unassembled shotgun metagenome data. For 16S rRNA gene amplicon data, taxa that occurred fewer than two-times and in <10% of samples were removed prior to Bray-Curtis dissimilarity analyses and sample counts were rarefied to an even sequencing depth (1e4) to avoid library size biases.^45^ For the Mash analysis, reference sketches generated for all metagenomes using trimmed and filtered reads were used to generate an all-versus-all Mash distance matrix. Both Bray-Curtis and Mash distance matrices were visualized using NMDS ordinations. We tested for homogeneity of dispersion among groups (symptomatic versus asymptomatic DEC infections and uninfected cases versus controls) using the betadispersion function in the R package vegan,^46^ which calculates average distances of sample group distributions from the distribution centroids. We also performed permutational multivariate analyses of variance (PERMANOVA) testing with 1e4 permutations using the adonis function in vegan to test for significant differences in the centroids of distributions for symptomatic versus asymptomatic DEC infections and uninfected cases versus controls with a significance threshold of *p <* 0.05.

To understand how *E. coli* in the gut impacted diversity comparisons, we also ran alpha and beta diversity comparisons after filtering *E. coli* from our dataset. Bacterial ASVs annotated as *E. coli* were removed from 16S rRNA gene amplicon diversity analyses, and BMTagger was used to filter shotgun metagenome reads that mapped to an *E. coli* reference assembly (NC_000913.3).

#### Differentially abundant taxa

Relative abundances of 16S rRNA gene amplicon family-level taxa were compared between sample groups using two approaches: i) a beta-binomial regression model that accounts for within-sample taxa correlation and uneven sequencing depths,^47^ and ii) linear discriminant analysis (LDA) with effect size corrections (LEfSe).^48^ Symptomatic infections, asymptomatic infections, uninfected cases, and uninfected controls (four total sample groups) were compared using both methods with significance thresholds of adjusted *p<*0.05. The analysis was also conducted after filtering to include only samples from participants aged 5 and under. Beta-binomial regressions were run using the R package corncob^47^ and included corrections for participant age category (adult, child, toddler, or infant) on relative abundance and age category and sample group on variance. LEfSe was run using the R package microbiomeMarker^49^ and taxa with LDA values >3 were considered significantly differentially abundandant. Mean relative abundances of significant microbial families by sample group were plotted with default hierarchical clustering using ComplexHeatmap^41^ following square-root transformation to improve visualization of less abundant taxa.

The same statistical approaches (corncob and LEfSe) were used to test for differential relative abundances of family-level shotgun metagenome data by diarrhea and DEC infection status. However, the corncob models failed to converge due to insufficient sample size and variance between groups, so only LEfSe results are reported. Similarly, there was insufficient data to conduct the sub-analysis for participants aged less than 5 years due to insufficient sample size and variance by either the LEfSe or corncob statistical methods.

#### Functional potential

Open reading frames (ORFs) were annotated on shotgun metagenome contigs >2kbp using Prodigal^50^ with the metagenome flag (−p meta). Prodigal-annotated ORFs were clustered using Linclust^51^ at 90% nucleotide identity. The longest sequence from each cluster was chosen as the representative sequence and extracted using a custom Python script. Trimmed metagenome reads were aligned to dereplicated ORF sequences using blastn,^40^ and the sequencing depth and relative abundance of each gene in each metagenome was calculated as described above for virulence factor genes. Representative sequences for each ORF cluster were assigned Kyoto Encyclopedia of Genes and Genomes (KEGG) Orthology (KO) annotations using the automated MicrobeAnnotator pipeline.^52^ KO-assigned genes were grouped into KEGG pathways using KEGG API mapping files (https://www.kegg.jp/kegg/rest/keggapi.html). KEGG annotations were then filtered to remove those not associated with bacteria. Kruskal–Wallis rank sum testing with BH FDR p-value corrections was performed to test for differential abundance of KO-annotated genes by DEC infection and diarrhea status. KEGG-annotated gene functions that were significantly different between sample groups (BH adjusted *p* < 0.05) were binned into higher-level secondary KEGG hierarchical categories and the mean relative abundance of all KO-annotated genes within each secondary KEGG category and sample group was plotted using default hierarchical clustering in ComplexHeatmap.^41^

### Pathogen characteristics

#### DEC isolate virulence potential

Virulence factor genes from the curated VFDB^53^ were identified in the DEC isolate genomes using the same read-based methods described above for the shotgun metagenomes. Following normality testing using Shapiro-Wilkes tests, we used Wilcoxon rank sum tests with a significance threshold of *p<*0.05 to determine whether the number of virulence genes present in isolate genomes differed between symptomatic versus asymptomatic DEC infections and between uninfected cases versus uninfected controls.

#### DEC isolate relatedness and accessory gene content

ORFs were annotated on DEC isolate genome contigs >2kbp using Prokka v.1.14.6^54^ and annotated and aligned with Roary v.3.12.0.^55^ A phylogenetic tree was inferred from the Roary core genome alignment using FastTree v.2.1.10^56^ with the general time-reversible model. The reliability of tree topology was assessed using FastTree support values, which are based on the Shomodaira-Hasegawa test of three alternate topologies around each split. The tree was midpoint rooted and visualized using ggtree.^57^ Phylogenetic signal tests were performed by calculating a δ statistic, a phylogenetic analog of the Shannon entropy that measures degree of phylogenetic signal between categorical traits (symptomatic/asymptomatic DEC infection status) and a phylogeny.^58^ We applied 1000 iterations and set the p-value threshold at 0.05. We tested for accessory ORFs associated with isolates from symptomatic versus asymptomatic infections using Scoary v.1.6.16^59^. The Roary core genome phylogenetic tree was used to infer population structure for Scoary and we applied a significance threshold of BH-corrected *p* < 0.05.

## Results

### Pathogen abundance

#### Relative abundance of E. coli in the gut

**Figure 2.**
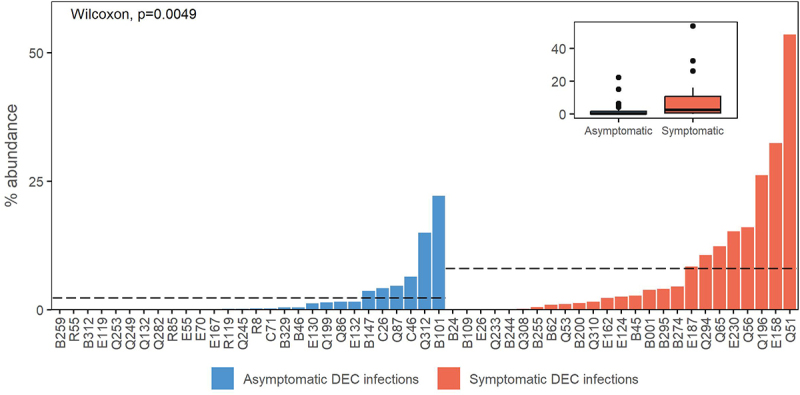
DEC isolate metagenome abundances. Dashed lines indicate mean abundance for case versus control sample groups. Data are also shown as an inset box plot.

Relative abundance of DEC isolate genome sequences in the corresponding shotgun metagenome was higher on-average in individuals with symptomatic DEC infections than those with asymptomatic DEC infections (*p=*0.0049; Figure 2). DEC isolate abundance in the metagenomes was highly correlated with the relative abundance of *E. coli* by qPCR (Supplementary Figure S1). We observed the same trend of higher DEC isolate relative abundance in symptomatic versus asymptomatic infections when the data were filtered to include only participants aged 5 and under (*p* = 0.02; Supplementary Figure S2).

#### Number and relative abundance of virulence genes in the gut

**Figure 3.**
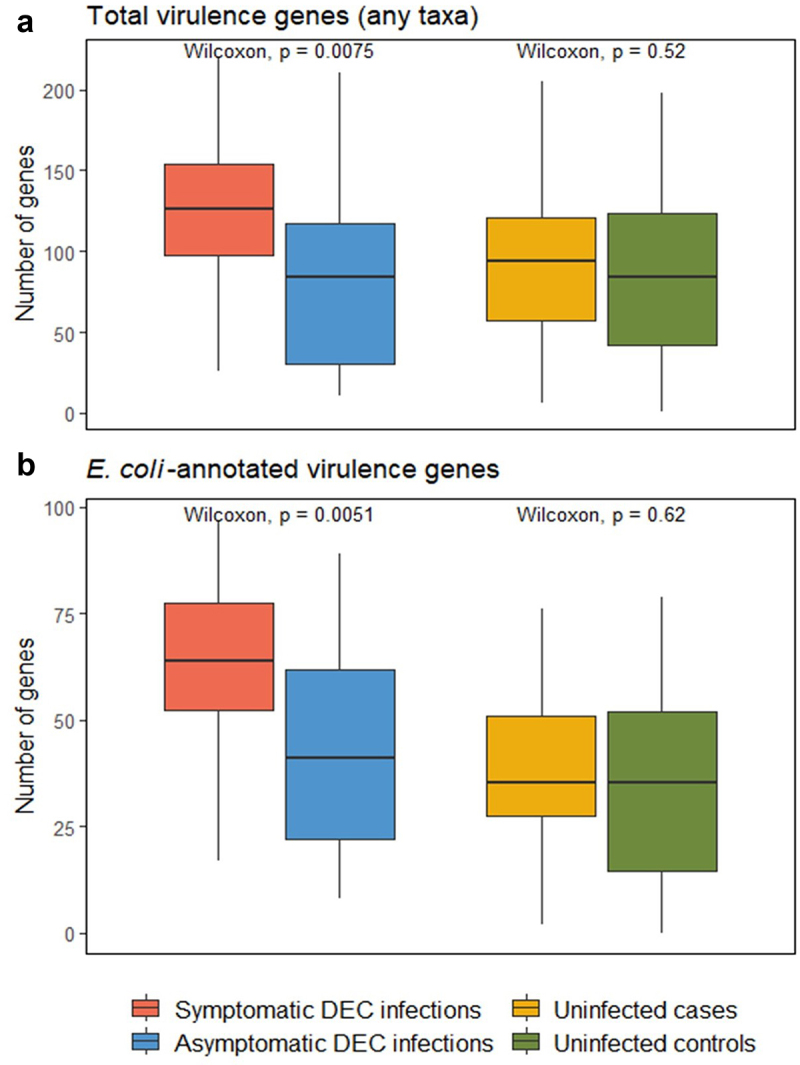
Comparison of the numbers of virulence genes from the virulence factor database (VFDB) in symptomatic versus asymptomatic DEC infections and in uninfected cases versus controls. Data are shown for virulence genes in the VFDB annotated as any taxa (a) and as *E. coli* (b).

There were significantly more total virulence genes detected in symptomatic compared to asymptomatic DEC infections (*p* = 0.0075; Figure 3a), with a mean of 127.96 virulence genes in symptomatic infections and 84.72 in asymptomatic infections. In contrast, there was no significant difference in the number of total virulence genes between uninfected cases versus controls. We found a similar trend when we looked at the relative abundance of *E. coli-*annotated virulence genes; there were more virulence genes in symptomatic than asymptomatic DEC infections (*p* = 0.0051; Figure 3b), but no significant difference in the number of virulence genes present in the gut microbiomes of uninfected cases versus controls. The same trends were observed for the sub-analysis of participants aged 5 and under, where the numbers of virulence genes were significantly different between symptomatic and asymptomatic DEC infections (*p* = 0.025 and *p=*0.021 for total and *E. coli-*annotated genes, respectively) but there were no differences between uninfected case versus control samples (Supplementary Figure S3). There were no *Salmonella* or *Campylobacter*-annotated virulence genes that passed our filtering criteria of presence in >10% of metagenomes, and the number of *Klebsiella*-annotated virulence genes was not significantly different by infection or diarrhea status for either the all-sample or under-age-5 analyses.

**Figure 4.**
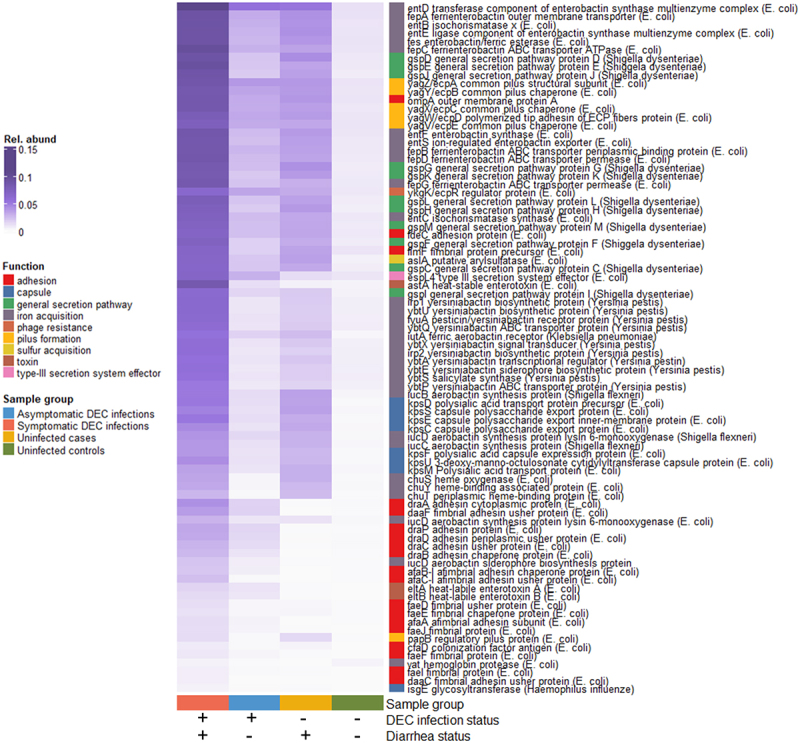
Mean relative abundances of virulence genes that were significantly differentially abundant by DEC infection and diarrhea case/control status (4-way Kruskal-Wallis adjusted *p-*values < 0.05).

The analysis of virulence factor relative abundances revealed 90 virulence genes from 23 bacterial operons that were differentially abundant by DEC infection and diarrhea status, when considering all samples (Figure 4). Of these, 63 were annotated as *E. coli* and 16 were annotated as closely related *Shigella* spp. There were also 10 genes annotated as *Yersinia pestis* and one annotated as *Klebsiella pneumonia*. These genes were all associated with siderophore production and transport and had similar relative abundances across sample groups. Overall, relative abundances of significant genes (BH adjusted *p* < 0.05) were highest in symptomatic DEC infections, followed by uninfected individuals with diarrhea, then asymptomatic infections. Samples from uninfected controls had very low virulence gene abundances. Gene functions that were enriched in symptomatic infections included pilus formation (*yag*/*ecp* and DAEC-associated *afa*, *dra*, and *daa* operon genes) and secretion pathways (*gsp* operon genes). Several differentially abundant capsule (*kps* operon genes) and hemoglobin binding (*chu* operon genes) had approximately the same abundances in symptomatic infections and infected cases. Results were similar when the data were filtered to include only participants under age 5 (Supplementary Figure S4).

### Gut microbiome characteristics

#### Alpha diversity

**Figure 5.**
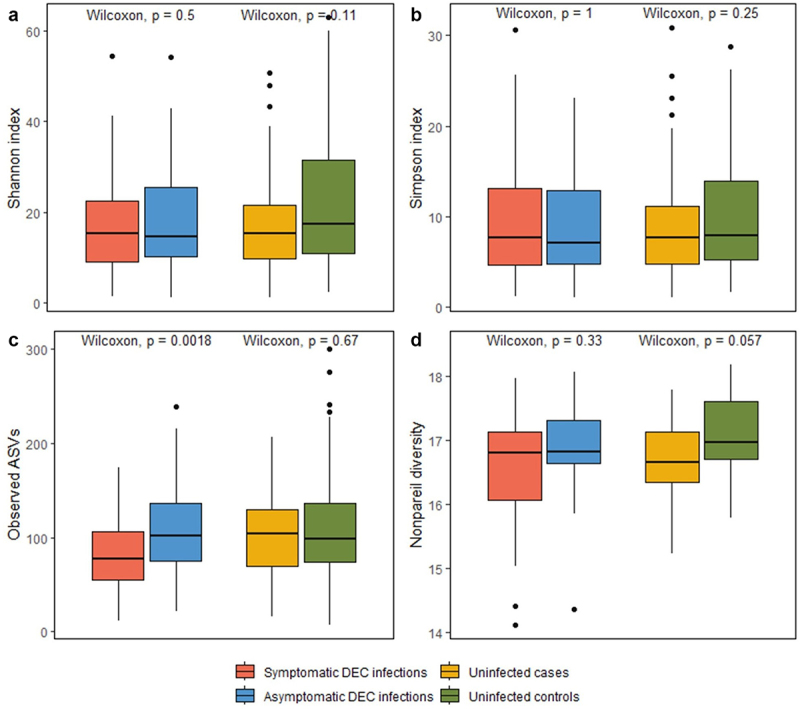
Comparisons of coverage-based estimates of within-sample alpha diversity for symptomatic versus asymptomatic DEC infections and for uninfected cases versus controls. Shannon, Simpson, and observed alpha diversity were calculated using 16S rRNA gene amplicon data (a-c); Nonpareil alpha diversity was calculated using shotgun metagenome data (d).

Alpha diversity metrics calculated for both 16S rRNA gene amplicon data (Shannon, Simpson, Observed ASVs) and shotgun metagenomes (Nonpareil) had slightly higher values for asymptomatic DEC infections than symptomatic DEC infections, and for uninfected controls than cases (Figure 5). However, this relationship was only significant for the Observed ASV comparison of asymptomatic DEC infections and symptomatic DEC infections; asymptomatic DEC infections had significantly higher observed ASVs (species richness) than symptomatic DEC infections (*p* = 0.0018). Similar results were observed for alpha diversity comparisons when *E. coli* was filtered from the analysis (Supplementary Figure S5). Alpha diversity metrics are summarized in Supplementary Table 9.

#### Beta diversity

NMDS visualizations of Bray-Curtis dissimilarity matrices (16S rRNA gene amplicon data) and Mash distances (shotgun metagenome data) showed substantial overlap in 2D space for symptomatic versus asymptomatic DEC infections or uninfected cases versus controls (Supplementary Figure S6). PERMANOVA testing indicated that there was a significant difference in the centroids of distribution for Bray-Curtis dissimilarities for uninfected cases versus controls (*p* = 0.016; Supplementary Figure S6b). However, case/control status explained only 1.00% of variation (R^2^ = 0.10) for that comparison. All other beta diversity comparisons were nonsignificant. Results were similar when we re-ran the analysis with *E. coli* reads/ASVs removed (Supplementary Figure S7), and beta diversity statistics are summarized in Supplementary Table S10.

#### Differentially abundant taxa

**Figure 6.**
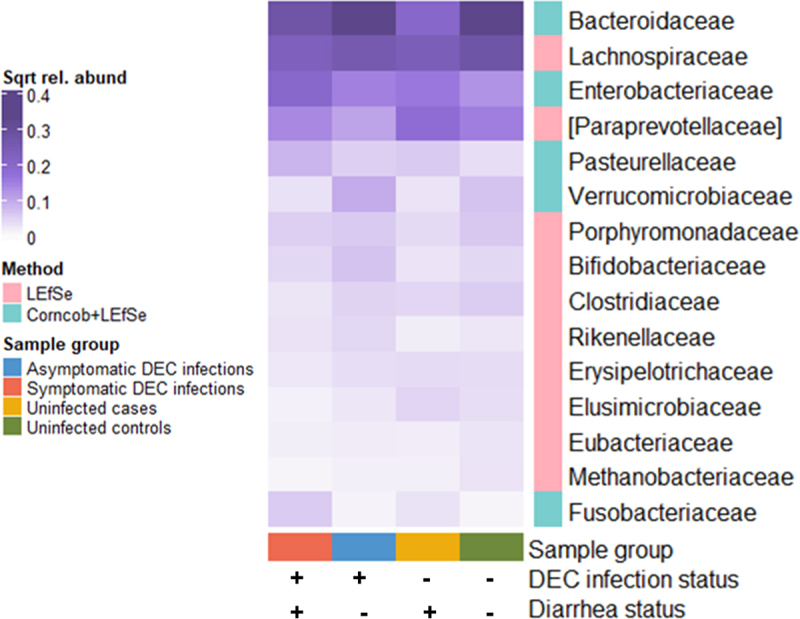
Mean relative abundances of 16S rRNA gene amplicon family-level bacterial taxa that were significantly associated with diarrhea and DEC infection status (corncob and/or LEfSe analyses; adjusted *p <* 0.05, LEfSe LDA threshold < 3). Values are square root transformed to improve visualization of taxa with low relative abundances.

Statistical analyses of 16S rRNA gene amplicon taxonomic abundances revealed 15 family-level bacterial taxa that were differentially abundant by DEC infection and diarrhea status (Figure 6; data are also shown as boxplots in Supplementary Figure S8). The families *Enterobacteriaceae*, *Pasterurellaceae*, and *Fusobacteriaceae* were enriched in DEC infections. The commensal families *Bacteroidaceae* and *Lachnospiraceae* had highest abundances in non-diarrheal samples, and the *Bifidobacteriaceae*, *Rikenellaceae*, and *Verrucomicrobiaceae* were enriched in asymptomatic DEC infections. The *Paraprevotellaceae* were more abundant in uninfected cases and controls than in samples from individuals with DEC infections. In the sub-analysis for participants under age 5, only the *Enterobacteriaceae*, *Paraprevotellaceae*, *Pasteurellaceae*, and *Clostridiaceae* were significantly differentially abundant between sample groups (Supplementary Figures S9 and S10). These taxa followed the same patterns of differential abundance between sample groups in the under-age-5 analysis as they did for all samples, though the increase in *Paraprevotellaceae* relative abundance in uninfected controls compared to other sample groups was more striking in participants under age 5.

There were four family-level taxa that were differentially abundant by diarrhea and DEC infection status in the analysis of the shotgun metagenome data: the *Enterobacteriaceae*, *Streptococcaceae*, *Erysipelotrichaceae*, and *Bacteroidaceae* (Supplementary Figures 11 and 12). Except for the *Streptococcaceae*, these taxa were also differentially abundant in the 16S rRNA gene amplicon analysis. The patterns of abundance for taxa that were found in both the 16S rRNA gene amplicon and shotgun metagenome analyses were similar by DEC infection and diarrhea status. In particular, the *Bacteroidaceae* were enriched in uninfected controls and the *Enterobacteriaceae* were enriched in symptomatic infections in both the 16S rRNA gene amplicon and shotgun metagenome analyses.

#### Functional potential

**Figure 7.**
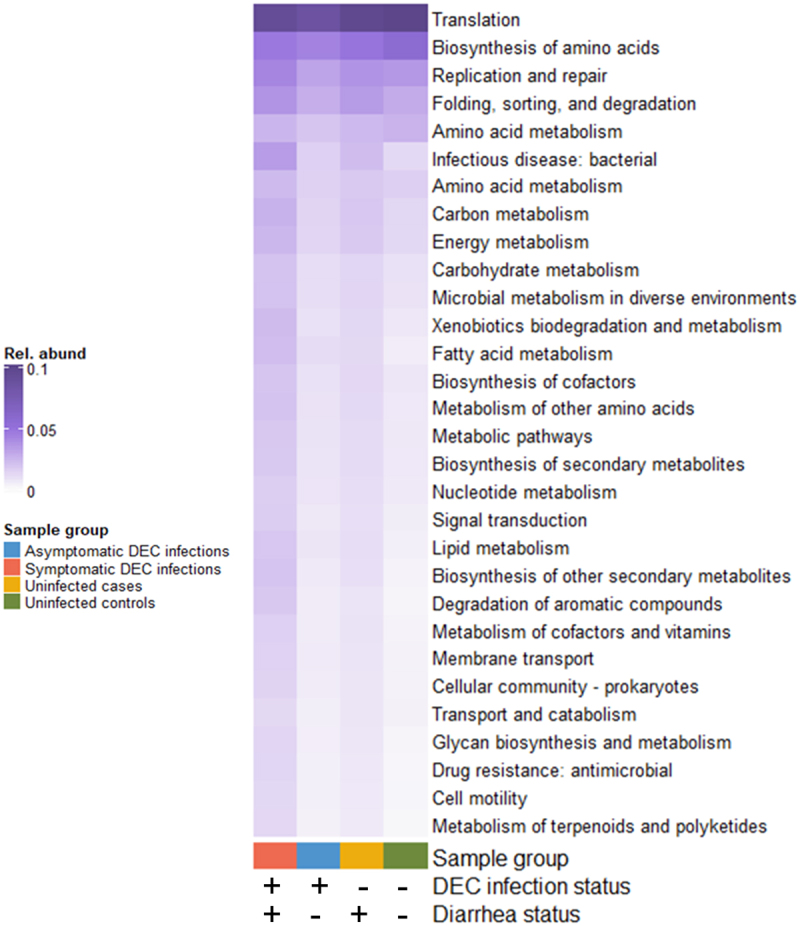
Mean relative abundances of genes with secondary KEGG annotations that were significantly differentially abundant by DEC infection and diarrhea status (4-way Kruskal-Wallis adjusted *p*-values < 0.05).

Of 1,256,905 dereplicated ORFs from the shotgun metagenomes, 464,181 (37%) were assigned KO numbers associated with 6,221 unique gene functions. Statistical testing resulted in 1,338 KO-annotated gene functions with differential relative abundances between sample groups by DEC infection and diarrhoea status with a B-H adjusted *p*-value < 0.05. KEGG-annotated secondary gene functions had the highest mean relative abundance in symptomatic DEC infections, followed, in order, by uninfected diarrheal cases, asymptomatic infections, and uninfected non-diarrheal controls (Figure 7).

### Pathogen characteristics

#### Number of virulence genes

There were no significant differences in the number of total or *E. coli-*annotated virulence genes annotated in the genomes of DEC pathotype isolates from symptomatic versus asymptomatic infections at *p* > 0.05.

#### Strain relatedness and trait-associated genes

**Figure 8.**
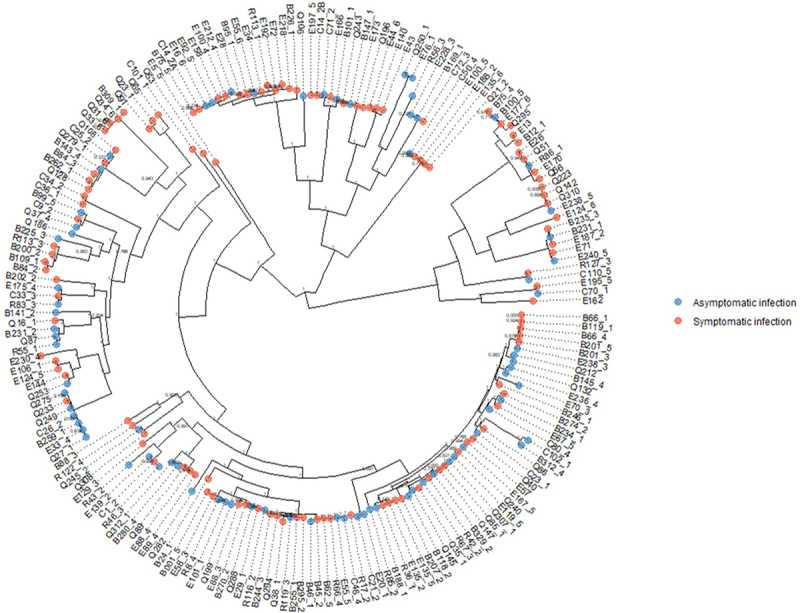
Core genome phylogenetic tree of DEC strains from symptomatic and asymptomatic individuals; there was no phylogenetic signal for case/control status (δ = 0.59, *p =* 0.92).

The core-genome phylogenetic tree (Figure 8) was mixed, with no clear structure or phylogenetic signal based on isolation from a symptomatic versus asymptomatic DEC infection (*p* = 0.92). We did, however, observe small, tight clusters of closely related strains that were all or majority from symptomatic or asymptomatic DEC infections. There were no accessory genes in DEC isolate genomes that were significantly associated with either symptomatic or asymptomatic sample groups with a corrected p-value cutoff of 0.05.

## Discussion

We utilized epidemiological data from a case/control study of diarrhea in combination with sequencing data from DEC pathotype isolates and corresponding gut microbiome data to improve understanding of why some DEC infections result in diarrhea while others do not. This integration of an epidemiological study design with multiple data types from the same samples enabled us to go beyond simple pathogen detection to examine how i) pathogen abundance in the gut microbiome, ii) gut microbiome community characteristics, and iii) pathogen genome characteristics contribute to symptomatic versus asymptomatic outcomes for DEC infections. The results of the analyses we conducted are summarized in Table 1.

**Table 1.** Results summary for analyses comparing pathogen abundance, gut microbiome characteristics, and pathogen characteristics for symptomatic versus asymptomatic DEC infections.

Factor	Figure	Analysis description	Primary result	Supporting result(s)
Pathogen abundance	Figure 2. *E. coli* relative abundance in the gut	Compared the relative abundance of DEC isolate sequences in the corresponding shotgun metagenome to determine the amount of *E. coli* in the gut for symptomatic versus asymptomatic DEC infections.	*E. coli* relative abundance was highly variable but higher on-average (*p=*0.0049) in symptomatic compared to asymptomatic DEC infections.	There was a strong correlation between the metagenome and qPCR-based *E. coli* abundance estimates (Figure S1). Results were similar for a sub-analysis of participants aged <5 years.
	Figure 3. Number of virulence genes in the gut	Compared the number of total and *E. coli*-annotated bacterial virulence genes in the gut.	There were more total (*p=*0.0075) and *E. coli-*annotated (*p=*0.0051) virulence genes in symptomatic than asymptomatic DEC infections.	There was no significant difference in the number of virulence genes in uninfected cases versus controls. There were no *Salmonella* or *Campylobacter*-annotated virulence genes that passed our filtering criteria of presence in > 10% of metagenomes, and the number of *Klebsiella*-annotated virulence genes was not significantly different by infection or diarrhea status. Results were similar for a sub-analysis of participants aged <5 years.
	Figure 4. Relative abundance of differentially abundant virulence genes in the gut	Compared the mean relative abundance of bacterial virulence genes that were significantly differentially abundant between sample groups.	Virulence genes that were significantly different (adjusted *p <* 0.05) between groups were most abundant in symptomatic DEC infections.	Abundances were next-highest in uninfected cases, followed by asymptomatic DEC infections. Those without diarrhea or a DEC infection (uninfected controls) had very low virulence factor abundances. Most genes were annotated as *E. coli* or closely related *Shigella* spp. Gene functions included pilus formation, nutrient acquisition, secretion systems, adhesion, and other classical virulence functions. Results were similar for a sub-analysis of participants aged <5 years.
(ii) Gut microbiome characteristics	Figure 5. Gut microbiome alpha diversity	Compared within-sample alpha diversity metrics calculated using 16S rRNA gene amplicon (Shannon, Simpson, and Observed ASVs) and shotgun metagenome (Nonpareil) gut microbiome data.	Alpha diversity was higher in asymptomatic than symptomatic DEC infections, though the comparison was only significant (*p<*0.05) for Observed ASVs.	A similar trend was observed for uninfected cases versus controls, where individuals without diarrhea had higher alpha diversity. Results were similar after filtering *E. coli* from the analyses.
	Figure 6. Differential abundance of gut microbiome taxa	Tested for differentially abundant family-level taxa in the gut microbiome using 16S rRNA gene amplicon data.	Bacterial families belonging to the *Proteobacteria*, including the *Enterobacteriaceae* and *Pasteurellaceae* were most abundant in symptomatic DEC infections, while asymptomatic infections were associated with high relative abundances of short chain fatty acid producers and strict anaerobes, including the *Bifidobacteriace*, *Bacteroidaceae*, and *Verrucomicrobiaceae.*	There were also differences in the relative abundance of gut microbiome taxa associated with case/control status, even in uninfected individuals; for example, individuals without DEC infections had high relative abundance of *Paraprevotellaceae*, regardless of diarrhea symptoms. Results were similar, though with fewer significant taxa, with differential relative abundance for the 16S rRNA gene amplicon sub-analysis of participants aged <5 years and the shotgun metagenome analysis.
	Figure 7. Relative abundance of genes associated with high-level cellular functions	Compared the mean relative abundances of bacterial functional genes that were significantly differentially abundant by DEC infection and diarrhea status.	Functional genes that were significantly different (*p<*0.05) between groups were most abundant in symptomatic DEC infections and spanned a wide array of high-level molecular functions.	Gene functions that were enriched in symptomatic DEC infections included those important for bacterial infection (capsule, pilus formation, etc.), cell maintenance, and the biosynthesis and metabolism of macromolecules.
(iii) Pathogen characteristics	Figure 8. Core genome phylogenetic tree	Tested for trait-associated phylogenetic signal using a core genome phylogeny of DEC isolates from symptomatic versus asymptomatic infections.	The core genome DEC isolate tree was mixed, with no phylogenetic signal based on isolation from an symptomatic versus asymptomatic infection.	Despite the lack of overall structure in the tree, we did observe small, tight clusters of closely related DEC strains that were all or majority from symptomatic or asymptomatic infections. There were no isolate virulence or accessory gene sequences associated with isolation from symptomatic versus asymptomatic infections.

Pathogen abundance, as determined by DEC isolate relative abundance and the presence and relative abundances of virulence factors in the shotgun metagenome gut microbiomes, played a strong role in determining symptomatic versus asymptomatic DEC infections. Compared to asymptomatic DEC infections, symptomatic DEC infections had higher relative abundance of DEC isolate sequences in the gut, higher numbers of total and *E. coli*-annotated virulence genes, and higher abundance of differentially abundant virulence genes. The microbial communities in the gut microbiome also showed some signals associated with symptoms of DEC carriage, with a trend toward higher taxonomic richness in asymptomatic infections, differential abundance of several taxa in symptomatic versus asymptomatic DEC infections, and greater abundance of differentially abundant functional genes in symptomatic infections. We saw that samples from diarrhea cases exhibited differential patterns from non-diarrheal controls, even in uninfected individuals, as demonstrated by higher virulence and functional gene relative abundances in cases compared to controls. Across all analyses, uninfected controls provided a comparison group, and had the lowest number and relative abundance of differentially abundant virulence factors and functional genes.

### Pathogen abundance

We used a read-based approach to measure the abundance of DEC isolate genome sequences in metagenomic gut microbiome data from the sample and found an on-average higher abundance of *E. coli* in the guts of individuals with symptomatic DEC infections. Higher pathogen loads in individuals with symptomatic versus asymptomatic infections have been previously observed for DEC and other enteropathogens, and are hypothesized to be an indicator of the extent to which pathogen colonization of the gastrointestinal tract has occurred.^20,22^ The presence of DEC or other pathogens in the stool of a healthy individual without diarrhea may reflect the recent ingestion of an inoculum that is insufficient to colonize the GI tract or cause diarrhea, prolonged asymptomatic shedding following diarrheal illness, or an infection that has not yet resulted diarrhea symptoms but will in the future.^6^ Similarly, patients who present with diarrhea but no or low abundance of pathogens may have diarrhea caused by a coinfecting and undiagnosed causative agent or noninfectious secretory diarrhea.^6^ Though measurements of pathogen abundance are an improvement from simple detection and have been associated with diarrhea symptoms in this and other studies,^14,20,22^ we detected a high degree of variation in DEC isolate metagenome abundance within both the symptomatic and asymptomatic sample groups. Given this variability, pathogen abundance alone may not be a sufficient indicator of association with diarrhea status for DEC infections.

The number of virulence genes in the gut was higher in individuals with symptomatic versus asymptomatic DEC infections, but there was not a case/control difference for uninfected individuals. These results demonstrate that, at least for DEC infections, diarrhea symptoms are associated with a more virulent gut microbiome state. This was similarly evident when we looked at differentially abundant virulence genes; individuals with symptomatic DEC infections had higher abundances of significantly differential virulence genes than those with asymptomatic DEC infections. Most differentially abundant virulence genes were annotated as *E. coli* or closely related *Shigella* species. We screened for and did not detect any Shigellae in our study, so we think it is likely that *Shigella-*annotated virulence genes are associated with *E. coli*.

We also detected several iron acquisition genes that were annotated as *Yersinia pestis* and one that was annotated as *Klebsiella pneumoniae* that had similar relative abundances across sample groups. Like *E. coli*, *Klebsiella pneumoniae* is a common member of the gut flora that can acquire virulence genes and cause diarrheal disease^60^. *Yersinia pestis*, on the other hand, is not typically associated with gut microbiome communities. The differentially abundant *Yersinia pestis*-annotated genes that we identified are associated with the production and transport of the siderophore yersiniabactin. While these genes and the mobile genetic elements that encode them were first discovered in *Yersinia pestis*, they have also been found on horizontally transferred pathogenicity islands in virulent strains of both *E. coli* and *Klebsiella pneumoniae*. ^e.^^g., 61–63^ Given the high relative abundances of these genes in diarrhea cases where we confirmed a DEC infection and their previously reported identification in *E. coli* and *Klebsiella pneumoniae*, this result does not necessarily indicate the presence of *Yersinia pestis* in the gut.

In addition to genes associated with iron scavenging, differentially abundant relative virulence gene functions included genes associated with adhesion, capsule formation, and toxins. All of these are classical virulence gene functions, but several, including those for pilus formation, capsule/biofilm formation, and iron scavenging are also induced by the commensal gut microbiota to initiate colonization or outcompete bacterial pathogens.^64–66^ On the other hand, many of the toxins and adhesins have been clearly associated with DEC infections. These were unsurprisingly most abundant in symptomatic DEC infections and were absent in uninfected individuals. They include members of the *dra*, *afa*, and *daa* gene operons, which code for DAEC adhesins that have been implicated in pathogenesis and are commonly used to detect DAEC carriage,^10^ and *eltA* and *eltB* enterotoxin genes, which are diagnostic for ETEC and have been associated with diarrhea.^14^

Interestingly, the second-highest abundances of many differentially abundant virulence genes were found in uninfected cases, where diarrhea was present, but we did not detect DEC by isolation. We looked for *Campylobacter*, *Klebsiella*, and *Salmonella* virulence genes in these samples to see if there was evidence that these diarrhea cases where we did not detect DEC were caused by other common bacterial enteropathogens in the region, but virulence gene counts and relative abundances associated with these taxa were not enriched in uninfected cases. Since we did detect *E. coli* and closely related *Shigella-*annotated virulence genes in this sample group, it is possible (and perhaps likely) that we missed detection of DEC by isolation, or that diarrhea symptoms were caused by a virus, parasite, or other enteropathogenic bacteria that we did not screen for. Molecular methods that quantify virulence genes are increasingly being used to detect DEC in stool samples, and are likely to be more sensitive than the culture-dependent approach used here.

### Gut microbiome characteristics

The gut microbiome impacts the ability of diarrheal pathogens to colonize the gastrointestinal tract by acting as a physical barrier to enteropathogen attachment and producing inhibitory substances.^6,67^ We identified variations in gut microbiome richness, taxonomic abundances, and functional potential associated with symptomatic and asymptomatic DEC infections and uninfected cases and controls. For within-sample alpha diversity, there was a trend of higher diversity in non-diarrheal samples for both DEC infected and uninfected sample groups. These results are consistent with previous research that has demonstrated lower within-sample gut microbiome richness and/or diversity associated with diarrhea.^68^ We did not see any large differences in between-sample beta diversity by diarrhea or DEC infection status.

We tested for differential taxonomic abundances of family-level gut taxa using both the 16S rRNA gene amplicon and shotgun metagenome data. Results were similar between the two methods, though there were fewer significantly differentially abundant taxa in the shotgun metagenome analyses. We have focused our discussion on the 16S rRNA gene amplicon-based analysis because of the larger sample size in the 16S rRNA gene amplicon dataset. When considering all samples, symptomatic DEC infections were associated with an increase in the relative abundance of the bacterial taxa *Enterobacteriaceae*, *Fusobacteriaceae*, and *Pasteurellaceae*. These three bacterial families have been described as “pathobionts” in gut microbiome communities, and the *Enterobacteriaceae* in particular, which were also enriched in symptomatic DEC infections in the sub-analysis of children under age 5, are frequently linked to gut microbiome dysbioses^69,70^. While not necessarily pathogenic themselves, increased abundances of pathobionts have been associated with enteropathogen infection, diarrhea, and chronic inflammatory bowel disorders^71–73^. Though they may be benign in a healthy gut state, pathobiontic *Enterobacteriaceae* proliferate during host inflammatory immune responses triggered by pathogen invasion or other disturbances^69^. Mouse models have provided possible mechanisms for this phenomenon. For example, *E. coli* and other members of the *Enterobacteriaceae* can use host-derived nitrate produced by inflammatory immune processes as an alternate electron receptor, conferring a competitive advantage over other commensals^74^. Colonization with *E. coli* and other *Enterobacteriaceae* in mice results in prolonged carriage of enteropathogens and gut abnormalities^75^. High levels of genetic variability, frequent participation in horizontal gene transfer, and the ability to proliferate in the presence of oxygen are also thought to give the *Enterobacteriaceae* selective advantage during gut microbiome disruptions^76,77^. The expansion of *Enterobacteriaceae* and other pathobionts thus results in a positive feedback loop in individuals carrying DEC or other enteropathogens where the proliferation of pathobiontic taxa results in inflammation and reduced resistance to pathogen colonization which then facilitates further inflammation and pathogen and pathobiont proliferation.^76^

The families *Bacteroidaceae* and *Lachnospiraceae* were abundant in non-diarrheal control samples, regardless of DEC infection status. Both are anaerobic and produce short-chain fatty acids (SCFAs) and other metabolic byproducts that are beneficial for the host and intestinal microbiota, although there are different outcomes depending on the specific taxa present.^78,79^ In particular, *Bacteroidaceae* and related lineages are protective for diarrhea in volunteer and epidemiological studies.^80,81^ The *Paraprevotellaceae* were enriched in the gut microbiomes of individuals that were not infected with DEC. Studies of children in LMIC settings have found that members of the *Paraprevotellaceae* are associated with reduced pathogen burden and lack of gastrointestinal disorder,^82,83^ but members of this family have also been associated with disease states.^84,85^ The *Verrucomicrobiaceae*, *Rikenellaceae*, and *Bifidobacteriaceae* were most abundant in individuals with asymptomatic DEC infections, suggesting that these taxa may confer some advantage in neutralizing DEC pathogenicity and the development of diarrhea symptoms. The *Bifidobacteriaceae* are considered to be contributors to healthy microbiomes, particularly in early life as several *Bifidobacteria* species are important for human milk metabolism and pathogen inhibition.^86,87^

We conducted a broad analysis of functional genes in the gut microbiome and found that genes that were differentially abundant were most abundant in symptomatic DEC infections. This followed the pattern we observed in the virulence factor analysis, where the largest differences in gene abundance were between diarrheal cases and non-diarrheal controls, regardless of DEC infection status. These significantly differentially abundant genes span a variety of high-level molecular functions, some of which are relevant to DEC pathogenesis and are similar to the gene functions we observed in the virulence factor analyses. These included genes important for bacterial infection (toxins and adhesins), cell motility (pilus genes), and prokaryotic cellular communities (capsule genes). Other gene functions however, including those associated with cell maintenance and the biosynthesis and metabolism of macromolecules, are less obviously linked to dysbiotic microbiome states. The higher relative abundances of these housekeeping and metabolic genes in DEC-positive diarrheal individuals may be a functional reflection of the increased proliferation of pathobionts in symptomatic DEC infections. Additional fine-scale and targeted metagenomic analyses, and potentially deeper sequencing and/or additional datatypes (*i.e*., transcriptomic, metabolomic data), are needed to fully characterize the links between these functional differences, the taxonomic composition of the gut microbiome, and diarrhea symptoms of enteropathogen infections.

### Pathogen characteristics

We used whole-genome sequencing data for DEC isolates to investigate whether DEC strains isolated from symptomatic infections had phylogenetic signatures or functional potentials that differentiated them from isolates from asymptomatic infections. Our results for all strain-level analyses were null. For the phylogenetic tree, however, we did observe subclades of closely related (nearly clonal) isolates where all strains were from either cases or controls. This near clonality in the tree structure could suggest that we have sampled several small-scale outbreaks caused by distinct strains or collections of very closely related strains. In contrast to our null results, volunteer challenge studies have demonstrated strain-level variation in diarrhea symptoms and severity for some enteropathogens, including early studies of DEC pathotypes.^88,89^ However, these studies compared very few strains, whereas the diversity of strains and DEC pathotypes in our study may be more reflective of real-world population-level host and pathogen dynamics.

## Limitations

This study had limitations. First, sample collection was cross-sectional and enteropathogen detection was focused primarily on DEC infections. Longitudinal studies that combine microbiome data with multiplex detection of multiple viral, parasitic, and bacterial enteropathogens are needed to refine understanding of how gut microbiome conditions impact initiation and recovery from a diarrheal episode, and how the presence and abundance of DEC versus other or coinfecting enteropathogens contribute to diarrhea symptoms. Second, we designed this study to encompass pathogen and gut microbiome differences for any DEC pathotype. This approach allowed us to focus on broad differences associated with DEC infections but did not capture important between-pathotype differences, though pathotype-associated gut microbiome differences have been previously explored for this dataset^19,90^. Lastly, there are factors associated with symptomatic versus asymptomatic carriage of DEC or other enteropathogens that we were not able to explore here. In particular, host-associated factors such as nutrition and immune status are likely important for determining host responses to enteropathogen colonization and the development of diarrhea symptoms.

## Conclusions

Asymptomatic carriage of DEC influences our understanding of the roles of these pathogens as causative agents of infectious, acute diarrhea. The findings reported here add support for the idea that pathogen abundance and gut microbiome differences are associated with symptomatic versus asymptomatic outcomes for DEC infections in a resource-limited and high enteropathogen transmission setting. DEC pathogen colonization and diarrhea represent related but distinguishable dysbioses for the gut microbiome, which are evidenced by differences in the taxonomic and functional potential of gut microbial communities when comparing gut characteristics associated with DEC infection versus diarrhea symptoms. Gut microbiome sequencing has potential to complement existing and established enteropathogen detection methods to understand diarrhea symptoms. This information can be used to help develop improved detection and diagnostic methods, and ultimately guide treatment approaches for DEC and other enteric infections. There is a clear need for longitudinal studies investigating the progression and resolution of DEC and other enteropathogen infections to fully understand how pathogen burdens and gut microbiome factors influence diarrhea symptoms. This research lays the groundwork for future studies investigating pathogen and microbiome characteristics associated with diarrhea and carriage of DEC and other enteropathogens.

## Supplementary Material

Gut Microbes supplement 2nd resubmission clean.docx

## Data Availability

The data that support the findings of this study are openly available under NCBI BioProject PRJNA486009. R code is available on Github: https://github.com/kjojess/EcoZUR-symptomatic-asymptomatic-manuscript.
