# Supplementary material for: Why are so many enteric pathogen infections asymptomatic? Pathogen and gut microbiome characteristics associated with diarrhea symptoms and carriage of diarrheagenic E. coli in northern Ecuador
Source: Gut Microbes. 2023 Nov 22;15(2):2281010. doi: 10.1080/19490976.2023.2281010 (PMC10730187; doi:10.1080/19490976.2023.2281010)
Supplement: Gut Microbes supplement 2nd resubmission clean.docx [file KGMI_A_2281010_SM4493.docx]

**Supplementary Material**

**Why are so many enteric pathogen infections asymptomatic? Pathogen and gut microbiome characteristics associated with diarrhea symptoms and carriage of diarrheagenic *E. coli* in northern Ecuador**

Kelsey J Jesser^a^, Gabriel Trueba^b^, Konstantinos T. Konstantinidis^c^, Karen Levy^a*^

*^a^Department of Environmental and Occupational Health Sciences, University of Washington, Seattle, WA, United States; ^b^Instituto de Microbiología, Universidad San Francisco de Quito, Ecuador; ^c^School of Civil and Environmental Engineering and School of Biological Sciences; Georgia Institute of Technology, Atlanta, GA, United States*

Corresponding author
Karen Levy: [klevy@uw.edu](mailto:klevy@uw.edu), (p) 206.543.4341

University of Washington School of Public Health, Hans Rosling Center for Population Health, Box 351618, 3980 15^th^ Avenue NE, Seattle, WA 98195.

**Table of Contents**

Detailed methods

Isolation and identification of DEC in whole stool 4

DNA extraction and sequencing 4

Whole genome scan for DEC diagnostic virulence genes 5

qPCR analyses 6

Figures

Supplementary Figure 1. DEC isolate metagenome and qPCR *E. coli* abundance 8

Supplementary Figure 2. DEC isolate metagenome abundance for ages <5 years 9

Supplementary Figure 3. Number of virulence genes for ages <5 years 10

Supplementary Figure 4. Abundance of virulence genes for ages <5 years 11

Supplementary Figure 5. Alpha diversity with *E. coli* removed 12

Supplementary Figure 6. Beta diversity 13

Supplementary Figure 7. Beta diversity with *E. coli* removed 14

Supplementary Figure 8. Boxplot of differential 16S rRNA gene amplicon taxa 15

Supplementary Figure 9. Heatmap of differential taxa for ages <5 years 16

Supplementary Figure 10. Boxplot of differential taxa for ages <5 years 17

Supplementary Figure 11. Heatmap of differential taxa for shotgun metagenomes 18

Supplementary Figure 12. Boxplot of differential shotgun metagenome taxa 19

Tables

Supplementary Table 1. DEC virulence gene endpoint PCR assays 20

Supplementary Table 2. Exclusion/inclusion criteria by sample type 21

Supplementary Table 3. Supplemental metadata 22

Supplementary Table 4. Shotgun metagenome sequencing metrics 33

Supplementary Table 5. Isolate sequencing metrics 36

Supplementary Table 6. 16S rRNA gene amplicon sequencing metrics 40

Supplementary Table 7. DEC pathotype gene reference sequences 44

Supplementary Table 8. *E. coli* and total bacteria qPCR assays 45

Supplementary Table 9. Alpha diversity statistics 46

Supplementary Table 10. Beta diversity statistics 47

References 48

**Detailed methods**

***Isolation and identification of DEC in whole stool***

Fresh stools from study participants were streaked onto MacConkey’s agar media. Up to five lactose-fermenting colonies and one non-lactose-fermenting colony (some *E. coli* are lactose non-fermenters) from each participant were streaked onto Chromocult agar (Merck, Darmsladt, Germany) to test for β-glucoronidase activity. Non-lactose-fermenting colonized were tested to differentiate Shigellae and *E. coli* using the API 20E test (BioMérieux, Marcy l’Etoile, France); no Shigellae were detected in the study. The five *E. coli* colonies isolated from each sample were pooled, resuspended, and boiled for 10 min in sterile water. Previously published conventional PCR assays (Supplementary Table 1) were used to screen pooled isolate lysates for DEC virulence genes, using the following virulence gene profiles to define DEC pathotypes: *aggR+* (EAEC)^1^, *afa+* (DAEC)^2^, *eaeA+* and *bfp+* (tEPEC)^28,29^, *eaeA+* and *bfp-* (aEPEC)^3,4^; *lt+* and/or *sta+* (ETEC), *ipaH+* (EIEC)^3^, and e*aeA*+, *stx1+* and/or *stx2+* (EHEC)^4^. If a pooled sample was positive for any DEC virulence gene target, then each of the five isolates from the pooled sample were retested individually to identify the specific isolate (or isolates) carrying that gene.

***DNA extraction and sequencing***

Presumptive DEC isolates were extracted using the Genomic DNA Purification Kit (Promega, Madison, WI) according to the manufacturer’s protocol. DNA from 0.2 mL homogenized whole stool was extracted using the MoBio (now Qiagen, Hilden, Germany) Powersoil DNA isolation kit according to the manufacturer’s protocol. A NanoDrop spectrophotometer (Thermo Scientific, Waltham, MA) and Qubit 2.0 dsDNA high sensitivity fluorometric assays (Invitrogen, Carlsbad, CA) were used to estimate DNA purity and yield for all extracts.

Isolate and shotgun metagenome libraries were prepared using the Illumina Nextera XT DNA library preparation kit with Illumina Nextera CD indexes according to the manufacturer’s instructions, with the modification that the protocol was terminated after isolation of cleaned, double-stranded libraries. Libraries were quantified using the Qubit 2.0 dsDNA high-sensitivity assay, and library insert sizes were confirmed using high-sensitivity DNA chip on a Bioanalyzer 2100 (Agilent).

For isolate libraries, an equimolar mixture of libraries (10 pM) was sequenced on an Illumina MiSeq instrument (Illumina, Inc., San Diego, CA) with a 2x250-bp paired-end run. Equimolar mixtures of shotgun metagenome libraries were sequenced using an Illumina HiSeq 2500 using the rapid run mode for 300 cycles. 16S rRNA gene amplicon libraries were generated using 515F (5’-GTGCCAGCMGCCGCGGTAA-3’) and 806R (5’-GGACTACHVGGGTWTCTAAT-3’) primers for the V4 region of the 16S rRNA gene. PCR reactions were run in duplicate and agarose gel electrophoresis was used to verify specific amplification. Indexing was done according to the Kozich method^5^. Duplicate samples were combined, purified, and pooled at equimolar concentrations for sequencing on an Illumina MiSeq with a 2x250-bp paired-end run.

***Whole genome scan for DEC diagnostic virulence genes***

Endpoint PCR-based DEC pathotype designations were confirmed using a read-based scan of isolate whole-genome sequences. Trimmed isolate reads were mapped against reference pathotype virulence gene sequences (see Supplementary Table 7 for genes and pathotype designation criteria) using blastn^6^. Blastn outputs were filtered to include only reads with ≥95% query sequence identity and ≥80% query length coverage. Next, the script “[BlastTab.seqdepth_ZIP.pl](http://blasttab.seqdepth_zip.pl/)” from the enveomics collection (<http://enve-omics.ce.gatech.edu/enveomics/>)^7^ was used to calculate the observed sequencing depth and number of reads mapping to each pathotype gene. Gene presence/absence was determined by the number of reads recruited (coverage depth) and the percent gene length that was covered (coverage breadth), assuming a zero-inflated Poisson distribution to correct for non-covered positions. Genes with zero inflation values of ≥0.3, which represents the fraction of the gene that is not covered, were considered absent. Thus, only genes with at least 70% breadth of coverage were considered present. This read-based approach was chosen over homology-based designations of assembled sequences to avoid the limitations and biases of the assembly process.

***qPCR analyses***

Quantitative PCR (qPCR) assays were used to quantify total bacterial and total *E. coli* gene copy numbers for a subset of samples for which sufficient whole stool DNA template left over after gut microbiome sequencing. For all assays, samples were run in duplicate on a Bio-Rad CFX96 real-time PCR system. 20 μl final volume reaction mixtures contained 10 μl of 2X SYBR Select Master Mix (Applied Biosystems, Austin, TX), 0.25 μM each forward and reverse primer, and 4 μl of DNA template. Cycling conditions for all assays were as follows: 50°C for 2 min, 95°C for 2 min, and 40 cycles of 95°C for 15s, 60 to 61.5°C for 15s, and 72°C for 1 min. Primers and precise annealing temperatures for assays used are summarized in Supplementary Table 8.

Gene abundance was quantified by interpolation to a standard curve as the mean concentration of duplicate reactions and reported as gene copies per ng DNA template. The standard curve was generated using gBlock gene fragments (Integrated DNA Technologies, Coralville, IA) that contained the target sequence at ten-fold dilutions ranging from 10^6^ to 10^1^ gene copies per reaction. No template added negative controls were included on each qPCR plate run. An inhibition control assay was used to test for qPCR inhibition, and 2.5x10^4^ copies of an artificially designed inhibition control gene target^8^ were spiked into all samples. Ct values of the inhibition control recovered from all samples were compared to those recovered from spiked nuclease free water as a benchmark. No inhibition (defined as a Ct value difference >2) was detected. A single peak on a melt curve analysis was used to confirm amplification of a single product at the expected melting temperature.

Standard curves were analyzed according to published Minimum Information for Publication of Quantitiative Real-Time PCR Experiements (MIQE) guidelines^9,10^. Detection and quantification methods are reported as described in Nguyen *et al.* 2018^11^. Briefly, limits of detection were defined as the lowest amount of template that could be reliably detected above the negative control for each assay run. The limit of quantification was defined using the standard curve as the gene target concentration where the standard deviation for all replicates was less than or equal to 2 Ct values. Results were quantified if the duplicate reactions were both amplified, fell within 2 standard deviations of each other, and were above the level of the lowest standard. If zero or one well was amplified, the result was deemed non-detectable (ND) and designated a value of half the limit of detection. If both duplicates were positive, but amplification occurred after the lowest dilution, the result was considered detected but not quantifiable (DNQ) and assigned the value of the limit of detection. Average assay efficiency was 88% for the total bacteria assay, 90% for the total *E. coli* assay, and 99% for the inhibition control assay. The mean slope, y-intercept, and R^2^ for each assay is listed in Supplementary Table 8.


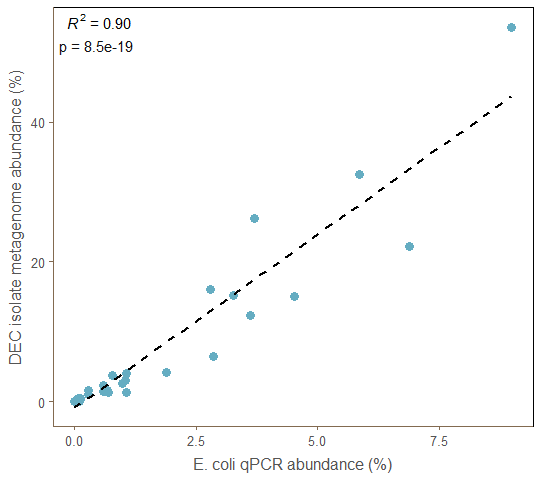


Supplementary Figure 1. Comparison of DEC isolate metagenome abundance and E. coli qPCR R abundance measurements.


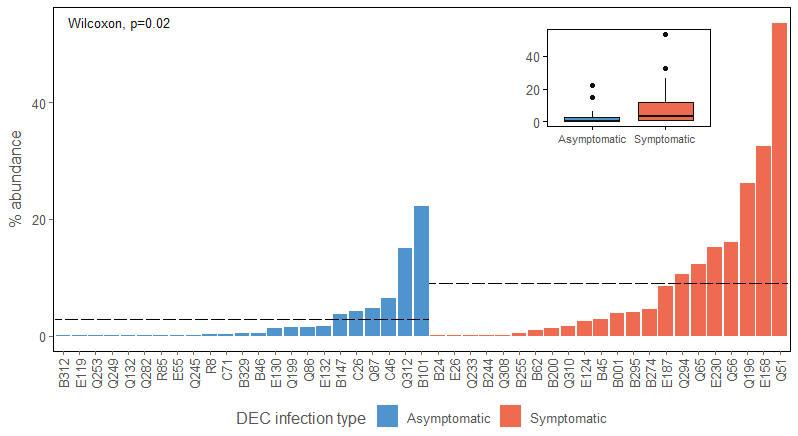


Supplementary Figure 2. DEC isolate metagenome abundances for participants aged <5 years. Dashed lines indicate mean abundance for case versus control sample groups. Data are also shown as an inset box plot.


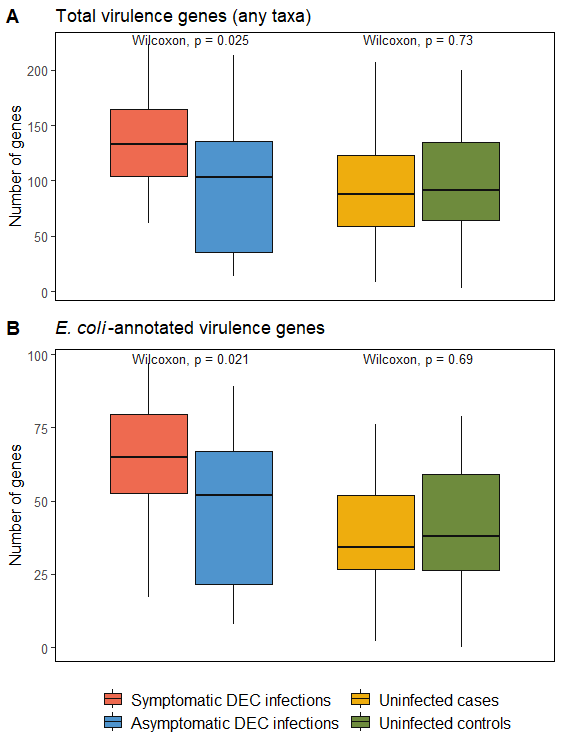


Supplementary Figure 3. Comparison of the numbers of virulence genes from the Virulence Factor Database (VFDB) in symptomatic versus asymptomatic DEC infections and in uninfected cases versus controls for participants aged <5 years. Data are shown for virulence genes in the VFDB annotated as any taxa (A) and as E. coli (B).


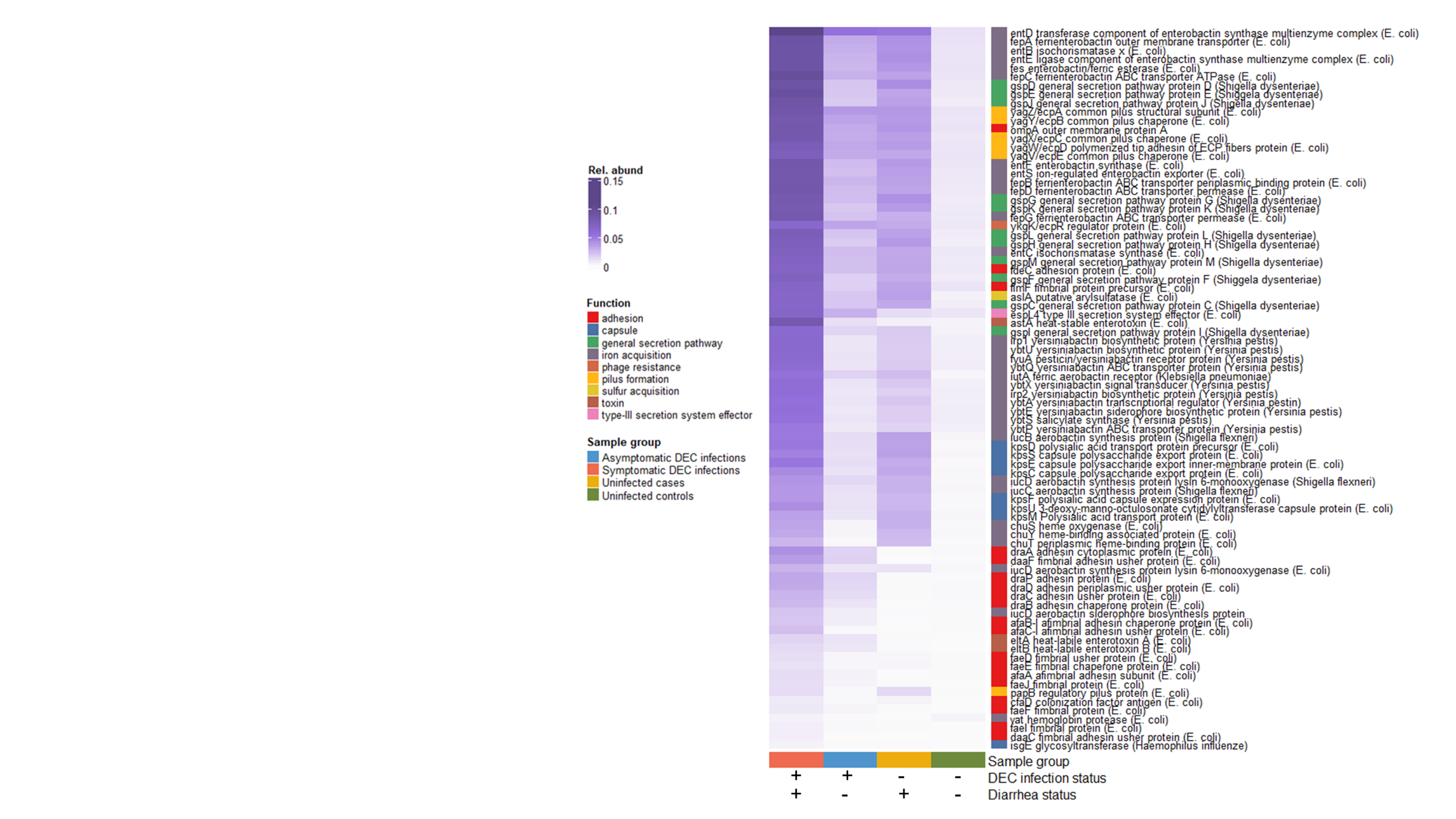


Supplementary Figure 4. Mean relative abundances of virulence genes that were significantly differentially abundant by DEC infection and diarrhea case/control status for participants <5 years (four-way Kruskal-Wallis adjusted p-values<0.05).


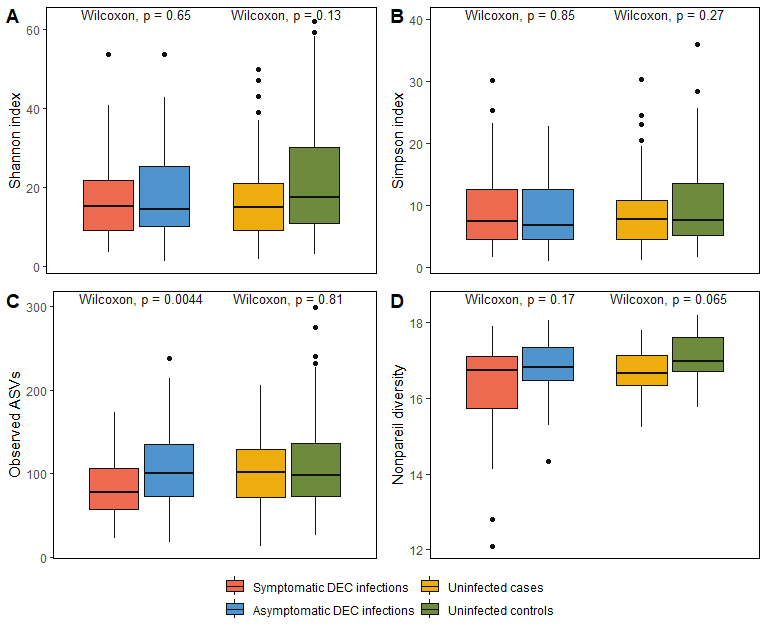


Supplementary Figure 5. Comparisons of coverage-based estimates of within-sample alpha diversity for symptomatic versus asymptomatic DEC infections and for uninfected cases versus controls with E. coli reads/ASVs filtered from the analyses. Shannon, Simpson, and Observed alpha diversity metrics were calculated using 16S rRNA data (A-C); Nonpareil alpha diversity was calculated using shotgun metagenome data (D).


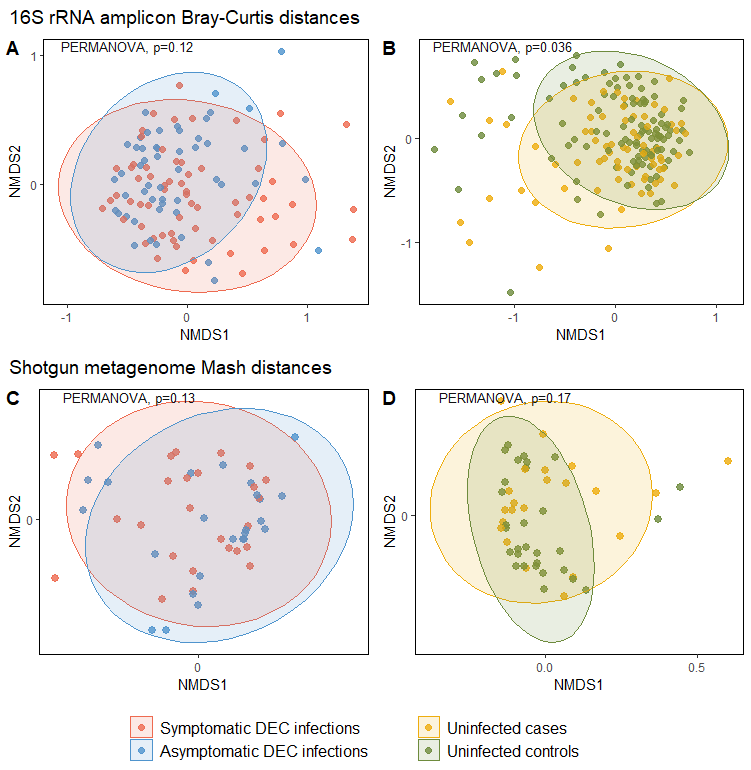


Supplementary Figure 6. NMDS plots summarizing between-sample beta diversity metrics; Bray-Curtis dissimilarity comparisons for symptomatic versus asymptomatic DEC infections (A) and uninfected cases versus controls (B) were calculated using 16S rRNA gene amplicon data; MASH distances for the same comparisons (C, D) were calculated using shotgun metagenome data.


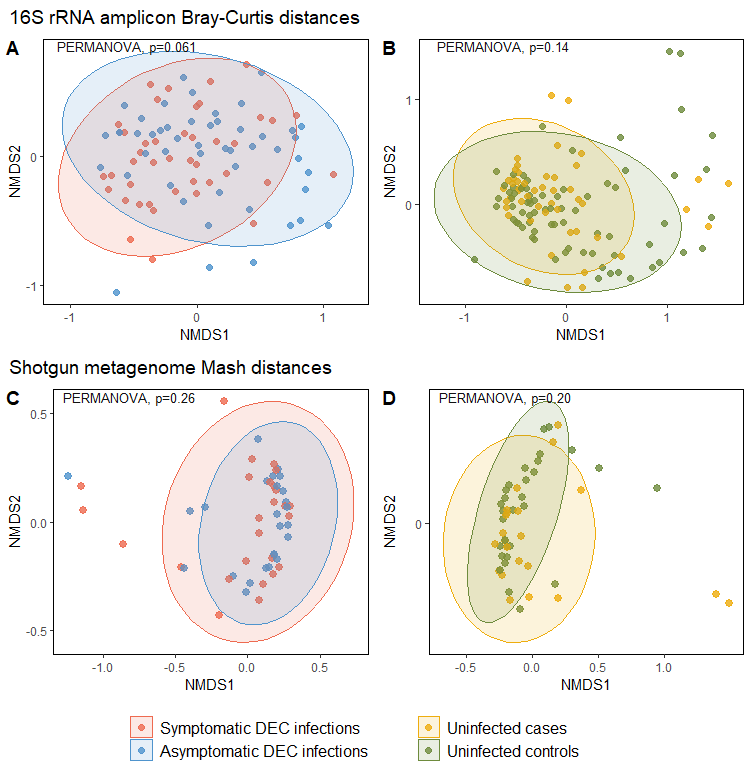


Supplementary Figure 7. NMDS plots summarizing between-sample beta diversity metrics with E. coli ASVs (16S rRNA gene amplicon data) or reads (shotgun metagenome data) removed; Bray-Curtis dissimilarity comparisons for symptomatic versus asymptomatic DEC infections (A) and uninfected cases versus controls (B) were calculated using 16S rRNA gene amplicon data; MASH distances for the same comparisons (C, D) were calculated using shotgun metagenome data.


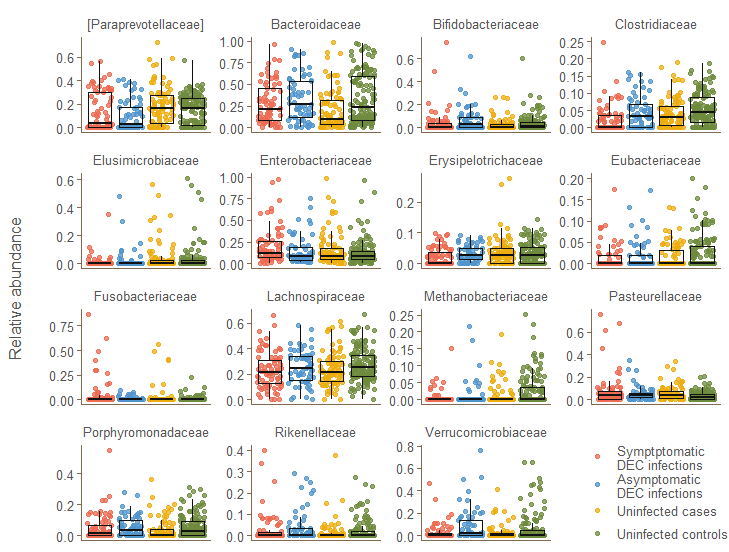


Supplementary Figure 8. Boxplots of mean relative abundances of 16S rRNA amplicon family-level taxa that were significantly associated with diarrhea and DEC infection status (corncob and/or LEfSe analyses; adjusted p<0.05, LEfSe LDA threshold<3). Y-axes are square root transformed to improve visualization of taxa with low relative abundances.


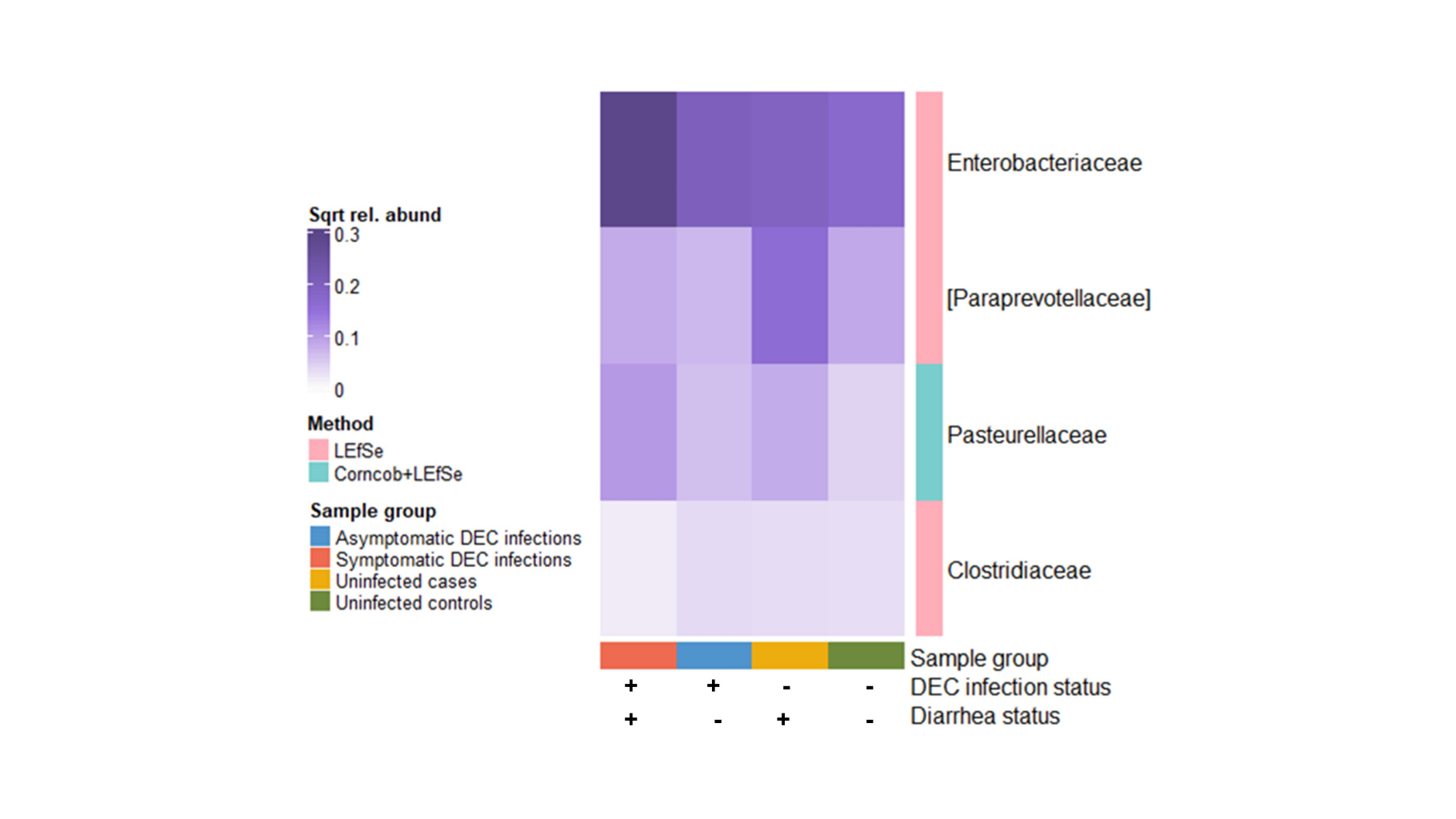


Supplementary Figure 9. Mean relative abundances of 16S rRNA amplicon gene family-level bacterial taxa that were significantly associated with diarrhea and DEC infection status for participants aged <5 years (corncob or LEfSe analyses; adjusted p<0.05, LEfSe LDA threshold <3). Values are square root transformed to improve visualization of taxa with low relative abundances.


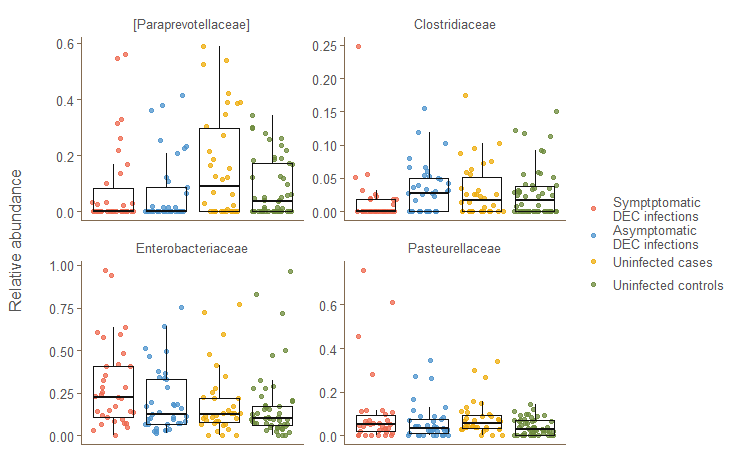


Supplementary Figure 10. Boxplots of mean relative abundances of 16S rRNA amplicon gene family-level taxa that were significantly associated with diarrhea and DEC infection status for participants aged <5 years (corncob and/or LEfSe analyses; adjusted p<0.05, LEfSe LDA threshold<3). Y-axes are square root transformed to improve visualization of taxa with low relative abundances.


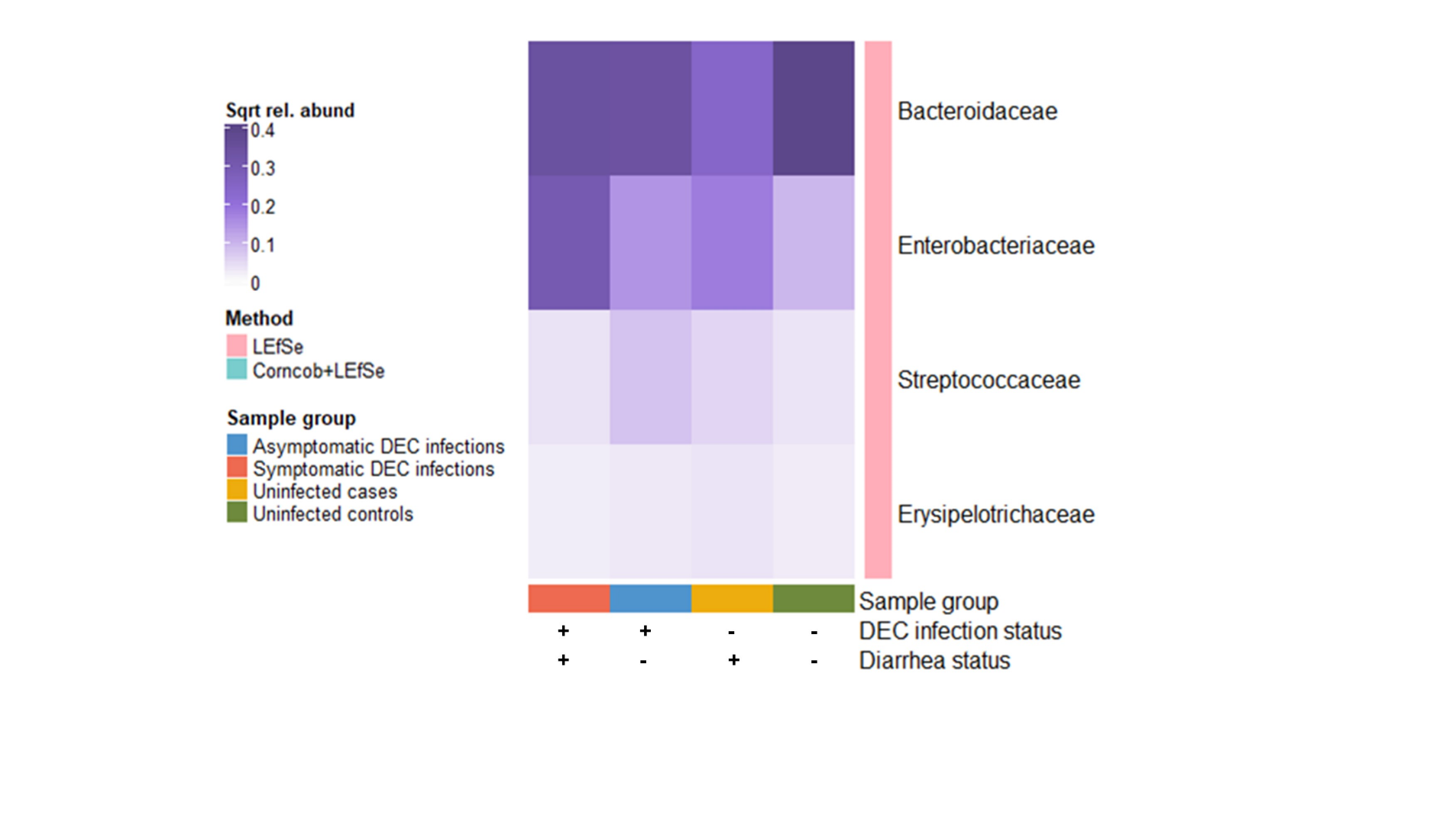


Supplementary Figure 11. Mean relative abundances of shotgun metagenome family-level bacterial taxa that were significantly associated with diarrhea and DEC infection status for participants aged <5 years (LEfSe analysis; adjusted p<0.05, LEfSe LDA threshold <3). Values are square root transformed to improve visualization of taxa with low relative abundances.


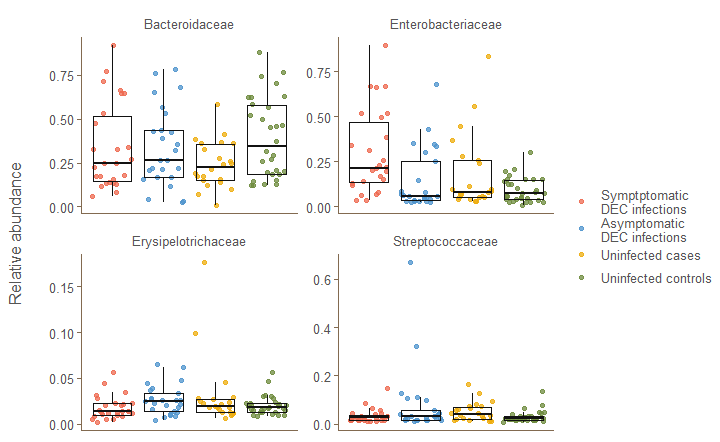


Supplementary Figure 12. Boxplots of mean relative abundances of shotgun metagenome taxa that were significantly associated with diarrhea and DEC infection status (LEfSe analysis; adjusted p<0.05, Lefse LDA threshold<3). Y-axes are square root transformed to improve visualization of taxa with low relative abundances.

| Supplementary Table 1. Endpoint PCR primer sequences for DEC virulence gene PCR screening. | | | | |
| --- | --- | --- | --- | --- |
| Gene | Forward primer (5’-3’) | Reverse primer (5’-3’) | Product size (bp) | Reference |
| *aggR* | GTATACACAAAAGAAGGAAGC | ACAGAATCGTCAGCATCAGC | 254 | Toma *et al.* 2003^1^ |
| *lt* | GCGACAAATTATACCGTGCT | CCGAATTCTGTTATATATGT | 708 | Tornieporth *et al.* 1995^2^ |
| *sta* | CTGTATTGTCTTTTTCACCT | GCACCCGGTACAAGCAGGAT | 182 | Tornieporth *et al.* 1995^2^ |
| *bfp* | CAATGGTGCTTGCGCTTGCT | GCCGCTTTATCCAACCTGGT | 324 | Tornieporth *et al.* 1995^2^ |
| *eaeA* | GACCCGGCACAAGCATAAGC | CCACCTGCAGCAACAAGAGG | 384 | ­­Paton and Paton 1998^3^ |
| *ipaH* | GCTGGAAAAACTCAGTGCCT | CCAGTCCGTAAATTCATTCT | 424 | Tornieporth *et al.* 1995^2^ |
| *afaB/afaC* | GCTGGGCAGCAAACTGATAACTCTC | CATCAAGCTCTTTGTTCGTCCGCCG | 750 | Le Bouguenec *et al.* 1992^4^ |
| *stx1* | ATAAATCGCCATTCGTTGACTAC | AGAACGCCCACTGAGATCATC | 180 | ­­­Paton and Paton 1998^3^ |
| *stx2* | GGCACTGTCTGAAACTGCTCC | TCGCCAGTTATCTGACATTCTG | 255 | ­­­Paton and Paton 1998^3^ |

| Supplementary Table 2. Summary of sample exclusion/inclusion criteria for final analyses by datatype. | | | | |
| --- | --- | --- | --- | --- |
| **Datatype** | **Number sequenced** | **Criteria** | **Number removed^a^** | **Number used in analyses** |
| DEC isolate genomes | 279 | Participant stool was rotavirus-positive | 10 | 196 |
|  |  | Genome sequence was poor quality or not annotated as *E. coli* | 15 |  |
|  |  | Disagreement between PCR and bioinformatic pathotype designations | 72 |  |
| 16S rRNA gene amplicon sequencing data | 411 | Participant stool was rotavirus-positive | 35 | 358 |
|  |  | Insufficient or missing metadata | 10 |  |
|  |  | Isolate associated with sample was contaminated | 8 |  |
| Whole shotgun metagenomes | 108 | Participant stool was rotavirus-positive | 4 | 103 |
|  |  | Isolate associated with sample was contaminated | 1 |  |
| ^a^Note that a single sample could fit multiple criteria for filtering (i.e., be both rotavirus-positive and missing metadata) | | | | |

| Supplementary Table 3. Supplemental metadata | | | | | | | | |  |  |  |  |
| --- | --- | --- | --- | --- | --- | --- | --- | --- | --- | --- | --- | --- |
| Sample ID | Alternate metagenome ID | 16S rRNA amplicon sequenced from whole stool | DEC isolate whole genome sequenced | DEC isolate ID(s) | Shotgun metagenome sequenced from whole stool | Diarrhea | DEC infection | DEC pathotype | Participant age (years) |  |  |  |
| B001 | MG1 | no | yes | B001_5 | yes | Case | Infected | DAEC | 1 |  |  |  |
| B006 | NA | yes | no | NA | no | Case | Uninfected | None | 28 |  |  |  |
| B011 | NA | yes | no | NA | no | Case | Uninfected | None | 7 |  |  |  |
| B012 | NA | no | yes | B12_1 | no | Case | Infected | DAEC | 1 |  |  |  |
| B019 | NA | yes | no | NA | no | Control | Uninfected | None | 28 |  |  |  |
| B022 | NA | yes | no | NA | no | Control | Uninfected | None | 1 |  |  |  |
| B024 | MG5 | yes | yes | B24_1 | yes | Case | Infected | DAEC | 2 |  |  |  |
| B027 | NA | yes | no | NA | no | Control | Uninfected | None | 6 |  |  |  |
| B031 | NA | yes | no | NA | no | Control | Uninfected | None | 18 |  |  |  |
| B032 | NA | yes | no | NA | no | Control | Uninfected | None | 19 |  |  |  |
| B033 | NA | yes | no | NA | no | Control | Uninfected | None | 11 |  |  |  |
| B036 | NA | yes | no | NA | no | Control | Uninfected | None | 1 |  |  |  |
| B037 | NA | yes | no | NA | no | Case | Uninfected | None | 44 |  |  |  |
| B042 | NA | yes | no | NA | no | Case | Uninfected | None | 30 |  |  |  |
| B045 | MG6 | yes | yes | B45_2 | yes | Case | Infected | ETEC | 2 |  |  |  |
| B046 | B46 | yes | yes | B46_1 | yes | Control | Infected | ETEC | 1 |  |  |  |
| B048 | NA | yes | no | NA | no | Control | Uninfected | None | 22 |  |  |  |
| B062 | MG3 | yes | yes | B62_5 | yes | Case | Infected | ETEC | 1 |  |  |  |
| B064 | MG2 | yes | no | NA | yes | Case | Uninfected | None | 26 |  |  |  |
| B066 | NA | no | yes | B66_1, B66_4 | no | Case | Infected | EAEC and EAEC | 0 |  |  |  |
| B075 | NA | no | yes | B75_4, B75_5 | no | Case | Infected | DAEC | 1 |  |  |  |
| B077 | NA | yes | no | NA | no | Case | Uninfected | None | 19 |  |  |  |
| B084 | NA | yes | yes | B84_2, B84_3 | no | Case | Infected | EIEC and ETEC | 1 |  |  |  |
| B088 | NA | yes | yes | B88_3 | no | Case | Infected | EIEC | 1 |  |  |  |
| B091 | NA | yes | no | NA | no | Case | Uninfected | None | 48 |  |  |  |
| B095 | NA | yes | yes | B95_1 | no | Case | Infected | DAEC | 0 |  |  |  |
| B097 | NA | yes | no | NA | no | Case | Uninfected | None | 46 |  |  |  |
| B099 | NA | yes | yes | B99_5 | no | Control | Infected | aEPEC | 7 |  |  |  |
| B100 | NA | yes | yes | B100_5 | no | Control | Infected | DAEC | 27 |  |  |  |
| B101 | B101 | yes | yes | B101_1 | yes | Control | Infected | DAEC | 0 |  |  |  |
| B103 | NA | yes | no | NA | no | Control | Uninfected | None | 3 |  |  |  |
| B104 | NA | yes | no | NA | no | Control | Uninfected | None | 4 |  |  |  |
| B106 | NA | yes | no | NA | no | Control | Uninfected | None | 29 |  |  |  |
| B108 | NA | yes | no | NA | no | Case | Uninfected | None | 0 |  |  |  |
| B109 | MG10 | yes | yes | B109_1 | yes | Case | Infected | ETEC | 5 |  |  |  |
| B112 | NA | yes | no | NA | no | Case | Uninfected | None | 8 |  |  |  |
| B117 | NA | yes | no | NA | no | Control | Uninfected | None | 7 |  |  |  |
| B118 | NA | yes | yes | B118_2 | no | Control | Infected | DAEC | 54 |  |  |  |
| B119 | NA | yes | yes | B119_1 | no | Case | Infected | EAEC | 0 |  |  |  |
| B122 | NA | yes | no | NA | no | Case | Uninfected | None | 18 |  |  |  |
| B124 | NA | yes | no | NA | no | Control | Uninfected | None | 4 |  |  |  |
| B126 | NA | yes | no | NA | no | Control | Uninfected | None | 15 |  |  |  |
| B129 | NA | yes | no | NA | no | Case | Uninfected | None | 5 |  |  |  |
| B135 | NA | yes | no | NA | no | Case | Uninfected | None | 4 |  |  |  |
| B141 | NA | yes | yes | B141_2 | no | Control | Infected | EAEC | 4 |  |  |  |
| B143 | NA | yes | yes | B143_4 | no | Control | Infected | EIEC | 0 |  |  |  |
| B145 | NA | yes | yes | B145_4 | no | Control | Infected | EAEC | 0 |  |  |  |
| B147 | B147 | yes | yes | B147_1 | yes | Control | Infected | DAEC | 1 |  |  |  |
| B159 | NA | yes | no | NA | no | Case | Uninfected | None | 8 |  |  |  |
| B165 | NA | yes | no | NA | no | Control | Uninfected | None | 15 |  |  |  |
| B169 | NA | no | yes | B169_1 | no | Control | Infected | EAEC | 0 |  |  |  |
| B170 | NA | yes | no | NA | no | Control | Uninfected | None | 12 |  |  |  |
| B174 | NA | yes | no | NA | no | Case | Uninfected | None | 12 |  |  |  |
| B181 | NA | yes | no | NA | no | Case | Uninfected | None | 38 |  |  |  |
| B185 | NA | yes | no | NA | no | Control | Uninfected | None | 16 |  |  |  |
| B188 | NA | yes | yes | B188_1 | no | Control | Infected | EAEC | 10 |  |  |  |
| B192 | NA | yes | no | NA | no | Control | Uninfected | None | 17 |  |  |  |
| B194 | NA | yes | no | NA | no | Case | Uninfected | None | 35 |  |  |  |
| B195 | NA | yes | no | NA | no | Case | Uninfected | None | 70 |  |  |  |
| B200 | MG15 | yes | yes | B200_2 | yes | Case | Infected | ETEC | 2 |  |  |  |
| B201 | NA | yes | yes | B201_3, 201_5 | no | Case | Infected | EAEC and EAEC | 4 |  |  |  |
| B202 | NA | yes | yes | B202_2 | no | Case | Infected | EAEC | 15 |  |  |  |
| B203 | NA | yes | no | NA | no | Control | Uninfected | None | 18 |  |  |  |
| B207 | NA | yes | yes | B207_2 | no | Case | Infected | DAEC | 12 |  |  |  |
| B209 | NA | yes | no | NA | no | Control | Uninfected | None | 20 |  |  |  |
| B210 | NA | yes | no | NA | no | Case | Uninfected | None | 46 |  |  |  |
| B211 | NA | yes | no | NA | no | Case | Uninfected | None | 6 |  |  |  |
| B212 | NA | yes | no | NA | no | Control | Uninfected | None | 15 |  |  |  |
| B213 | NA | yes | no | NA | no | Control | Uninfected | None | 1 |  |  |  |
| B214 | NA | yes | no | NA | no | Control | Uninfected | None | 37 |  |  |  |
| B216 | NA | yes | no | NA | no | Control | Uninfected | None | 23 |  |  |  |
| B217 | NA | yes | no | NA | no | Control | Uninfected | None | 20 |  |  |  |
| B219 | NA | yes | no | NA | no | Control | Uninfected | None | 38 |  |  |  |
| B225 | NA | no | yes | B225_3 | no | Control | Infected | aEPEC | 3 |  |  |  |
| B226 | NA | yes | yes | B226_1 | no | Case | Infected | DAEC | 64 |  |  |  |
| B228 | MG14 | yes | no | NA | yes | Case | Uninfected | None | 5 |  |  |  |
| B230 | NA | yes | no | NA | no | Case | Uninfected | None | 29 |  |  |  |
| B231 | NA | yes | yes | B231_1, B231_2 | no | Control | Infected | tEPEC and aEPEC | 0 |  |  |  |
| B234 | NA | yes | yes | B234_1 | no | Control | Infected | DAEC | 29 |  |  |  |
| B235 | NA | yes | yes | B235_3 | no | Case | Infected | tEPEC | 85 |  |  |  |
| B236 | NA | yes | no | NA | no | Control | Uninfected | None | 39 |  |  |  |
| B244 | MG12 | yes | yes | B244_3 | yes | Case | Infected | ETEC | 1 |  |  |  |
| B245 | NA | yes | no | NA | no | Case | Uninfected | None | 22 |  |  |  |
| B246 | NA | yes | yes | B246_1 | no | Case | Infected | aEPEC | 30 |  |  |  |
| B248 | NA | yes | no | NA | no | Case | Uninfected | None | 21 |  |  |  |
| B249 | NA | yes | no | NA | no | Case | Uninfected | None | 21 |  |  |  |
| B250 | NA | yes | no | NA | no | Case | Uninfected | None | 26 |  |  |  |
| B251 | NA | yes | no | NA | no | Case | Uninfected | None | 27 |  |  |  |
| B252 | NA | yes | no | NA | no | Case | Uninfected | None | 34 |  |  |  |
| B253 | NA | yes | no | NA | no | Case | Uninfected | None | 44 |  |  |  |
| B255 | MG11 | yes | yes | B255_1 | yes | Case | Infected | ETEC | 1 |  |  |  |
| B259 | B259 | yes | yes | B259_1 | yes | Control | Infected | aEPEC | 6 |  |  |  |
| B262 | NA | no | yes | B262_1 | no | Case | Infected | EIEC | 66 |  |  |  |
| B263 | NA | yes | no | NA | no | Control | Uninfected | None | 41 |  |  |  |
| B270 | NA | yes | yes | B270_2 | no | Case | Infected | ETEC | 44 |  |  |  |
| B273 | NA | yes | no | NA | no | Control | Uninfected | None | 45 |  |  |  |
| B274 | MG13 | yes | yes | B274_2 | yes | Case | Infected | DAEC | 1 |  |  |  |
| B276 | NA | yes | no | NA | no | Case | Uninfected | None | 36 |  |  |  |
| B278 | NA | yes | no | NA | no | Control | Uninfected | None | 25 |  |  |  |
| B279 | NA | yes | no | NA | no | Control | Uninfected | None | 25 |  |  |  |
| B280 | NA | yes | yes | B280_4 | no | Control | Infected | EAEC | 39 |  |  |  |
| B281 | NA | yes | no | NA | no | Control | Uninfected | None | 20 |  |  |  |
| B282 | NA | yes | no | NA | no | Control | Uninfected | None | 19 |  |  |  |
| B283 | NA | yes | no | NA | no | Control | Uninfected | None | 29 |  |  |  |
| B285 | NA | yes | no | NA | no | Case | Uninfected | None | 8 |  |  |  |
| B293 | B293 | yes | no | NA | yes | Control | Uninfected | None | 1 |  |  |  |
| B295 | MG16 | yes | yes | B295_2 | yes | Case | Infected | ETEC | 2 |  |  |  |
| B302 | NA | yes | no | NA | no | Control | Uninfected | None | 51 |  |  |  |
| B305 | NA | yes | no | NA | no | Control | Uninfected | None | 17 |  |  |  |
| B309 | NA | yes | yes | B309_1 | no | Case | Infected | EIEC | 3 |  |  |  |
| B311 | NA | yes | no | NA | no | Case | Uninfected | None | 66 |  |  |  |
| B312 | B312 | yes | yes | B312_4 | yes | Control | Infected | aEPEC | 1 |  |  |  |
| B313 | NA | yes | no | NA | no | Control | Uninfected | None | 33 |  |  |  |
| B323 | NA | yes | no | NA | no | Control | Uninfected | None | 1 |  |  |  |
| B327 | NA | yes | no | NA | no | Control | Uninfected | None | 12 |  |  |  |
| B328 | NA | yes | no | NA | no | Control | Uninfected | None | 70 |  |  |  |
| B329 | B329 | yes | yes | B329_2 | yes | Control | Infected | DAEC | 0 |  |  |  |
| C001 | NA | no | yes | C1_2 | no | Case | Infected | ETEC | missing |  |  |  |
| C009 | NA | yes | yes | C9_2 | no | Case | Infected | aEPEC | 7 |  |  |  |
| C013 | NA | yes | no | NA | no | Case | Uninfected | None | 2 |  |  |  |
| C014 | NA | yes | yes | C14_2A, C14_2B | no | Case | Infected | DAEC and DAEC | 9 |  |  |  |
| C019 | NA | yes | no | NA | no | Control | Uninfected | None | 4 |  |  |  |
| C021 | NA | yes | yes | C21_2 | no | Control | Infected | EAEC | 2 |  |  |  |
| C023 | NA | yes | yes | C23_1 | no | Control | Infected | EAEC | 1 |  |  |  |
| C025 | NA | yes | yes | C25_2 | no | Control | Infected | EIEC | 15 |  |  |  |
| C026 | C26 | yes | yes | C26_2 | yes | Control | Infected | aEPEC | 3 |  |  |  |
| C032 | NA | yes | no | NA | no | Case | Uninfected | None | 1 |  |  |  |
| C033 | NA | yes | yes | C33_3 | no | Case | Infected | EAEC | 4 |  |  |  |
| C034 | NA | yes | yes | C34_2 | no | Case | Infected | DAEC | 1 |  |  |  |
| C036 | NA | yes | yes | C36_1 | no | Case | Infected | DAEC | 1 |  |  |  |
| C038 | NA | yes | no | NA | no | Case | Uninfected | None | 2 |  |  |  |
| C046 | C46 | yes | yes | C46_4 | yes | Control | Infected | ETEC | 2 |  |  |  |
| C050 | NA | yes | yes | C50_4 | no | Control | Infected | DAEC | 37 |  |  |  |
| C070 | NA | yes | yes | C70_1 | no | Control | Infected | aEPEC | 1 |  |  |  |
| C071 | C71 | yes | yes | C71_2 | yes | Control | Infected | DAEC | 1 |  |  |  |
| C072 | NA | no | yes | C72_3 | no | Case | Infected | EAEC | missing |  |  |  |
| C080 | NA | yes | yes | C80_4 | no | Control | Infected | EAEC | 0 |  |  |  |
| C085 | NA | yes | no | NA | no | Case | Uninfected | None | 3 |  |  |  |
| C086 | NA | yes | no | NA | no | Control | Uninfected | None | 1 |  |  |  |
| C087 | NA | yes | no | NA | no | Case | Uninfected | None | 27 |  |  |  |
| C097 | NA | yes | no | NA | no | Case | Uninfected | None | 38 |  |  |  |
| C100 | NA | yes | no | NA | no | Case | Uninfected | None | 2 |  |  |  |
| C101 | NA | no | yes | C101_1 | no | Case | Infected | ETEC | 4 |  |  |  |
| C102 | NA | yes | yes | C102_1 | no | Control | Infected | aEPEC | 2 |  |  |  |
| C110 | NA | yes | yes | C110_5 | no | Control | Infected | aEPEC | 3 |  |  |  |
| E005 | NA | no | yes | E5_5 | no | Case | Infected | aEPEC | 1 |  |  |  |
| E013 | NA | yes | yes | E13 | no | Case | Infected | DAEC | 13 |  |  |  |
| E016 | NA | no | yes | E16_6 | no | Control | Infected | DAEC | 5 |  |  |  |
| E020 | NA | no | yes | E20_1 | no | Control | Infected | EAEC | 2 |  |  |  |
| E023 | NA | yes | no | NA | no | Control | Uninfected | None | 5 |  |  |  |
| E026 | MG18 | yes | yes | E26 | yes | Case | Infected | DAEC | 4 |  |  |  |
| E028 | NA | yes | yes | E28 | no | Case | Infected | DAEC | 13 |  |  |  |
| E029 | NA | no | yes | E29_1 | no | Case | Infected | ETEC | 19 |  |  |  |
| E033 | NA | yes | yes | E33_4 | no | Control | Infected | aEPEC | 13 |  |  |  |
| E034 | NA | yes | yes | E34 | no | Case | Infected | DAEC | 24 |  |  |  |
| E036 | NA | yes | no | NA | no | Control | Uninfected | None | 7 |  |  |  |
| E038 | NA | yes | no | NA | no | Control | Uninfected | None | 29 |  |  |  |
| E043 | NA | yes | yes | E43 | no | Case | Infected | DAEC | 30 |  |  |  |
| E044 | NA | yes | yes | E44_6 | no | Case | Infected | DAEC | 2 |  |  |  |
| E055 | E55 | yes | yes | E55_5, E55_6 | yes | Control | Infected | ETEC and DAEC | 1 |  |  |  |
| E056 | NA | yes | no | NA | no | Control | Uninfected | None | 2 |  |  |  |
| E057 | NA | yes | yes | E57 | no | Case | Infected | DAEC | 11 |  |  |  |
| E058 | NA | no | yes | E58_3 | no | Case | Infected | DAEC | 0 |  |  |  |
| E067 | NA | no | yes | E67_5 | no | Case | Infected | DAEC | 0 |  |  |  |
| E070 | E70 | yes | yes | E70_3 | yes | Control | Infected | EAEC | 8 |  |  |  |
| E071 | NA | yes | yes | E71 | no | Case | Infected | aEPEC | 28 |  |  |  |
| E072 | NA | yes | yes | E72 | no | Case | Infected | DAEC | 8 |  |  |  |
| E076 | NA | yes | yes | E76_1 | no | Control | Infected | EAEC | 58 |  |  |  |
| E082 | E82 | yes | no | NA | yes | Control | Uninfected | None | 57 |  |  |  |
| E084 | NA | yes | no | NA | no | Case | Uninfected | None | 29 |  |  |  |
| E085 | NA | yes | no | NA | no | Control | Uninfected | None | 21 |  |  |  |
| E088 | NA | no | yes | E88_3, E88_4 | no | Case | Infected | DAEC and EAEC | 7 |  |  |  |
| E089 | NA | no | yes | E89_4 | no | Case | Infected | EAEC | 12 |  |  |  |
| E092 | NA | no | yes | E92_5 | no | Control | Infected | DAEC | 0 |  |  |  |
| E093 | NA | yes | no | NA | no | Control | Uninfected | None | 4 |  |  |  |
| E100 | NA | no | yes | E100_4, E100_5 | no | Case | Infected | DAEC and DAEC | 4 |  |  |  |
| E101 | NA | no | yes | E101_1 | no | Control | Infected | DAEC | 3 |  |  |  |
| E106 | NA | no | yes | E106_1 | no | Case | Infected | EAEC | 1 |  |  |  |
| E108 | NA | yes | no | NA | no | Control | Uninfected | None | 7 |  |  |  |
| E114 | NA | yes | no | NA | no | Control | Uninfected | None | 63 |  |  |  |
| E115 | NA | yes | no | NA | no | Control | Uninfected | None | 14 |  |  |  |
| E119 | E119 | yes | yes | E119_5 | yes | Control | Infected | DAEC | 3 |  |  |  |
| E124 | MG23 | yes | yes | E124_5, E124_6 | yes | Case | Infected | DAEC and EAEC | 1 |  |  |  |
| E129 | NA | no | yes | E129_3 | no | Case | Infected | aEPEC | 0 |  |  |  |
| E130 | E130 | yes | no | NA | yes | Control | Uninfected | None | 1 |  |  |  |
| E131 | NA | yes | no | NA | no | Control | Uninfected | None | 6 |  |  |  |
| E132 | E132 | yes | no | NA | yes | Control | Uninfected | None | 1 |  |  |  |
| E135 | NA | yes | yes | E135_2, E135_5, E135_6 | no | Case | Infected | EAEC, EAEC, and DAEC | 10 |  |  |  |
| E139 | NA | yes | yes | E139_2 | no | Control | Infected | ETEC | 1 |  |  |  |
| E140 | NA | yes | yes | E140 | no | Case | Infected | DAEC | 29 |  |  |  |
| E141 | NA | yes | no | NA | no | Control | Uninfected | None | 4 |  |  |  |
| E143 | NA | yes | no | NA | no | Control | Uninfected | None | 36 |  |  |  |
| E144 | NA | yes | yes | E144 | no | Case | Infected | aEPEC | 69 |  |  |  |
| E153 | NA | yes | no | NA | no | Control | Uninfected | None | 8 |  |  |  |
| E158 | MG24 | yes | yes | E158 | yes | Case | Infected | DAEC | 1 |  |  |  |
| E162 | MG22 | yes | yes | E162 | yes | Case | Infected | aEPEC | 6 |  |  |  |
| E166 | NA | yes | yes | E166 | no | Case | Infected | DAEC | 10 |  |  |  |
| E167 | E167 | yes | yes | E167_5 | yes | Control | Infected | DAEC | 8 |  |  |  |
| E170 | NA | yes | yes | E170 | no | Case | Infected | DAEC | 18 |  |  |  |
| E171 | NA | yes | no | NA | no | Control | Uninfected | None | 0 |  |  |  |
| E173 | NA | yes | yes | E173 | no | Case | Infected | DAEC | 77 |  |  |  |
| E175 | NA | yes | yes | E175_4 | no | Control | Infected | EAEC | 2 |  |  |  |
| E177 | NA | yes | yes | E177_6 | no | Case | Infected | DAEC | 0 |  |  |  |
| E184 | MG19 | yes | no | NA | yes | Case | Uninfected | None | 1 |  |  |  |
| E185 | NA | yes | no | NA | no | Control | Uninfected | None | 23 |  |  |  |
| E187 | MG21 | yes | yes | E187_2 | yes | Case | Infected | tEPEC | 1 |  |  |  |
| E188 | NA | yes | yes | E188_2 | no | Case | Infected | DAEC | 13 |  |  |  |
| E189 | NA | yes | no | NA | no | Control | Uninfected | None | 9 |  |  |  |
| E192 | NA | yes | yes | E192 | no | Case | Infected | DAEC | 0 |  |  |  |
| E195 | NA | no | yes | E195_5 | no | Case | Infected | aEPEC | 1 |  |  |  |
| E197 | NA | no | yes | E197_5 | no | Case | Infected | DAEC | 1 |  |  |  |
| E204 | NA | yes | no | NA | no | Control | Uninfected | None | 1 |  |  |  |
| E205 | MG20 | yes | no | NA | yes | Case | Uninfected | None | 1 |  |  |  |
| E206 | NA | yes | no | NA | no | Control | Uninfected | None | 60 |  |  |  |
| E212 | NA | yes | yes | E212_4 | no | Control | Infected | DAEC | 66 |  |  |  |
| E215 | NA | yes | no | NA | no | Control | Uninfected | None | 2 |  |  |  |
| E218 | NA | yes | yes | E218 | no | Case | Infected | DAEC | 15 |  |  |  |
| E228 | NA | yes | yes | E228_3 | no | Control | Infected | EAEC | 0 |  |  |  |
| E230 | MG25 | yes | yes | E230_4 | yes | Case | Infected | DAEC | 1 |  |  |  |
| E236 | NA | yes | yes | E236_4 | no | Control | Infected | EAEC | 1 |  |  |  |
| E238 | NA | no | yes | E238_3, E238_5 | no | Control | Infected | EAEC and DAEC | 0 |  |  |  |
| E240 | NA | no | yes | E240_5 | no | Control | Infected | aEPEC | 0 |  |  |  |
| Q016 | NA | no | yes | Q16_1 | no | Case | Infected | ETEC | 60 |  |  |  |
| Q021 | NA | no | yes | Q21_2 | no | Case | Infected | DAEC | 47 |  |  |  |
| Q023 | NA | no | yes | Q23_1 | no | Case | Infected | DAEC | 41 |  |  |  |
| Q027 | NA | no | yes | Q27_1 | no | Control | Infected | aEPEC | 2 |  |  |  |
| Q031 | NA | no | yes | Q31_6 | no | Case | Infected | DAEC | 27 |  |  |  |
| Q033 | NA | no | yes | Q33_6 | no | Case | Infected | EIEC | 5 |  |  |  |
| Q034 | NA | no | yes | Q34_6 | no | Case | Infected | EIEC | 20 |  |  |  |
| Q035 | NA | no | yes | Q35_1 | no | Case | Infected | DAEC | 43 |  |  |  |
| Q037 | NA | no | yes | Q37_4 | no | Control | Infected | aEPEC | 34 |  |  |  |
| Q038 | NA | no | yes | Q38_1 | no | Case | Infected | ETEC | 38 |  |  |  |
| Q040 | NA | yes | yes | Q40 | no | Case | Infected | DAEC | 38 |  |  |  |
| Q049 | MG28 | yes | no | NA | yes | Case | Uninfected | None | 1 |  |  |  |
| Q051 | MG31 | yes | yes | Q51 | yes | Case | Infected | DAEC | 4 |  |  |  |
| Q053 | MG34 | yes | yes | Q53 | yes | Case | Infected | ETEC | 5 |  |  |  |
| Q056 | MG29 | yes | yes | Q56 | yes | Case | Infected | DAEC | 1 |  |  |  |
| Q065 | MG30 | yes | yes | Q65 | yes | Case | Infected | DAEC | 2 |  |  |  |
| Q069 | NA | yes | no | NA | no | Control | Uninfected | None | 49 |  |  |  |
| Q070 | NA | yes | no | NA | no | Control | Uninfected | None | 32 |  |  |  |
| Q071 | MG55 | yes | no | NA | yes | Case | Uninfected | None | 1 |  |  |  |
| Q083 | NA | yes | no | NA | no | Control | Uninfected | None | 40 |  |  |  |
| Q085 | NA | no | yes | Q85_1 | no | Case | Infected | DAEC | 10 |  |  |  |
| Q086 | MG41 | no | yes | Q86 | yes | Case | Infected | aEPEC | 1 |  |  |  |
| Q087 | MG42 | yes | yes | Q87 | yes | Control | Infected | ETEC | 1 |  |  |  |
| Q089 | NA | yes | yes | Q89 | no | Case | Infected | EAEC | 31 |  |  |  |
| Q090 | NA | yes | no | NA | no | Case | Uninfected | None | 27 |  |  |  |
| Q091 | NA | yes | yes | Q91 | no | Case | Infected | DAEC | 26 |  |  |  |
| Q097 | NA | yes | no | NA | no | Control | Uninfected | None | 41 |  |  |  |
| Q098 | NA | yes | no | NA | no | Control | Uninfected | None | 61 |  |  |  |
| Q099 | NA | yes | no | NA | no | Control | Uninfected | None | 47 |  |  |  |
| Q101 | MG49 | yes | no | NA | yes | Control | Uninfected | None | 4 |  |  |  |
| Q105 | MG37 | no | no | NA | yes | Control | Uninfected | None | 1 |  |  |  |
| Q106 | NA | yes | yes | Q106 | no | Control | Infected | ETEC | 7 |  |  |  |
| Q107 | NA | yes | no | NA | no | Control | Uninfected | None | 0 |  |  |  |
| Q108 | NA | yes | yes | Q108 | no | Case | Infected | EIEC | 10 |  |  |  |
| Q116 | MG43 | yes | no | NA | yes | Control | Uninfected | None | 2 |  |  |  |
| Q117 | NA | yes | no | NA | no | Control | Uninfected | None | 10 |  |  |  |
| Q127 | MG38 | yes | no | NA | yes | Control | Uninfected | None | 1 |  |  |  |
| Q128 | NA | yes | yes | Q128 | no | Case | Infected | EIEC | 19 |  |  |  |
| Q130 | NA | yes | no | NA | no | Control | Uninfected | None | 45 |  |  |  |
| Q131 | MG52 | yes | no | NA | yes | Control | Uninfected | None | 5 |  |  |  |
| Q132 | MG47 | yes | yes | Q132 | yes | Control | Infected | ETEC | 3 |  |  |  |
| Q133 | NA | yes | no | NA | no | Control | Uninfected | None | 31 |  |  |  |
| Q142 | NA | yes | yes | Q142 | no | Case | Infected | DAEC | 33 |  |  |  |
| Q143 | NA | yes | no | NA | no | Control | Uninfected | None | 6 |  |  |  |
| Q144 | NA | yes | no | NA | no | Control | Uninfected | None | 6 |  |  |  |
| Q145 | NA | yes | yes | Q145 | no | Control | Infected | DAEC | 30 |  |  |  |
| Q146 | NA | yes | no | NA | no | Control | Uninfected | None | 46 |  |  |  |
| Q147 | NA | yes | yes | Q147 | no | Case | Infected | DAEC | 31 |  |  |  |
| Q148 | NA | yes | no | NA | no | Control | Uninfected | None | 9 |  |  |  |
| Q157 | MG44 | yes | no | NA | yes | Control | Uninfected | None | 2 |  |  |  |
| Q158 | MG39 | yes | no | NA | yes | Control | Uninfected | None | 1 |  |  |  |
| Q159 | NA | yes | no | NA | no | Control | Uninfected | None | 17 |  |  |  |
| Q168 | NA | yes | no | NA | no | Control | Uninfected | None | 5 |  |  |  |
| Q170 | NA | yes | no | NA | no | Control | Uninfected | None | 12 |  |  |  |
| Q174 | NA | yes | no | NA | no | Control | Uninfected | None | 0 |  |  |  |
| Q178 | NA | yes | no | NA | no | Control | Uninfected | None | 11 |  |  |  |
| Q186 | NA | yes | yes | Q186 | no | Control | Infected | EAEC | 8 |  |  |  |
| Q188 | NA | yes | no | NA | no | Control | Uninfected | None | 0 |  |  |  |
| Q189 | NA | yes | no | NA | no | Control | Uninfected | None | 0 |  |  |  |
| Q192 | NA | yes | no | NA | no | Control | Uninfected | None | 25 |  |  |  |
| Q196 | MG32 | yes | yes | Q196 | yes | Case | Infected | DAEC | 4 |  |  |  |
| Q199 | Q199 | yes | yes | Q199 | yes | Control | Infected | DAEC | 0 |  |  |  |
| Q203 | NA | yes | no | NA | no | Control | Uninfected | None | 0 |  |  |  |
| Q212 | NA | yes | yes | Q212 | no | Control | Infected | EAEC | 12 |  |  |  |
| Q223 | NA | yes | yes | Q223 | no | Case | Infected | DAEC | 40 |  |  |  |
| Q233 | MG35 | yes | yes | Q233 | yes | Case | Infected | aEPEC | 2 |  |  |  |
| Q240 | NA | yes | yes | Q240 | no | Case | Infected | DAEC | 44 |  |  |  |
| Q243 | NA | yes | yes | Q243 | no | Case | Infected | DAEC | 43 |  |  |  |
| Q245 | MG48 | yes | yes | Q245_2 | yes | Control | Infected | DAEC | 3 |  |  |  |
| Q249 | MG51 | yes | yes | Q249 | yes | Control | Infected | aEPEC | 4 |  |  |  |
| Q250 | NA | no | yes | Q250_1 | no | Control | Infected | EAEC | 0 |  |  |  |
| Q253 | MG45 | yes | yes | Q253 | yes | Control | Infected | aEPEC | 2 |  |  |  |
| Q259 | NA | yes | no | NA | no | Control | Uninfected | None | 26 |  |  |  |
| Q270 | NA | yes | no | NA | no | Case | Uninfected | None | 20 |  |  |  |
| Q275 | NA | yes | yes | Q275 | no | Control | Infected | aEPEC | 49 |  |  |  |
| Q279 | NA | no | yes | Q279_1 | no | Case | Infected | EIEC | 12 |  |  |  |
| Q282 | MG46 | yes | yes | Q282 | yes | Control | Infected | aEPEC | 2 |  |  |  |
| Q284 | MG40 | yes | no | NA | yes | Control | Uninfected | None | 1 |  |  |  |
| Q288 | NA | yes | yes | Q288 | no | Control | Infected | ETEC | 8 |  |  |  |
| Q289 | NA | yes | no | NA | no | Control | Uninfected | None | 30 |  |  |  |
| Q294 | MG33 | yes | yes | Q294 | yes | Case | Infected | ETEC | 1 |  |  |  |
| Q295 | NA | yes | yes | Q295 | no | Case | Infected | ETEC | 78 |  |  |  |
| Q300 | MG36 | yes | no | NA | yes | Case | Uninfected | None | 4 |  |  |  |
| Q307 | NA | no | yes | Q307_1 | no | Control | Infected | DAEC | 0 |  |  |  |
| Q308 | MG26 | yes | yes | Q308 | yes | Case | Infected | DAEC | 1 |  |  |  |
| Q310 | MG27 | yes | yes | Q310 | yes | Case | Infected | DAEC | 1 |  |  |  |
| Q312 | Q312 | yes | yes | Q312_1 | yes | Control | Infected | DAEC | 0 |  |  |  |
| R001 | MG77 | yes | no | NA | yes | Case | Uninfected | None | 1 |  |  |  |
| R003 | NA | yes | no | NA | no | Case | Uninfected | None | 3 |  |  |  |
| R006 | MG87 | yes | no | NA | yes | Case | Uninfected | None | 2 |  |  |  |
| R007 | MG79 | no | no | NA | yes | Case | Uninfected | None | 1 |  |  |  |
| R008 | R8 | yes | yes | R8_4 | yes | Control | Infected | DAEC | 1 |  |  |  |
| R009 | MG81 | yes | no | NA | yes | Case | Uninfected | None | 2 |  |  |  |
| R010 | NA | yes | no | NA | no | Case | Uninfected | None | 4 |  |  |  |
| R011 | NA | yes | no | NA | no | Case | Uninfected | None | 57 |  |  |  |
| R012 | NA | yes | no | NA | no | Control | Uninfected | None | 1 |  |  |  |
| R013 | NA | yes | no | NA | no | Case | Uninfected | None | 54 |  |  |  |
| R014 | MG76 | yes | no | NA | yes | Case | Uninfected | None | 1 |  |  |  |
| R015 | MG59 | yes | no | NA | yes | Control | Uninfected | None | 3 |  |  |  |
| R017 | NA | yes | yes | R17_2 | no | Case | Infected | ETEC | 37 |  |  |  |
| R021 | NA | yes | no | NA | no | Case | Uninfected | None | 5 |  |  |  |
| R022 | MG60 | yes | no | NA | yes | Control | Uninfected | None | 1 |  |  |  |
| R024 | NA | yes | no | NA | no | Control | Uninfected | None | 39 |  |  |  |
| R025 | MG71 | yes | no | NA | yes | Control | Uninfected | None | 4 |  |  |  |
| R026 | MG61 | yes | no | NA | yes | Control | Uninfected | None | 2 |  |  |  |
| R029 | MG72 | yes | no | NA | yes | Control | Uninfected | None | 5 |  |  |  |
| R030 | NA | yes | no | NA | no | Control | Uninfected | None | 8 |  |  |  |
| R031 | NA | yes | no | NA | no | Case | Uninfected | None | 33 |  |  |  |
| R032 | NA | yes | no | NA | no | Case | Uninfected | None | 5 |  |  |  |
| R036 | NA | no | yes | R36_1 | no | Case | Infected | EAEC | 1 |  |  |  |
| R039 | NA | yes | no | NA | no | Case | Uninfected | None | 32 |  |  |  |
| R040 | MG73 | yes | no | NA | yes | Control | Uninfected | None | 4 |  |  |  |
| R041 | NA | yes | no | NA | no | Control | Uninfected | None | 6 |  |  |  |
| R042 | NA | yes | yes | R42_2 | no | Case | Infected | DAEC | 30 |  |  |  |
| R043 | NA | yes | yes | R43_2 | no | Control | Infected | aEPEC | 2 |  |  |  |
| R044 | NA | yes | no | NA | no | Case | Uninfected | None | 9 |  |  |  |
| R045 | NA | yes | no | NA | no | Case | Uninfected | None | 4 |  |  |  |
| R046 | NA | yes | yes | R46_3 | no | Control | Infected | ETEC | 5 |  |  |  |
| R050 | MG88 | yes | no | NA | yes | Case | Uninfected | None | 3 |  |  |  |
| R051 | NA | yes | no | NA | no | Case | Uninfected | None | 5 |  |  |  |
| R053 | NA | yes | no | NA | no | Control | Uninfected | None | 15 |  |  |  |
| R054 | R54 | yes | no | NA | no | Control | Uninfected | None | 15 |  |  |  |
| R055 | NA | yes | yes | R55_1 | yes | Control | Infected | aEPEC | 15 |  |  |  |
| R056 | NA | yes | yes | R56_3 | no | Control | Infected | EAEC | 15 |  |  |  |
| R057 | NA | yes | no | NA | no | Case | Uninfected | None | 4 |  |  |  |
| R058 | NA | yes | no | NA | no | Control | Uninfected | None | 13 |  |  |  |
| R059 | NA | yes | no | NA | no | Case | Uninfected | None | 6 |  |  |  |
| R060 | NA | yes | no | NA | no | Case | Uninfected | None | 38 |  |  |  |
| R061 | NA | yes | no | NA | no | Control | Uninfected | None | 37 |  |  |  |
| R062 | NA | yes | no | NA | no | Case | Uninfected | None | 4 |  |  |  |
| R063 | NA | yes | no | NA | no | Case | Uninfected | None | 8 |  |  |  |
| R064 | NA | yes | no | NA | no | Control | Uninfected | None | 16 |  |  |  |
| R065 | NA | yes | no | NA | no | Case | Uninfected | None | 51 |  |  |  |
| R066 | NA | yes | yes | R66_4 | no | Case | Infected | ETEC | 8 |  |  |  |
| R067 | NA | yes | yes | R67_3 | no | Control | Infected | DAEC | 10 |  |  |  |
| R068 | NA | yes | no | NA | no | Control | Uninfected | None | 12 |  |  |  |
| R071 | NA | yes | no | NA | no | Case | Uninfected | None | 5 |  |  |  |
| R074 | NA | yes | no | NA | no | Control | Uninfected | None | 9 |  |  |  |
| R076 | NA | yes | no | NA | no | Case | Uninfected | None | 0 |  |  |  |
| R077 | NA | yes | no | NA | no | Case | Uninfected | None | 0 |  |  |  |
| R078 | MG82 | yes | no | NA | yes | Case | Uninfected | None | 2 |  |  |  |
| R079 | MG83 | yes | no | NA | yes | Case | Uninfected | None | 2 |  |  |  |
| R080 | MG62 | yes | no | NA | yes | Control | Uninfected | None | 1 |  |  |  |
| R081 | MG63 | yes | no | NA | yes | Control | Uninfected | None | 1 |  |  |  |
| R083 | NA | yes | yes | R83_3 | no | Control | Infected | EAEC | 2 |  |  |  |
| R084 | R84 | yes | no | NA | yes | Control | Uninfected | None | 1 |  |  |  |
| R085 | R85 | yes | yes | R85_2 | yes | Control | Infected | ETEC | 2 |  |  |  |
| R086 | NA | no | yes | R86_1 | no | Control | Infected | DAEC | 80 |  |  |  |
| R088 | MG80 | yes | no | NA | yes | Case | Uninfected | None | 1 |  |  |  |
| R090 | MG74 | yes | no | NA | yes | Control | Uninfected | None | 1 |  |  |  |
| R091 | MG64 | yes | no | NA | yes | Control | Uninfected | None | 4 |  |  |  |
| R093 | MG84 | yes | no | NA | yes | Case | Uninfected | None | 2 |  |  |  |
| R097 | MG75 | yes | no | NA | yes | Control | Uninfected | None | 2 |  |  |  |
| R098 | NA | yes | no | NA | no | Control | Uninfected | None | 8 |  |  |  |
| R101 | MG78 | yes | no | NA | yes | Case | Uninfected | None | 1 |  |  |  |
| R102 | NA | yes | no | NA | no | Case | Uninfected | None | 0 |  |  |  |
| R104 | NA | yes | no | NA | no | Control | Uninfected | None | 9 |  |  |  |
| R105 | MG65 | yes | no | NA | yes | Control | Uninfected | None | 3 |  |  |  |
| R109 | NA | yes | no | NA | no | Control | Uninfected | None | 1 |  |  |  |
| R110 | NA | yes | no | NA | no | Control | Uninfected | None | 0 |  |  |  |
| R111 | MG86 | yes | no | NA | yes | Case | Uninfected | None | 2 |  |  |  |
| R113 | NA | yes | yes | R113_1, R113_3 | no | Case | Infected | ETEC and DAEC | 0 |  |  |  |
| R114 | MG85 | yes | no | NA | yes | Case | Uninfected | None | 2 |  |  |  |
| R116 | NA | yes | yes | R116_2 | no | Control | Infected | ETEC | 17 |  |  |  |
| R118 | NA | yes | no | NA | no | Case | Uninfected | None | 57 |  |  |  |
| R119 | R119 | yes | yes | R119_3 | yes | Control | Infected | ETEC | 7 |  |  |  |
| R120 | NA | yes | no | NA | no | Case | Uninfected | None | 45 |  |  |  |
| R122 | NA | no | yes | R122_4 | no | Case | Infected | EAEC | 11 |  |  |  |
| R123 | NA | yes | no | NA | no | Control | Uninfected | None | 0 |  |  |  |
| R124 | MG68 | yes | no | NA | yes | Control | Uninfected | None | 2 |  |  |  |
| R125 | NA | yes | no | NA | no | Case | Uninfected | None | 7 |  |  |  |
| R126 | MG57 | yes | no | NA | yes | Case | Uninfected | None | 1 |  |  |  |
| R127 | NA | yes | yes | R127_3 | no | Case | Infected | aEPEC | 7 |  |  |  |
| R128 | NA | yes | no | NA | no | Control | Uninfected | None | 6 |  |  |  |
| R129 | MG69 | yes | no | NA | yes | Control | Uninfected | None | 2 |  |  |  |
| R130 | MG66 | yes | no | NA | yes | Control | Uninfected | None | 5 |  |  |  |
| R131 | MG70 | yes | no | NA | yes | Control | Uninfected | None | 4 |  |  |  |
| R132 | NA | yes | no | NA | no | Control | Uninfected | None | 7 |  |  |  |
| R134 | MG67 | yes | no | NA | yes | Control | Uninfected | None | 1 |  |  |  |
| R135 | MG58 | yes | no | NA | yes | Case | Uninfected | None | 1 |  |  |  |
| R136 | NA | yes | no | NA | no | Case | Uninfected | None | 59 |  |  |  |
| R137 | NA | yes | no | NA | no | Control | Uninfected | None | 2 |  |  |  |
| R138 | NA | yes | no | NA | no | Case | Uninfected | None | 14 |  |  |  |

| Supplementary Table 4**.** Shotgun metagenome sequencing and assembly metrics | | | | | |
| --- | --- | --- | --- | --- | --- |
| Sample ID | Raw reads | QC trimmed and human filtered reads | MiGA assembly N50 | MiGA assembly contigs <1k | Microbe Census genome equivalents |
| R079 | 9.66E+06 | 7.62E+06 | 5.41E+03 | 9.43E+03 | 295.8 |
| Q249 | 1.03E+07 | 7.69E+06 | 8.80E+03 | 6.73E+03 | 278.8 |
| E026 | 1.07E+07 | 8.16E+06 | 9.58E+03 | 1.53E+04 | 277.9 |
| Q105 | 1.08E+07 | 8.03E+06 | 4.56E+04 | 6.07E+03 | 247.9 |
| R090 | 1.13E+07 | 8.43E+06 | 1.73E+04 | 8.88E+03 | 304.3 |
| R093 | 1.14E+07 | 8.50E+06 | 7.86E+03 | 8.11E+03 | 295.5 |
| B101 | 1.21E+07 | 7.60E+06 | 2.89E+04 | 2.72E+03 | 279.2 |
| R081 | 1.21E+07 | 1.01E+07 | 1.49E+04 | 8.73E+03 | 297.0 |
| R078 | 1.22E+07 | 9.57E+06 | 6.76E+03 | 1.06E+04 | 378.4 |
| C071 | 1.25E+07 | 3.86E+06 | 4.96E+03 | 6.73E+03 | 151.1 |
| E184 | 1.26E+07 | 1.04E+07 | 6.04E+03 | 1.48E+04 | 433.8 |
| Q157 | 1.30E+07 | 1.03E+07 | 8.57E+03 | 9.43E+03 | 249.2 |
| C026 | 1.30E+07 | 9.65E+06 | 6.96E+03 | 1.81E+04 | 380.0 |
| B001 | 1.31E+07 | 2.30E+06 | 1.08E+04 | 2.34E+03 | 90.8 |
| R111 | 1.31E+07 | 1.04E+07 | 1.50E+04 | 1.10E+04 | 411.8 |
| R088 | 1.36E+07 | 1.05E+07 | 5.99E+03 | 2.02E+04 | 438.1 |
| E167 | 1.36E+07 | 1.04E+07 | 6.24E+03 | 1.71E+04 | 397.4 |
| Q310 | 1.39E+07 | 1.08E+07 | 4.42E+03 | 6.34E+03 | 97.7 |
| Q053 | 1.39E+07 | 7.31E+06 | 4.16E+03 | 1.08E+04 | 244.3 |
| E162 | 1.39E+07 | 8.01E+06 | 5.60E+03 | 1.46E+04 | 346.9 |
| E205 | 1.41E+07 | 1.08E+07 | 9.13E+03 | 8.23E+03 | 260.2 |
| R080 | 1.41E+07 | 1.15E+07 | 9.86E+03 | 9.38E+03 | 340.3 |
| R022 | 1.41E+07 | 1.09E+07 | 1.00E+04 | 1.55E+04 | 441.3 |
| R006 | 1.43E+07 | 1.19E+07 | 6.63E+03 | 3.91E+03 | 358.2 |
| R008 | 1.44E+07 | 1.08E+07 | 4.77E+03 | 1.70E+04 | 452.4 |
| Q049 | 1.45E+07 | 1.15E+07 | 7.84E+03 | 1.80E+04 | 356.0 |
| B295 | 1.48E+07 | 1.19E+07 | 1.11E+04 | 7.91E+03 | 336.3 |
| B274 | 1.49E+07 | 1.17E+07 | 9.96E+03 | 4.27E+03 | 294.3 |
| Q086 | 1.49E+07 | 1.18E+07 | 1.07E+04 | 1.19E+04 | 286.6 |
| R131 | 1.49E+07 | 1.23E+07 | 6.62E+03 | 1.34E+04 | 443.7 |
| R050 | 1.50E+07 | 1.27E+07 | 3.78E+03 | 2.07E+04 | 707.9 |
| Q158 | 1.50E+07 | 1.16E+07 | 7.14E+03 | 1.74E+04 | 457.1 |
| R007 | 1.50E+07 | 1.21E+07 | 8.25E+03 | 1.24E+04 | 688.5 |
| R029 | 1.51E+07 | 1.28E+07 | 3.54E+03 | 3.57E+04 | 591.3 |
| R026 | 1.52E+07 | 1.28E+07 | 6.34E+03 | 1.31E+04 | 519.3 |
| C046 | 1.52E+07 | 1.15E+07 | 4.35E+03 | 2.18E+04 | 415.6 |
| Q284 | 1.52E+07 | 1.18E+07 | 2.86E+04 | 4.42E+03 | 265.8 |
| R009 | 1.53E+07 | 1.23E+07 | 9.75E+03 | 1.17E+04 | 588.4 |
| Q127 | 1.56E+07 | 1.26E+07 | 1.04E+04 | 1.18E+04 | 443.2 |
| R129 | 1.56E+07 | 1.33E+07 | 3.29E+03 | 2.52E+04 | 594.2 |
| B046 | 1.56E+07 | 1.20E+07 | 7.48E+03 | 1.61E+04 | 445.5 |
| Q131 | 1.58E+07 | 1.29E+07 | 3.53E+03 | 2.58E+04 | 685.7 |
| Q282 | 1.59E+07 | 1.31E+07 | 6.32E+03 | 1.70E+04 | 488.8 |
| R001 | 1.63E+07 | 1.35E+07 | 8.99E+03 | 7.65E+03 | 569.1 |
| B329 | 1.66E+07 | 1.28E+07 | 4.73E+04 | 6.23E+02 | 455.8 |
| B244 | 1.66E+07 | 1.16E+07 | 5.53E+03 | 1.17E+04 | 445.1 |
| R114 | 1.66E+07 | 1.38E+07 | 3.73E+03 | 2.15E+04 | 602.6 |
| Q116 | 1.68E+07 | 1.35E+07 | 1.16E+04 | 1.78E+04 | 619.7 |
| E158 | 1.68E+07 | 1.18E+07 | 1.14E+04 | 8.64E+03 | 379.0 |
| R015 | 1.70E+07 | 1.40E+07 | 4.50E+03 | 2.32E+04 | 620.8 |
| R040 | 1.72E+07 | 1.45E+07 | 5.95E+03 | 1.20E+04 | 544.6 |
| E230 | 1.76E+07 | 1.46E+07 | 5.53E+03 | 1.04E+04 | 484.6 |
| B062 | 1.77E+07 | 1.47E+07 | 7.14E+03 | 1.74E+04 | 615.1 |
| Q065 | 1.78E+07 | 1.02E+07 | 3.72E+03 | 6.88E+03 | 615.8 |
| R124 | 1.79E+07 | 1.45E+07 | 5.00E+03 | 2.97E+04 | 665.0 |
| Q233 | 1.79E+07 | 1.45E+07 | 6.46E+03 | 2.42E+04 | 706.3 |
| B064 | 1.80E+07 | 1.53E+07 | 4.73E+03 | 2.25E+04 | 678.7 |
| E187 | 1.81E+07 | 1.43E+07 | 4.05E+03 | 1.69E+04 | 516.9 |
| Q132 | 1.81E+07 | 1.53E+07 | 9.87E+03 | 1.74E+04 | 475.8 |
| Q101 | 1.82E+07 | 1.56E+07 | 8.12E+03 | 1.81E+04 | 515.7 |
| B259 | 1.83E+07 | 1.33E+07 | 1.43E+04 | 8.59E+03 | 540.7 |
| R130 | 1.83E+07 | 1.55E+07 | 8.71E+03 | 1.40E+04 | 631.8 |
| B109 | 1.84E+07 | 1.54E+07 | 4.87E+03 | 2.02E+04 | 648.2 |
| B228 | 1.85E+07 | 1.56E+07 | 5.32E+03 | 3.08E+03 | 627.8 |
| R134 | 1.85E+07 | 1.56E+07 | 4.76E+03 | 1.72E+04 | 595.0 |
| R105 | 1.86E+07 | 1.56E+07 | 2.35E+04 | 9.62E+03 | 437.9 |
| R091 | 1.86E+07 | 1.51E+07 | 6.78E+03 | 1.42E+04 | 583.7 |
| R014 | 1.93E+07 | 1.13E+06 | 5.19E+03 | 3.36E+03 | 41.6 |
| Q300 | 1.97E+07 | 1.65E+07 | 8.22E+03 | 6.53E+03 | 575.8 |
| B255 | 1.97E+07 | 1.69E+07 | 3.96E+03 | 1.78E+04 | 854.2 |
| R101 | 1.98E+07 | 2.74E+06 | 8.61E+03 | 2.03E+03 | 104.2 |
| E070 | 1.98E+07 | 1.49E+07 | 5.90E+03 | 1.26E+04 | 558.6 |
| B293 | 2.00E+07 | 1.50E+07 | 5.96E+03 | 1.39E+04 | 617.8 |
| B312 | 2.01E+07 | 1.54E+07 | 1.03E+04 | 1.02E+04 | 511.1 |
| Q308 | 2.02E+07 | 1.72E+07 | 6.58E+03 | 1.77E+04 | 720.0 |
| R085 | 2.03E+07 | 1.57E+07 | 5.78E+03 | 1.47E+04 | 636.0 |
| Q056 | 2.03E+07 | 1.25E+07 | 8.52E+03 | 6.97E+03 | 503.8 |
| R025 | 2.03E+07 | 1.79E+07 | 1.32E+04 | 4.39E+03 | 887.8 |
| Q245 | 2.05E+07 | 1.74E+07 | 1.28E+04 | 1.87E+04 | 852.2 |
| R135 | 2.10E+07 | 7.78E+06 | 6.19E+03 | 9.23E+03 | 317.2 |
| R097 | 2.10E+07 | 1.10E+06 | 5.06E+03 | 2.83E+03 | 45.2 |
| Q071 | 2.10E+07 | 1.77E+07 | 4.79E+03 | 1.45E+04 | 773.8 |
| R055 | 2.11E+07 | 1.64E+07 | 3.38E+03 | 1.35E+04 | 842.1 |
| Q051 | 2.11E+07 | 1.61E+06 | 2.97E+03 | 2.39E+03 | 47.0 |
| Q196 | 2.11E+07 | 2.03E+06 | 8.22E+03 | 1.95E+03 | 69.1 |
| E130 | 2.12E+07 | 1.33E+07 | 4.05E+03 | 2.12E+04 | 384.4 |
| E124 | 2.17E+07 | 1.83E+07 | 8.36E+03 | 2.23E+04 | 575.8 |
| E132 | 2.19E+07 | 1.28E+07 | 4.83E+03 | 6.08E+03 | 454.2 |
| R126 | 2.24E+07 | 7.05E+05 | 1.77E+03 | 1.45E+03 | 23.9 |
| Q199 | 2.27E+07 | 1.69E+07 | 1.29E+04 | 5.50E+03 | 508.4 |
| Q087 | 2.28E+07 | 1.42E+07 | 6.57E+03 | 1.24E+04 | 850.0 |
| B045 | 2.30E+07 | 1.96E+07 | 1.24E+04 | 1.10E+04 | 735.7 |
| E082 | 2.42E+07 | 1.87E+07 | 5.65E+03 | 2.46E+04 | 885.0 |
| Q312 | 2.43E+07 | 1.53E+07 | 2.64E+04 | 6.15E+03 | 468.0 |
| R119 | 2.43E+07 | 1.57E+07 | 5.31E+03 | 2.55E+04 | 629.5 |
| B200 | 2.50E+07 | 2.06E+07 | 7.17E+03 | 2.81E+04 | 892.9 |
| B147 | 2.59E+07 | 1.69E+07 | 6.66E+03 | 1.21E+04 | 568.0 |
| E055 | 2.62E+07 | 2.06E+07 | 5.57E+03 | 1.34E+04 | 758.6 |
| B024 | 2.63E+07 | 2.13E+07 | 8.10E+03 | 1.21E+04 | 935.9 |
| R084 | 2.63E+07 | 2.05E+07 | 5.46E+03 | 1.93E+04 | 701.0 |
| E119 | 2.65E+07 | 2.01E+07 | 1.71E+04 | 1.00E+04 | 692.6 |
| Q294 | 2.66E+07 | 2.22E+07 | 1.57E+04 | 1.28E+04 | 763.7 |
| Q253 | 2.95E+07 | 2.42E+07 | 8.91E+03 | 2.68E+04 | 1134.3 |

| Supplementary Table 5. Isolate whole genome assembly metrics | | | | |
| --- | --- | --- | --- | --- |
| Isolate ID | Isolate genome assembly contigs <500 bp | Isolate genome assembly N50 | Isolate genome assembly size (bp) | Isolate estimated sequencing depth (X) |
| B001_5 | 257 | 6.18E+04 | 5.14E+06 | 53 |
| B100_5 | 109 | 1.59E+05 | 5.15E+06 | 38 |
| B101_1 | 208 | 7.09E+04 | 5.26E+06 | 40 |
| B109_1 | 403 | 2.20E+04 | 4.73E+06 | 17 |
| B118_2 | 812 | 1.27E+04 | 5.44E+06 | 40 |
| B119_1 | 213 | 8.08E+04 | 5.09E+06 | 45 |
| B12_1 | 155 | 1.14E+05 | 4.98E+06 | 56 |
| B141_2 | 236 | 6.04E+04 | 5.06E+06 | 288 |
| B143_4 | 393 | 2.48E+04 | 4.77E+06 | 43 |
| B145_4 | 182 | 1.07E+05 | 4.80E+06 | 25 |
| B147_1 | 301 | 4.41E+04 | 5.29E+06 | 303 |
| B169_1 | 234 | 5.85E+04 | 5.24E+06 | 23 |
| B188_1 | 426 | 2.25E+04 | 5.04E+06 | 16 |
| B200_2 | 243 | 4.29E+04 | 4.80E+06 | 20 |
| B201_3 | 193 | 6.34E+04 | 5.02E+06 | 57 |
| B201_5 | 228 | 5.63E+04 | 4.95E+06 | 32 |
| B202_2 | 186 | 9.29E+04 | 4.94E+06 | 45 |
| B207_2 | 204 | 6.15E+04 | 4.96E+06 | 98 |
| B225_3 | 225 | 7.57E+04 | 4.88E+06 | 396 |
| B226_1 | 206 | 7.92E+04 | 5.15E+06 | 228 |
| B231_1 | 219 | 5.67E+04 | 5.01E+06 | 57 |
| B231_2 | 236 | 5.53E+04 | 4.69E+06 | 26 |
| B234_1 | 481 | 3.77E+04 | 5.28E+06 | 464 |
| B235_3 | 183 | 9.05E+04 | 5.02E+06 | 132 |
| B24_1 | 253 | 5.52E+04 | 4.81E+06 | 162 |
| B244_3 | 201 | 6.61E+04 | 4.85E+06 | 289 |
| B246_1 | 250 | 7.34E+04 | 5.09E+06 | 38 |
| B255_1 | 246 | 4.95E+04 | 4.72E+06 | 167 |
| B259_1 | 139 | 1.02E+05 | 4.83E+06 | 37 |
| B262_1 | 641 | 1.10E+04 | 4.45E+06 | 25 |
| B270_2 | 317 | 3.70E+04 | 4.93E+06 | 161 |
| B274_2 | 254 | 5.91E+04 | 4.92E+06 | 335 |
| B280_4 | 80 | 1.37E+05 | 4.69E+06 | 262 |
| B295_2 | 255 | 4.95E+04 | 4.72E+06 | 118 |
| B309_1 | 416 | 2.04E+04 | 4.57E+06 | 98 |
| B312_4 | 506 | 1.85E+04 | 4.82E+06 | 17 |
| B329_2 | 218 | 7.18E+04 | 5.12E+06 | 108 |
| B45_2 | 315 | 3.06E+04 | 4.85E+06 | 26 |
| B46_1 | 757 | 9.76E+03 | 4.71E+06 | 14 |
| B62_5 | 274 | 4.37E+04 | 4.88E+06 | 140 |
| B66_1 | 207 | 7.22E+04 | 5.07E+06 | 48 |
| B66_4 | 160 | 9.75E+04 | 5.04E+06 | 51 |
| B75_4 | 168 | 7.47E+04 | 5.15E+06 | 284 |
| B75_5 | 285 | 7.24E+04 | 5.42E+06 | 213 |
| B84_2 | 244 | 5.51E+04 | 4.75E+06 | 22 |
| B84_3 | 380 | 2.35E+04 | 4.86E+06 | 66 |
| B88_3 | 411 | 2.59E+04 | 5.07E+06 | 45 |
| B95_1 | 219 | 7.54E+04 | 5.25E+06 | 441 |
| B99_5 | 335 | 3.90E+04 | 5.09E+06 | 401 |
| C1_2 | 143 | 9.83E+04 | 4.81E+06 | 41 |
| C101_1 | 138 | 9.82E+04 | 4.85E+06 | 38 |
| C102_1 | 1115 | 7.11E+03 | 5.09E+06 | 52 |
| C110_5 | 315 | 3.88E+04 | 4.86E+06 | 31 |
| C14_2A | 272 | 5.13E+04 | 5.41E+06 | 26 |
| C14_2B | 332 | 5.36E+04 | 5.27E+06 | 26 |
| C21_2 | 254 | 5.42E+04 | 5.05E+06 | 97 |
| C23_1 | 498 | 1.81E+04 | 4.75E+06 | 30 |
| C25_2 | 146 | 9.77E+04 | 4.94E+06 | 52 |
| C26_2 | 305 | 4.00E+04 | 5.02E+06 | 48 |
| C33_3 | 931 | 7.38E+03 | 4.59E+06 | 16 |
| C34_2 | 178 | 8.54E+04 | 4.87E+06 | 11 |
| C36_1 | 271 | 5.65E+04 | 5.11E+06 | 31 |
| C46_4 | 195 | 6.09E+04 | 5.10E+06 | 25 |
| C50_4 | 117 | 1.03E+05 | 4.82E+06 | 27 |
| C70_1 | 330 | 5.40E+04 | 5.26E+06 | 49 |
| C71_2 | 238 | 5.05E+04 | 5.39E+06 | 30 |
| C72_3 | 293 | 5.28E+04 | 5.07E+06 | 69 |
| C80_4 | 193 | 7.03E+04 | 5.01E+06 | 77 |
| C9_2 | 205 | 6.52E+04 | 5.15E+06 | 54 |
| E100_4 | 233 | 5.20E+04 | 5.29E+06 | 146 |
| E100_5 | 238 | 7.08E+04 | 5.04E+06 | 206 |
| E101_1 | 182 | 9.21E+04 | 5.14E+06 | 109 |
| E106_1 | 231 | 7.02E+04 | 5.07E+06 | 109 |
| E119_5 | 204 | 8.67E+04 | 5.17E+06 | 211 |
| E124_5 | 231 | 5.11E+04 | 5.11E+06 | 53 |
| E124_6 | 278 | 4.80E+04 | 5.15E+06 | 90 |
| E129_3 | 229 | 4.04E+04 | 4.96E+06 | 85 |
| E13 | 881 | 8.44E+03 | 5.03E+06 | 48 |
| E135_2 | 123 | 1.12E+05 | 5.04E+06 | 53 |
| E135_5 | 295 | 4.92E+04 | 5.24E+06 | 31 |
| E135_6 | 370 | 2.96E+04 | 5.19E+06 | 36 |
| E139_2 | 368 | 2.70E+04 | 4.91E+06 | 16 |
| E140 | 205 | 6.34E+04 | 5.12E+06 | 36 |
| E144 | 160 | 8.08E+04 | 4.75E+06 | 58 |
| E158 | 202 | 7.70E+04 | 5.20E+06 | 42 |
| E16_6 | 332 | 4.04E+04 | 5.50E+06 | 191 |
| E162 | 148 | 8.96E+04 | 4.76E+06 | 38 |
| E166 | 282 | 4.66E+04 | 5.39E+06 | 31 |
| E167_5 | 188 | 7.65E+04 | 5.05E+06 | 165 |
| E170 | 592 | 1.45E+04 | 5.06E+06 | 12 |
| E173 | 234 | 6.19E+04 | 5.23E+06 | 83 |
| E175_4 | 170 | 7.04E+04 | 4.90E+06 | 211 |
| E177_6 | 286 | 3.59E+04 | 5.11E+06 | 122 |
| E187_2 | 298 | 4.20E+04 | 5.04E+06 | 38 |
| E188_2 | 313 | 3.26E+04 | 5.29E+06 | 24 |
| E192 | 228 | 7.51E+04 | 5.26E+06 | 38 |
| E195_5 | 110 | 1.33E+05 | 4.85E+06 | 39 |
| E197_5 | 341 | 3.87E+04 | 5.47E+06 | 92 |
| E20_1 | 229 | 6.64E+04 | 5.04E+06 | 114 |
| E212_4 | 1093 | 2.47E+04 | 6.43E+06 | 46 |
| E218 | 245 | 5.03E+04 | 5.25E+06 | 25 |
| E228_3 | 269 | 5.71E+04 | 5.43E+06 | 114 |
| E230_4 | 1630 | 5.05E+03 | 5.88E+06 | 40 |
| E236_4 | 331 | 2.84E+04 | 4.72E+06 | 35 |
| E238_3 | 584 | 1.53E+04 | 4.88E+06 | 1077 |
| E238_5 | 356 | 2.67E+04 | 5.02E+06 | 1104 |
| E240_5 | 133 | 8.40E+04 | 4.79E+06 | 647 |
| E26 | 206 | 1.06E+05 | 5.32E+06 | 47 |
| E28 | 224 | 5.55E+04 | 5.17E+06 | 36 |
| E29_1 | 280 | 3.74E+04 | 4.91E+06 | 40 |
| E33_4 | 131 | 1.28E+05 | 4.90E+06 | 45 |
| E34 | 197 | 8.21E+04 | 5.20E+06 | 30 |
| E43 | 223 | 7.51E+04 | 5.11E+06 | 13 |
| E44_6 | 323 | 3.49E+04 | 5.09E+06 | 139 |
| E5_5 | 556 | 1.68E+04 | 5.09E+06 | 47 |
| E55_5 | 253 | 5.89E+04 | 5.27E+06 | 548 |
| E55_6 | 379 | 2.96E+04 | 4.88E+06 | 433 |
| E57 | 217 | 6.48E+04 | 5.05E+06 | 38 |
| E58_3 | 414 | 2.59E+04 | 5.03E+06 | 978 |
| E67_5 | 528 | 1.60E+04 | 4.96E+06 | 439 |
| E70_3 | 1591 | 3.05E+03 | 4.18E+06 | 250 |
| E71 | 106 | 8.91E+04 | 4.68E+06 | 44 |
| E72 | 408 | 2.34E+04 | 5.24E+06 | 7 |
| E76_1 | 280 | 4.84E+04 | 5.21E+06 | 678 |
| E88_3 | 267 | 4.43E+04 | 5.07E+06 | 372 |
| E88_4 | 266 | 5.43E+04 | 4.76E+06 | 1048 |
| E89_4 | 216 | 6.37E+04 | 4.77E+06 | 501 |
| E92_5 | 456 | 3.22E+04 | 5.33E+06 | 1731 |
| Q106 | 247 | 3.96E+04 | 4.92E+06 | 22 |
| Q108 | 410 | 2.13E+04 | 4.82E+06 | 104 |
| Q128 | 452 | 1.90E+04 | 4.92E+06 | 81 |
| Q132 | 887 | 9.99E+03 | 5.19E+06 | 33 |
| Q142 | 154 | 8.04E+04 | 5.05E+06 | 48 |
| Q145 | 246 | 5.13E+04 | 5.12E+06 | 52 |
| Q147 | 318 | 3.65E+04 | 5.07E+06 | 22 |
| Q16_1 | 380 | 3.02E+04 | 5.09E+06 | 37 |
| Q186 | 253 | 5.93E+04 | 5.15E+06 | 37 |
| Q196 | 309 | 3.42E+04 | 5.19E+06 | 31 |
| Q199 | 211 | 8.09E+04 | 5.01E+06 | 60 |
| Q21_2 | 344 | 3.21E+04 | 5.28E+06 | 66 |
| Q212 | 151 | 1.07E+05 | 4.88E+06 | 44 |
| Q223 | 343 | 2.92E+04 | 4.98E+06 | 19 |
| Q23_1 | 472 | 1.84E+04 | 4.66E+06 | 85 |
| Q233 | 625 | 1.34E+04 | 4.85E+06 | 18 |
| Q240 | 290 | 4.04E+04 | 5.13E+06 | 47 |
| Q243 | 284 | 4.54E+04 | 5.27E+06 | 54 |
| Q245_2 | 762 | 1.42E+04 | 5.29E+06 | 258 |
| Q249 | 222 | 6.98E+04 | 4.97E+06 | 110 |
| Q250_1 | 333 | 3.10E+04 | 5.17E+06 | 166 |
| Q253 | 158 | 8.53E+04 | 4.83E+06 | 32 |
| Q27_1 | 205 | 5.59E+04 | 5.02E+06 | 45 |
| Q275 | 185 | 6.96E+04 | 4.92E+06 | 34 |
| Q279_1 | 499 | 1.64E+04 | 4.77E+06 | 112 |
| Q282 | 1117 | 7.65E+03 | 5.26E+06 | 26 |
| Q288 | 231 | 4.96E+04 | 4.90E+06 | 43 |
| Q294 | 215 | 5.32E+04 | 4.84E+06 | 55 |
| Q295 | 615 | 1.58E+04 | 5.31E+06 | 26 |
| Q307_1 | 281 | 3.85E+04 | 5.05E+06 | 189 |
| Q308 | 192 | 6.62E+04 | 4.90E+06 | 54 |
| Q31_6 | 955 | 7.16E+03 | 4.61E+06 | 96 |
| Q310 | 522 | 1.41E+04 | 4.38E+06 | 51 |
| Q312_1 | 205 | 5.90E+04 | 5.07E+06 | 124 |
| Q33_6 | 446 | 1.76E+04 | 4.42E+06 | 106 |
| Q34_6 | 388 | 2.09E+04 | 4.46E+06 | 167 |
| Q35_1 | 269 | 4.76E+04 | 5.08E+06 | 44 |
| Q37_4 | 433 | 2.75E+04 | 5.07E+06 | 43 |
| Q38_1 | 717 | 1.33E+04 | 5.03E+06 | 114 |
| Q40 | 386 | 2.59E+04 | 5.00E+06 | 23 |
| Q51 | 252 | 5.54E+04 | 5.20E+06 | 24 |
| Q53 | 1612 | 4.30E+03 | 5.21E+06 | 18 |
| Q56 | 137 | 1.18E+05 | 5.07E+06 | 28 |
| Q65 | 1222 | 6.47E+03 | 5.16E+06 | 38 |
| Q85_1 | 216 | 7.37E+04 | 5.22E+06 | 237 |
| Q86 | 260 | 4.03E+04 | 4.66E+06 | 110 |
| Q87 | 206 | 5.80E+04 | 4.93E+06 | 31 |
| Q89 | 695 | 1.10E+04 | 4.69E+06 | 12 |
| Q91 | 277 | 3.27E+04 | 4.66E+06 | 87 |
| R113_1 | 225 | 7.66E+04 | 5.19E+06 | 26 |
| R113_3 | 232 | 6.00E+04 | 4.91E+06 | 218 |
| R116_2 | 258 | 4.13E+04 | 4.85E+06 | 29 |
| R119_3 | 254 | 4.92E+04 | 4.71E+06 | 41 |
| R122_4 | 239 | 6.29E+04 | 4.83E+06 | 34 |
| R127_3 | 221 | 7.26E+04 | 4.86E+06 | 83 |
| R17_2 | 247 | 5.16E+04 | 4.84E+06 | 45 |
| R36_1 | 203 | 8.91E+04 | 5.23E+06 | 52 |
| R42_2 | 203 | 9.02E+04 | 5.15E+06 | 37 |
| R43_2 | 256 | 5.33E+04 | 4.91E+06 | 33 |
| R46_3 | 126 | 1.21E+05 | 4.92E+06 | 41 |
| R55_1 | 148 | 8.60E+04 | 4.89E+06 | 32 |
| R56_3 | 743 | 1.56E+04 | 5.67E+06 | 20 |
| R66_4 | 215 | 6.92E+04 | 4.89E+06 | 44 |
| R67_3 | 270 | 4.73E+04 | 5.01E+06 | 25 |
| R8_4 | 228 | 6.76E+04 | 5.03E+06 | 56 |
| R83_3 | 292 | 4.07E+04 | 5.08E+06 | 93 |
| R85_2 | 186 | 6.37E+04 | 4.70E+06 | 72 |
| R86_1 | 241 | 1.27E+05 | 5.31E+06 | 38 |

| Supplementary Table 6. 16S rRNA gene amplicon gene read numbers | | | | | | | |
| --- | --- | --- | --- | --- | --- | --- | --- |
| Sample ID | 16S rRNA gene amplicon gene reads (QC trimmed and joined) |  | Sample ID | 16S rRNA gene amplicon reads (QC trimmed and joined) |  | Sample ID | 16S rRNA gene amplicon reads (QC trimmed and joined) |
| B001 | 4.63E+04 |  | C036 | 3.38E+04 |  | Q168 | 1.12E+04 |
| B006 | 2.45E+04 |  | C038 | 3.76E+04 |  | Q169 | 1.93E+04 |
| B011 | 6.29E+04 |  | C046 | 4.47E+04 |  | Q170 | 2.31E+04 |
| B019 | 4.37E+04 |  | C050 | 4.64E+04 |  | Q174 | 7.03E+03 |
| B022 | 6.28E+04 |  | C070 | 2.76E+04 |  | Q178 | 1.05E+04 |
| B024 | 3.48E+04 |  | C071 | 7.13E+04 |  | Q186 | 7.23E+03 |
| B027 | 1.01E+05 |  | C079 | 3.98E+04 |  | Q188 | 1.73E+04 |
| B031 | 2.65E+05 |  | C080 | 6.81E+04 |  | Q189 | 1.99E+04 |
| B032 | 3.06E+04 |  | C085 | 3.26E+04 |  | Q192 | 6.79E+03 |
| B033 | 9.93E+04 |  | C086 | 3.02E+04 |  | Q196 | 2.19E+04 |
| B036 | 4.66E+04 |  | C087 | 5.11E+04 |  | Q199 | 7.42E+03 |
| B037 | 4.30E+04 |  | C097 | 4.16E+04 |  | Q203 | 2.11E+04 |
| B042 | 8.17E+03 |  | C099 | 4.24E+04 |  | Q207 | 3.62E+04 |
| B045 | 5.78E+04 |  | C100 | 3.95E+04 |  | Q212 | 2.24E+04 |
| B046 | 4.68E+04 |  | C102 | 4.63E+04 |  | Q215 | 1.03E+04 |
| B047 | 1.16E+04 |  | C110 | 4.94E+04 |  | Q223 | 2.46E+04 |
| B048 | 1.48E+04 |  | E012 | 2.28E+04 |  | Q227 | 5.25E+04 |
| B056 | 2.58E+04 |  | E013 | 7.88E+04 |  | Q233 | 3.52E+04 |
| B062 | 6.24E+04 |  | E014 | 4.29E+04 |  | Q239 | 6.48E+03 |
| B064 | 5.14E+04 |  | E016 | 2.23E+04 |  | Q240 | 2.34E+04 |
| B068 | 3.83E+04 |  | E017 | 7.69E+04 |  | Q243 | 9.77E+03 |
| B069 | 4.12E+04 |  | E018 | 1.97E+05 |  | Q245 | 1.33E+05 |
| B077 | 5.26E+04 |  | E021 | 5.04E+04 |  | Q249 | 2.85E+04 |
| B084 | 4.31E+04 |  | E023 | 3.56E+04 |  | Q253 | 6.61E+03 |
| B088 | 4.94E+04 |  | E026 | 4.40E+04 |  | Q259 | 2.26E+04 |
| B089 | 3.99E+04 |  | E027 | 1.12E+04 |  | Q270 | 1.92E+04 |
| B091 | 2.52E+04 |  | E028 | 4.56E+04 |  | Q275 | 9.09E+04 |
| B095 | 2.60E+04 |  | E033 | 2.86E+05 |  | Q282 | 2.20E+04 |
| B097 | 6.33E+04 |  | E034 | 4.56E+04 |  | Q284 | 3.41E+05 |
| B099 | 3.61E+04 |  | E036 | 5.58E+04 |  | Q288 | 5.72E+04 |
| B100 | 1.27E+05 |  | E038 | 1.09E+05 |  | Q289 | 2.71E+04 |
| B101 | 4.72E+04 |  | E043 | 4.04E+03 |  | Q291 | 1.07E+04 |
| B103 | 7.77E+04 |  | E044 | 2.42E+04 |  | Q294 | 1.08E+05 |
| B104 | 7.88E+04 |  | E055 | 4.28E+04 |  | Q295 | 7.28E+03 |
| B105 | 3.04E+04 |  | E056 | 3.29E+04 |  | Q300 | 5.60E+04 |
| B106 | 3.25E+04 |  | E057 | 3.66E+04 |  | Q304 | 1.22E+04 |
| B108 | 5.92E+04 |  | E070 | 5.45E+04 |  | Q308 | 2.81E+04 |
| B109 | 1.97E+04 |  | E071 | 5.90E+05 |  | Q310 | 2.75E+04 |
| B112 | 4.66E+04 |  | E072 | 4.26E+04 |  | Q312 | 4.96E+03 |
| B117 | 5.79E+04 |  | E076 | 5.80E+04 |  | R001 | 1.62E+04 |
| B118 | 2.22E+04 |  | E082 | 2.70E+04 |  | R003 | 7.88E+03 |
| B119 | 4.87E+04 |  | E084 | 2.83E+04 |  | R006 | 2.02E+04 |
| B122 | 1.46E+04 |  | E085 | 5.88E+04 |  | R008 | 4.98E+04 |
| B124 | 5.98E+04 |  | E093 | 4.77E+04 |  | R009 | 1.99E+04 |
| B126 | 2.51E+04 |  | E108 | 6.92E+04 |  | R010 | 1.25E+05 |
| B129 | 1.53E+04 |  | E114 | 1.13E+05 |  | R011 | 2.54E+05 |
| B135 | 9.69E+04 |  | E115 | 1.06E+04 |  | R012 | 4.40E+04 |
| B141 | 8.07E+04 |  | E119 | 1.55E+05 |  | R013 | 9.30E+04 |
| B143 | 1.62E+05 |  | E124 | 3.77E+04 |  | R014 | 2.36E+04 |
| B145 | 8.18E+04 |  | E130 | 1.28E+04 |  | R015 | 8.99E+04 |
| B147 | 8.85E+04 |  | E131 | 1.34E+05 |  | R017 | 3.73E+04 |
| B159 | 1.45E+04 |  | E132 | 2.02E+05 |  | R021 | 1.38E+04 |
| B165 | 2.40E+04 |  | E135 | 4.44E+04 |  | R022 | 1.36E+04 |
| B170 | 4.32E+04 |  | E139 | 8.52E+04 |  | R024 | 1.83E+04 |
| B174 | 3.65E+04 |  | E140 | 1.02E+05 |  | R025 | 2.95E+04 |
| B181 | 1.89E+04 |  | E141 | 2.54E+04 |  | R026 | 3.56E+05 |
| B185 | 1.94E+04 |  | E143 | 2.22E+04 |  | R029 | 2.46E+04 |
| B188 | 5.32E+04 |  | E144 | 4.83E+04 |  | R030 | 7.16E+04 |
| B192 | 3.62E+04 |  | E153 | 2.13E+05 |  | R031 | 4.73E+04 |
| B194 | 4.99E+04 |  | E158 | 8.23E+03 |  | R032 | 2.32E+04 |
| B195 | 1.44E+04 |  | E162 | 8.53E+03 |  | R039 | 3.60E+05 |
| B196 | 6.10E+04 |  | E166 | 1.94E+04 |  | R040 | 1.22E+04 |
| B200 | 4.97E+04 |  | E167 | 1.82E+04 |  | R041 | 1.68E+05 |
| B201 | 2.71E+04 |  | E170 | 1.50E+04 |  | R042 | 5.88E+04 |
| B202 | 1.46E+04 |  | E171 | 5.65E+03 |  | R043 | 3.84E+04 |
| B203 | 1.96E+04 |  | E173 | 3.12E+04 |  | R044 | 2.03E+04 |
| B207 | 3.22E+04 |  | E175 | 2.12E+04 |  | R045 | 3.27E+04 |
| B209 | 4.43E+04 |  | E177 | 7.82E+03 |  | R046 | 7.34E+03 |
| B210 | 3.77E+04 |  | E184 | 9.72E+04 |  | R050 | 3.79E+04 |
| B211 | 2.73E+04 |  | E185 | 8.07E+04 |  | R051 | 2.18E+04 |
| B212 | 2.16E+04 |  | E187 | 2.96E+04 |  | R052 | 2.26E+04 |
| B213 | 4.55E+04 |  | E188 | 3.05E+04 |  | R053 | 3.34E+04 |
| B214 | 1.40E+05 |  | E189 | 6.19E+04 |  | R054 | 1.99E+04 |
| B215 | 1.41E+05 |  | E192 | 1.93E+04 |  | R055 | 4.43E+04 |
| B216 | 1.58E+04 |  | E194 | 3.00E+04 |  | R056 | 3.54E+04 |
| B217 | 8.85E+03 |  | E196 | 2.55E+04 |  | R057 | 1.69E+04 |
| B219 | 4.14E+04 |  | E204 | 7.19E+04 |  | R058 | 4.15E+04 |
| B226 | 4.46E+04 |  | E205 | 7.41E+04 |  | R059 | 7.93E+04 |
| B228 | 6.21E+04 |  | E206 | 9.48E+04 |  | R060 | 1.55E+04 |
| B230 | 1.01E+04 |  | E212 | 3.22E+04 |  | R061 | 5.64E+04 |
| B231 | 2.27E+04 |  | E215 | 4.74E+04 |  | R062 | 6.58E+04 |
| B234 | 6.90E+04 |  | E218 | 8.90E+04 |  | R063 | 6.93E+04 |
| B235 | 3.11E+04 |  | E228 | 1.97E+04 |  | R064 | 1.71E+04 |
| B236 | 1.22E+04 |  | E230 | 6.69E+04 |  | R065 | 2.81E+04 |
| B237 | 4.82E+04 |  | E233 | 6.09E+04 |  | R066 | 4.66E+04 |
| B244 | 4.71E+04 |  | E236 | 4.32E+04 |  | R067 | 9.44E+04 |
| B245 | 2.80E+04 |  | Q040 | 8.62E+03 |  | R068 | 4.18E+04 |
| B246 | 1.02E+04 |  | Q049 | 9.69E+03 |  | R071 | 1.42E+04 |
| B248 | 2.23E+04 |  | Q051 | 2.22E+04 |  | R074 | 1.23E+04 |
| B249 | 1.03E+05 |  | Q053 | 2.87E+04 |  | R076 | 1.30E+04 |
| B250 | 6.35E+04 |  | Q056 | 5.31E+03 |  | R077 | 1.53E+04 |
| B251 | 2.80E+04 |  | Q057 | 3.93E+03 |  | R078 | 4.54E+04 |
| B252 | 1.03E+04 |  | Q061 | 3.34E+04 |  | R079 | 6.10E+04 |
| B253 | 3.52E+05 |  | Q065 | 1.47E+04 |  | R080 | 1.13E+05 |
| B255 | 4.95E+04 |  | Q069 | 1.11E+04 |  | R081 | 5.52E+04 |
| B259 | 4.14E+04 |  | Q070 | 6.29E+03 |  | R083 | 2.87E+04 |
| B263 | 3.85E+05 |  | Q071 | 8.64E+03 |  | R084 | 6.67E+04 |
| B270 | 4.87E+04 |  | Q074 | 2.47E+04 |  | R085 | 5.90E+04 |
| B273 | 1.48E+05 |  | Q083 | 6.69E+03 |  | R088 | 2.01E+04 |
| B274 | 4.93E+04 |  | Q086 | 6.76E+04 |  | R090 | 1.55E+04 |
| B276 | 2.21E+04 |  | Q087 | 4.79E+03 |  | R091 | 1.38E+05 |
| B278 | 8.40E+03 |  | Q089 | 8.02E+03 |  | R093 | 8.25E+04 |
| B279 | 4.12E+04 |  | Q090 | 3.26E+04 |  | R097 | 1.15E+04 |
| B280 | 1.50E+05 |  | Q091 | 6.23E+03 |  | R098 | 2.81E+04 |
| B281 | 9.55E+03 |  | Q092 | 1.37E+04 |  | R101 | 2.74E+04 |
| B282 | 1.07E+04 |  | Q097 | 9.55E+03 |  | R102 | 3.00E+04 |
| B283 | 6.76E+04 |  | Q098 | 1.47E+04 |  | R104 | 1.17E+05 |
| B285 | 8.68E+04 |  | Q099 | 1.52E+04 |  | R105 | 2.41E+05 |
| B286 | 5.34E+04 |  | Q101 | 2.01E+04 |  | R109 | 5.37E+04 |
| B293 | 4.16E+04 |  | Q104 | 5.26E+03 |  | R110 | 1.63E+04 |
| B295 | 5.75E+04 |  | Q105 | 5.25E+04 |  | R111 | 2.06E+04 |
| B302 | 5.81E+04 |  | Q106 | 2.34E+04 |  | R113 | 8.83E+04 |
| B305 | 6.30E+04 |  | Q107 | 1.46E+04 |  | R114 | 2.13E+04 |
| B308 | 1.27E+04 |  | Q108 | 2.14E+04 |  | R116 | 2.65E+04 |
| B309 | 2.74E+04 |  | Q116 | 1.17E+04 |  | R118 | 1.56E+04 |
| B311 | 5.65E+04 |  | Q117 | 2.57E+04 |  | R119 | 7.46E+04 |
| B312 | 3.69E+04 |  | Q127 | 3.38E+04 |  | R120 | 5.82E+04 |
| B313 | 3.19E+04 |  | Q128 | 4.62E+04 |  | R122 | 4.16E+04 |
| B315 | 9.43E+03 |  | Q130 | 2.26E+04 |  | R123 | 1.56E+04 |
| B323 | 4.54E+04 |  | Q131 | 1.22E+04 |  | R124 | 1.38E+04 |
| B327 | 3.20E+04 |  | Q132 | 8.56E+03 |  | R125 | 1.79E+04 |
| B328 | 1.88E+04 |  | Q133 | 5.16E+03 |  | R126 | 2.27E+04 |
| B329 | 4.24E+04 |  | Q139 | 1.58E+04 |  | R127 | 1.94E+04 |
| C009 | 6.15E+04 |  | Q142 | 1.39E+04 |  | R128 | 2.84E+04 |
| C013 | 4.90E+04 |  | Q143 | 5.27E+03 |  | R129 | 2.39E+04 |
| C014 | 6.20E+04 |  | Q144 | 3.95E+03 |  | R130 | 3.37E+04 |
| C019 | 4.23E+04 |  | Q145 | 6.34E+03 |  | R131 | 1.14E+04 |
| C021 | 3.40E+04 |  | Q146 | 4.63E+04 |  | R132 | 6.21E+04 |
| C023 | 5.32E+04 |  | Q147 | 7.25E+04 |  | R134 | 1.69E+04 |
| C025 | 5.78E+04 |  | Q148 | 7.68E+03 |  | R135 | 3.38E+04 |
| C026 | 4.01E+04 |  | Q157 | 1.05E+04 |  | R136 | 2.41E+05 |
| C032 | 6.18E+04 |  | Q158 | 6.76E+03 |  | R137 | 4.59E+04 |
| C033 | 3.37E+04 |  | Q159 | 1.68E+04 |  | R138 | 2.57E+04 |
| C034 | 5.54E+04 |  | Q160 | 1.38E+04 |  |  |  |

| Supplementary Table 7. Pathotype designation criteria and reference gene sequences used for the read-based whole genome scan of presumptive DEC isolates. | | | | |
| --- | --- | --- | --- | --- |
| Pathotype | Pathotype designation criteria^12^ | Gene name | Gene description | Genbank Accession |
| ETEC | Isolates were designated as ETEC if either or both the *lt* (both subunits) or *sta* genes were present | *eltA* | Heat-labile enterotoxin subunits A and B | AAA24685 |
|  |  | *eltB* |  | AAA98064 |
|  |  | *sta* | Heat-stable enterotoxin | WP_001353651 |
| EPEC | tEPEC were defined by the presence of both *eaeA* and *bfpA*; aEPEC were defined by the presence of *eaeA* and absence of *bfpA* | *eaeA* | Locus of enterocyte effacement-encoded intimin protein | AAC38392 |
|  |  | *bfpA* | Bundle-forming pilus | BAA84838 |
| EAEC | Isolates were defined as EAEC if *aggR*, *aaiC*, and/or *aatA* were present | *aggR* | Aggregative adherence transcriptional regulator | QKN22446 |
|  |  | *aaiC* | Secreted protein | LT719075 |
|  |  | *aatA* | Biofilm formation protein | SJK83517 |
| DAEC^1^ | The afa, dra, and daa operons are made up of a series of structural proteins and adhesins; isolates were considered DAEC if their draft genome sequences contained >50% of structural genes for at least one operon and 1 or more adhesin genes (*afaE, draE*, or F1845) | *afaF-III* | afa operon transcriptional regulator | CAA54112 |
|  |  | *afaA* | afa operon transcriptional regulator | CAW30797 |
|  |  | *afaB-I* | Afa operon periplasmic chaperone | CAW30798 |
|  |  | *afaC-I* | Afa operon anchoring protein variant | CAW30799 |
|  |  | *afaC-III* | Afa operon anchoring protein variant | CAA54117 |
|  |  | *afaD* | Afibrial adhesin subunit D | CAW30800 |
|  |  | *afaE-I* | Afimbrial adhesin subunit E variant | CAW30801 |
|  |  | *afaE-II* | Afimbrial adhesin subunit E variant | CAA59767 |
|  |  | *afaE-III* | Afimbrial adhesin subunit E variant | CAA54121 |
|  |  | *afaE-V* | Afimbrial adhesin subunit E variant | CAA62863 |
|  |  | *draA* | Dra operon transcriptional regulator | AAK16475 |
|  |  | *draB* | Dra operon periplasmic chaperone | AAK16476 |
|  |  | *draC* | Dra operon anchoring protein | AAK16477 |
|  |  | *draD* | Dra operon afibrial adhesin subunit D | AAK16478 |
|  |  | *draP* | Dra operon fimbriae coding cluster protein | AAK16479 |
|  |  | *draE* | Dra operon fimbrial adhesin subunit E variant | AAK16480 |
|  |  | *draE-II* | Dra operon fimbrial adhesin subunit E variant | AAB65153 |
|  |  | *daaF* | Daa operon transcriptional regulator | AAA23662 |
|  |  | *daaA* | Daa operon transcriptional regulator | AAA23663 |
|  |  | *daaC* | Daa operon anchoring protiein | ABU51870 |
|  |  | *daaD* | Daa Fimbrial adhesin subunit D | AAG10405 |
|  |  | *F1845* | Daa operon fimbrial adhesin | AAA23661 |
| EIEC | *E. coli* isolates with ipaH were designated as EIEC | *ipaH* | pINV plasmid-encoded type-III effector protein | NP_858212 |
| EHEC | Isolates that had *stx* and *eaeA* were designated as EHEC | *stx1 A* | Shiga-like toxin, subunits A and B | WP_000691354 |
|  |  | *stx1 B* |  | WP_000752026 |
|  |  | *eaeA* | Locus of enterocyte effacement-encoded intimin protein | AAC38392 |

| Supplementary Table 8. qPCR primers, annealing temperature, and assay performance | | | | | | | |
| --- | --- | --- | --- | --- | --- | --- | --- |
| Target (gene) | Primer name | Primer sequence (5’ to 3’) | Annealing temp. | Mean slope | Mean y-intercept | Mean R^2^ | Reference |
| Total bacteria (16S rRNA gene) | U16SRT-F | ACTCCTACGGGAGGCAGCAGT | 61.5°C | -3.67 | 42.1 | 0.996 | Clifford *et al.* 2012^13^ |
|  | U16SRT-R | TATTACCGCGGCTGCTGGC |  |  |  |  |  |
| Total *E. coli* (uidA) | uidA-F | CGGAAGCAACGCGTAAACTC | 61.5°C | -3.60 | 41.4 | 0.996 | Silkie et al. 2008^14^ |
|  | uidA-R | TGAGCGTCGCAGAACATTACA |  |  |  |  |  |
| Inhibition control assay | ICA-F | CTAACCTTCGTGATGAGCAATCG | 60°C | -3.35 | 38.8 | 0.991 | Deer *et al.* 2010^8^ |
|  | ICA-R | GATCAGCTACGTGAGGTCCTAC |  |  |  |  |  |

| Supplementary Table 9. Alpha diversity statistics; *p*-values are for two-way nonparametric Wilcoxon tests and bold typeface indicates significance at *p*<0.05. | | | | | | | | | | | | | |
| --- | --- | --- | --- | --- | --- | --- | --- | --- | --- | --- | --- | --- | --- |
|  |  |  | 16S rRNA alpha diversity | | | | | | | |  | Shotgun metagenome alpha diversity | |
| *E. coli* ASVs/reads | Sample group |  | Mean Shannon diversity (stdev) | *p* |  | Mean Simpson diversity (stdev) | *p* |  | Mean observed ASVs (stdev) | *p* |  | Mean nonpareil N_d_ | *p* |
| Included | Symptomatic DEC infections |  | 17.05 (10.92) | 0.50 |  | 9.32 (6.21) | 1 |  | 81.02 (36.82) | **0.0018** |  | 16.49 (1.00) | 0.33 |
|  | Asymptomatic DEC infections |  | 18.51 (11.3) |  |  | 9.04 (5.41) |  |  | 109.31 (50.17) |  |  | 16.85 (0.78) |  |
|  | Uninfected cases |  | 17.29 (10.44) | 0.11 |  | 8.89 (5.67) | 0.25 |  | 100.81 (43.42) | 0.67 |  | 16.69 (0.70) | 0.057 |
|  | Uninfected controls |  | 20.74 (13.04) |  |  | 9.95 (6.07) |  |  | 107.97 (52.65) |  |  | 17.07 (0.67) |  |
| Removed | Symptomatic DEC infections |  | 17.05 (10.66) | 0.65 |  | 9.24 (6.11) | 0.85 |  | 81.88 (35.70) | **0.0044** |  | 16.22 (1.44) | 0.17 |
|  | Asymptomatic DEC infections |  | 18.14 (11.19) |  |  | 8.83 (5.33) |  |  | 107.59 (50.25) |  |  | 16.83 (0.83) |  |
|  | Uninfected cases |  | 17.24 (10.35) | 0.13 |  | 8.79 (5.60) | 0.27 |  | 101.44 (42.32) | 0.81 |  | 16.65 (0.70) | 0.065 |
|  | Uninfected controls |  | 20.49 (13.09) |  |  | 9.94 (6.43) |  |  | 107.71 (52.10) |  |  | 17.06 (0.69_ |  |

| Supplementary Table 10. Beta diversity statistics; bold typeface indicates significance at p<0.05. | | | | | | | | | |
| --- | --- | --- | --- | --- | --- | --- | --- | --- | --- |
|  |  |  | 16S rRNA Bray-Curtis dissimilarity statistics | | |  | Shotgun metagenome Mash distance statistics | | |
| *E. coli* ASVs/reads | Comparison |  | NMDS Stress | Beta dispersion | PERMANOVA |  | NMDS stress | Beta dispersion | PERMANOVA |
| Included | Symptomatic versus asymptomatic DEC infections |  | 0.21 | *p* = 0.75 | *p =* 0.13  R^2^ = 0.011 |  | 0.036 | *p* = 0.24 | *p =* 0.13  R^2^ = 0.036 |
|  |  |  |  |  |  |  |  |  |  |
|  | Uninfected cases versus controls |  | 0.21 | *p* = 0.41 | ***p =* 0.016**  R^2^ = 0.010 |  | 0.047 | *p* = 0.73 | *p =* 0.17  R^2^ = 0.032 |
|  |  |  |  |  |  |  |  |  |  |
| Removed | Symptomatic versus asymptomatic DEC infections |  | 0.21 | *p* = 0.62 | *p =* 0.19  R^2^ = 0.010 |  | 0.066 | *p* = 0.50 | *p =* 0.26  R^2^ = 0.026 |
|  |  |  |  |  |  |  |  |  |  |
|  | Uninfected cases versus controls |  | 0.21 | *p* = 0.30 | ***p =* 0.015**  R^2^ = 0.010 |  | 0.046 | ***p* = 0.0068** | *p =* 0.20  R^2^ = 0.029 |
|  |  |  |  |  |  |  |  |  |  |
|  |  |  |  |  |  |  |  |  |  |
|  |  |  |  |  |  |  |  |  |  |

**References**

1. Toma C, Lu Y, Higa N, Nakasone N, Chinen I, Baschkier A, Rivas M, Iwanaga M. Multiplex PCR assay for identification of human diarrheagenic Escherichia coli. Journal of clinical microbiology 2003; 41:2669–71.

2. Le Bouguenec C, Archambaud M, Labigne A. Rapid and specific detection of the pap, afa, and sfa adhesin-encoding operons in uropathogenic Escherichia coli strains by polymerase chain reaction. Journal of clinical microbiology 1992; 30:1189–93.

3. Tornieporth NG, John J, Salgado K, de Jesus P, Latham E, Melo MC, Gunzburg ST, Riley LW. Differentiation of pathogenic Escherichia coli strains in Brazilian children by PCR. Journal of clinical microbiology 1995; 33:1371–4.

4. Paton AW, Paton JC. Detection and characterization of Shiga toxigenic Escherichia coli by using multiplex PCR assays for stx1, stx2, eaeA, enterohemorrhagic E. coli hlyA, rfbO111, and rfbO157. Journal of clinical microbiology 1998; 36:598–602.

5. Kozich JJ, Westcott SL, Baxter NT, Highlander SK, Schloss PD. Development of a Dual-Index Sequencing Strategy and Curation Pipeline for Analyzing Amplicon Sequence Data on the MiSeq Illumina Sequencing Platform. Appl Environ Microbiol 2013; 79:5112–20.

6. Altschul SF, Gish W, Miller W, Myers EW, Lipman DJ. Basic local alignment search tool. Journal of Molecular Biology 1990; 215:403–10.

7. Rodriguez-R L, Konstantinidis K. The enveomics collection: a toolbox for specialized analyses of microbial genomes and metagenomes. 2016;

8. Deer DM, Lampel KA, González-Escalona N. A versatile internal control for use as DNA in real-time PCR and as RNA in real-time reverse transcription PCR assays. Letters in Applied Microbiology 2010; 50:366–72.

9. Bustin SA, Benes V, Garson JA, Hellemans J, Huggett J, Kubista M, Mueller R, Nolan T, Pfaffl MW, Shipley GL, et al. The MIQE Guidelines: Minimum Information for Publication of Quantitative Real-Time PCR Experiments. Clinical Chemistry 2009; 55:611–22.

10. Forootan A, Sjöback R, Björkman J, Sjögreen B, Linz L, Kubista M. Methods to determine limit of detection and limit of quantification in quantitative real-time PCR (qPCR). Biomolecular Detection and Quantification 2017; 12:1–6.

11. Nguyen KH, Senay C, Young S, Nayak B, Lobos A, Conrad J, Harwood VJ. Determination of wild animal sources of fecal indicator bacteria by microbial source tracking (MST) influences regulatory decisions. Water Research 2018; 144:424–34.

12. Jesser KJ, Levy K. Updates on defining and detecting diarrheagenic Escherichia coli pathotypes. Current Opinion in Infectious Diseases 2020; 33:372–80.

13. Clifford RJ, Milillo M, Prestwood J, Quintero R, Zurawski DV. Detection of Bacterial 16S rRNA and Identification of Four Clinically Important Bacteria by Real-Time PCR. PLoS ONE 2012; 7:48558.

14. Silkie SS, Tolcher MP, Nelson KL. Reagent decontamination to eliminate false-positives in Escherichia coli qPCR. Journal of Microbiological Methods 2008; 72:275–82.
